# Update of the EuroGuiDerm evidence‐based guideline for the treatment of acne—Short version

**DOI:** 10.1111/jdv.70331

**Published:** 2026-03-18

**Authors:** Alexander Nast, Bassel H. Al Wattar, Marie Beylot Barry, Holger Brüggemann, Zrinka Bukvić Mokos, Dawn M. Caruana, Klaus Degitz, Clio Dessinioti, Brigitte Dréno, Rieke J. B. Driessen, Harald Gollnick, Merete Hædersdal, Alexander Katoulis, Severin Läuchli, Julien Lambert, Alison M. Layton, Giuseppe Micali, Falk R. Ochsendorf, Thozhukat Sathyapalan, Katarina Zak Stangeland, Simon Francis Thomsen, Daniel Töröcsik, Antonia Pennitz

**Affiliations:** ^1^ Division of Evidence‐Based Medicine, Klinik für Dermatologie Charité ‐ Universitätsmedizin Berlin Berlin Germany; ^2^ Comprehensive Clinical Trials Unit, Institute of Clinical Trials and Methodology University College London London UK; ^3^ Beginnings Assisted Conception Unit Epsom and St Helier University Hospitals London UK; ^4^ Dermatology Department CHU Bordeaux Bordeaux France; ^5^ Université de Bordeaux Bordeaux France; ^6^ Department of Biomedicine Aarhus University Aarhus Denmark; ^7^ Department of Dermatology and Venereology, University Hospital Centre Zagreb University of Zagreb School of Medicine Zagreb Croatia; ^8^ Private Practice Valletta Malta; ^9^ Department of Dermatology and Allergy Ludwig‐Maximilian University Munich Germany; ^10^ Department of Dermatology, Andreas Sygros Hospital University of Athens Athens Greece; ^11^ INSERM, CNRS, Immunology and New Concepts in ImmunoTherapy, INCIT, UMR 1302/EMR6001 Nantes Université Nantes France; ^12^ Department of Dermatology Radboud University Medical Center Nijmegen The Netherlands; ^13^ Department of Dermatology and Venereology University of Magdeburg Magdeburg Germany; ^14^ Department of Dermatology, Bispebjerg Hospital University of Copenhagen Copenhagen Denmark; ^15^ Second Department of Dermatology and Venereology, Medical School, “Attikon” General University Hospital National and Kapodistrian University of Athens Athens Greece; ^16^ Institute of Dermatology and Venerology Stadtspital Zürich Zürich Switzerland; ^17^ Department of Dermatology, University Hospital of Antwerp University of Antwerp Antwerp Belgium; ^18^ Skin Research Centre, Hull York Medical School University of York York UK; ^19^ Department of Dermatology Harrogate and District NHS Trust Harrogate UK; ^20^ Department of Dermatology University of Catania Sicily Italy; ^21^ Department of Dermatology, Venereology and Allergology Goethe‐University Frankfurt, Universitätsmedizin Frankfurt Germany; ^22^ Department of Academic Diabetes, Endocrinology and Metabolism, Hull York Medical School University of Hull Hull UK; ^23^ Department of Dermatology and Venereology Aleris Stavanger Stavanger Norway; ^24^ Department of Dermatology, Faculty of Medicine University of Debrecen Debrecen Hungary; ^25^ ELKH‐DE Allergology Research Group Debrecen Hungary

**Keywords:** acne vulgaris, evidence‐based medicine, methods, oral administration, practice guideline, topical administration

## Abstract

This evidence‐ and consensus‐based guideline for the treatment of acne was developed in accordance with the EuroGuiDerm Guideline and Consensus Statement Development Manual. This guideline is an update of the 2016 version. This is a short summary of the full version of the EuroGuiDerm Evidence‐based Guideline for the Treatment of Acne. For the complete guideline text, detailed methods report, and comprehensive evidence report, please refer to the online full version. In this targeted update, the guideline group prioritized three key clinical questions considered most relevant for current practice:

(a) For which types of acne and patient groups should isotretinoin be recommended versus systemic antibiotics, and with what strength of recommendation? (b) What is the appropriate duration for systemic antibiotic therapy?
For which types of acne and patient groups should hormonal treatments and spironolactone be recommended, and with what strength of recommendation?
For which types of acne and patient groups should new topical treatments, including trifarotene and clascoterone, be recommended and with what strength of recommendation?

Additionally, the updated guideline provides revised recommendations regarding: safety of benzoyl peroxide (BPO), selection of systemic antibiotic therapy, treatment considerations during pregnancy, isotretinoin dosing strategies, and the use of hormonal antiandrogenic contraceptives or other combined hormonal contraceptives, as well as spironolactone. All other aspects remain unchanged from the 2016 guideline.

## Graphical abstract

The 2025 EuroGuiDerm guideline update provides evidence‐based recommendations for acne management, addressing systemic antibiotics, systemic isotretinoin, hormonal therapies including spironolactone and contraceptives, and new topical agents. Key considerations include treatment duration, safety, pregnancy‐specific guidance and minimizing antibiotic resistance, supporting personalized, effective and safe acne care across diverse patient populations.

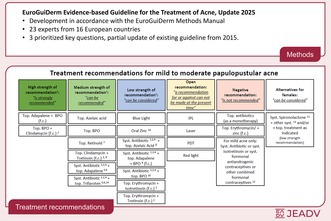

**Why was the study undertaken?**

- This guideline group identified the ‘choice between isotretinoin and systemic antibiotics’, ‘duration of systemic antibiotic treatment’ and ‘use of systemic hormonal treatments’, use of ‘clascoterone’ and ‘trifarotene’ as areas of interest.

**What does this study add?**

- It is strongly recommended to treat severe papulopustular/moderate nodular and severe nodular/conglobate acne with systemic isotretinoin (if possible).
- Use of systemic antibiotics should generally be limited to 3 months, unless specific clinical circumstances justify a longer course.
- Hormonal treatments are an alternative for females with severe acne forms as adjuncts to standard therapies.
- Spironolactone (off‐label) is an alternative for females in papulopustular, nodular or conglobate acne as an adjunct to standard treatments.
- Head‐to‐head data on clascoterone or trifarotene that would allow comparison with other topical treatments remain limited.

**What are the implications of this study for disease understanding and/or clinical care?**

- Evidence‐based selection of available acne therapies ensures effective treatment. However, additional head‐to‐head trials comparing agents such as clascoterone, trifarotene, hormonal therapies and systemic spironolactone with other treatments are needed to strengthen guideline recommendations.

## METHODS

This guideline is an update of the 2016 version. This is a short summary of the complete version of the European Evidence‐based Guideline (EuroGuiDerm) for the Treatment of Acne. For the complete guideline text, detailed methods report and comprehensive evidence report, please refer to the online full version.

In order to weigh the different recommendations, the group assigned a ‘strength of recommendation’ grade (see Table 1). The strength of recommendation considered all aspects of the treatment decision, such as efficacy, safety, patient preference and the reliability of the existing body of evidence (level of evidence).

**TABLE 1:** Strength of recommendations.

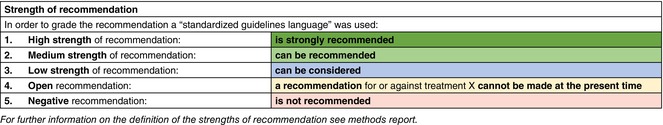

### Update 2025

This guideline is an update of the 2016 edition.^1^ For this targeted update, the group selected 3 key questions that were perceived as the most relevant questions to be dealt with in the update:

(a) ‘For which types of acne and patient groups should isotretinoin be recommended versus systemic antibiotics, and with what strength of recommendation?’ (b) ‘For what duration can treatment with systemic antibiotics be administered?’
‘For which types of acne and patient groups should hormonal treatments and spironolactone be recommended, and with what strength of recommendation?’
‘For which types of acne and patient groups should the new acne treatments trifarotene and clascoterone be recommended, and with what strength of recommendation?’

## INDUCTION THERAPY

Table 2 presents the summary of therapeutic recommendations for induction therapy. The recommendations are based on available evidence and expert consensus. Available evidence and expert voting lead to the classification of the strength of the recommendation. For recommendations on acne severity grading, see the respective chapter in the full version of the guideline (Data S1).

**TABLE 2:** Summary of therapeutic recommendations^1^ for induction therapy.

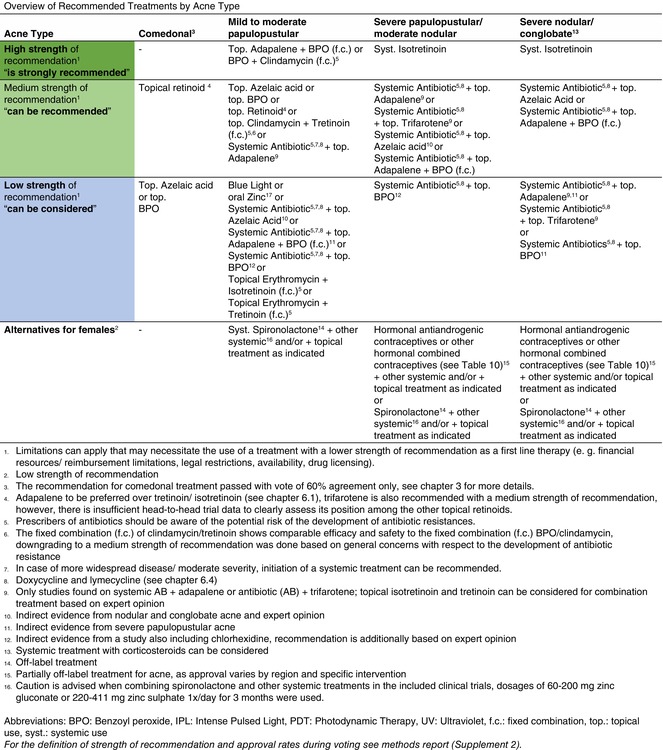

## TREATMENT OF COMEDONAL ACNE

### Recommendations for comedonal acne

**FIGURE 1:**
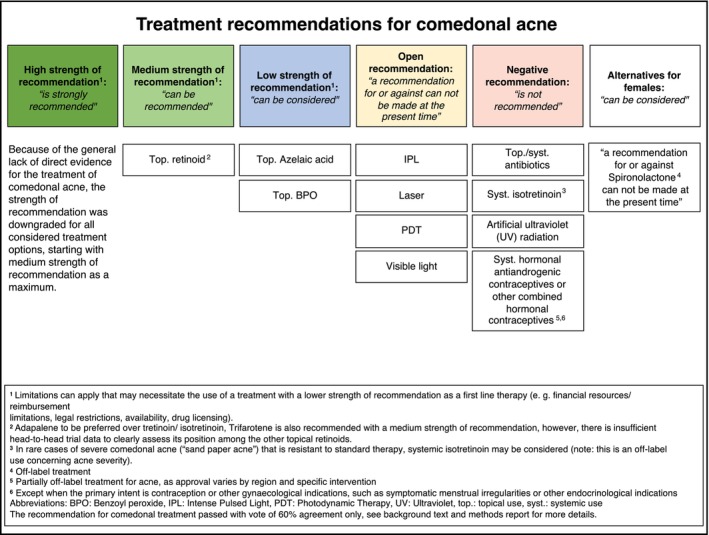
Treatment recommendations for comedonal acne.

Figure 1 presents treatment recommendations for patients with comedonal acne. A detailed rationale for these recommendations is available in the full version of the guideline (Data S1).

## TREATMENT OF PAPULOPUSTULAR ACNE

### Recommendations for mild to moderate papulopustular acne

**FIGURE 2:**
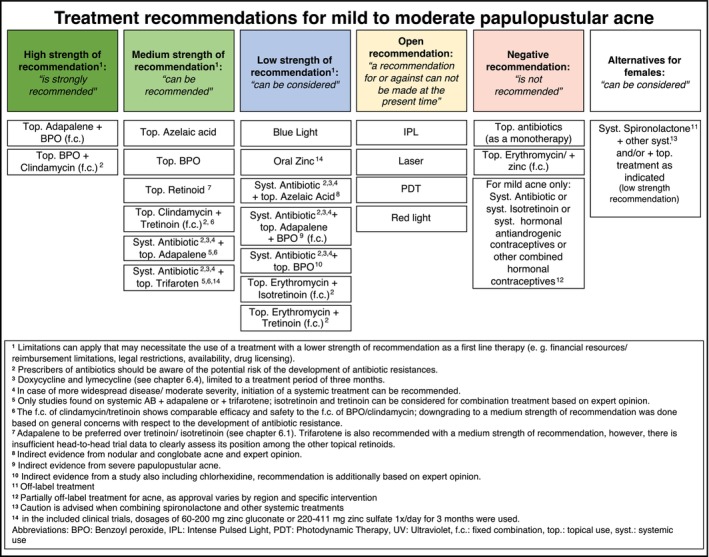
Treatment recommendations for mild to moderate papulopustular acne.

Figure 2 presents treatment recommendations for patients with mild to moderate papulopustular acne. A detailed rationale for these recommendations is available in the full version of the guideline (Data S1).

### Recommendations for severe papulopustular/moderate nodular acne

**FIGURE 3:**
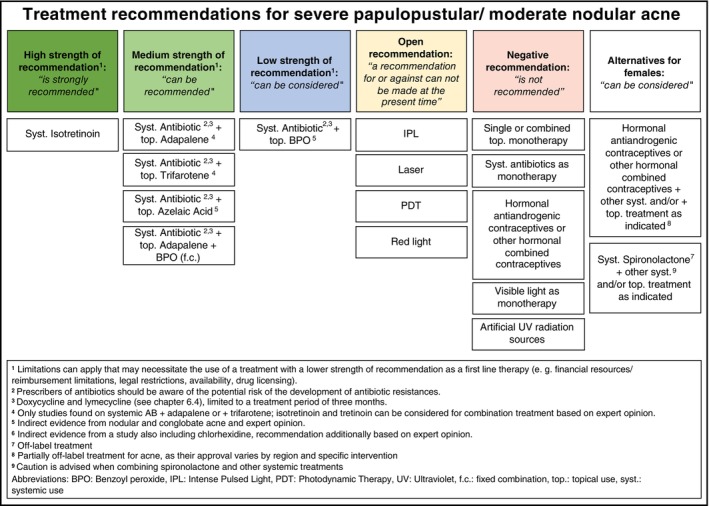
Treatment recommendations for severe papulopustular/moderate acne.

Figure 3 presents treatment recommendations for patients with severe papulopustular/moderate nodular acne. A detailed rationale for these recommendations is available in the full version of the guideline (Data S1).

## TREATMENT OF SEVERE NODULAR/CONGLOBATE ACNE

### Recommendations for severe nodular/conglobate acne

**FIGURE 4:**
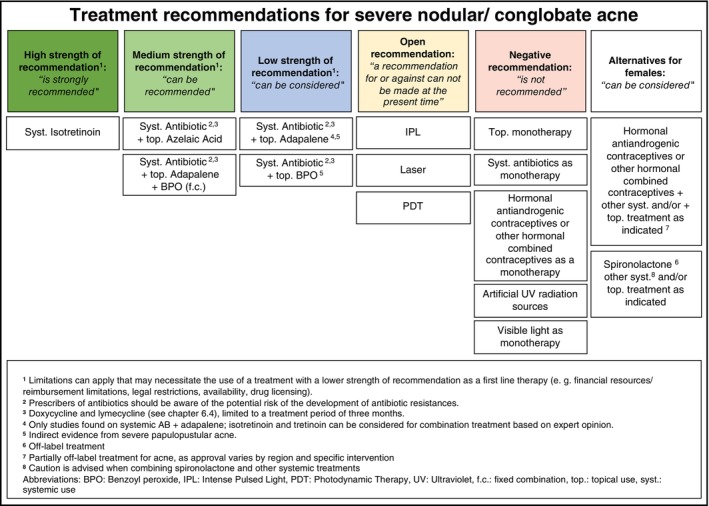
Treatment recommendations for severe nodular/conglobate acne.

Figure 4 presents treatment recommendations for patients with severe papulopustular/moderate nodular acne. A detailed rationale for these recommendations is available in the full version of the guideline (Data S1).

## GENERAL CONSIDERATIONS

### Choice of type of topical retinoid

Adapalene should be selected in preference to tretinoin and isotretinoin.

There is currently not enough evidence to compare trifarotene against adapalene, tretinoin or topical isotretinoin, as no head‐to‐head trials are available.

Studies on trifarotene focused on truncal acne, where it was shown to be effective compared to placebo. For further information and supporting evidence, see the full version of the guideline (Data S1), the methods report (Data S2) and the evidence report (Data S3).

### Considerations on the safety of benzoyl peroxide (BPO)

Concerns about the potential cancer risk, particularly leukaemia of BPO, arose from reports that some over‐the‐counter BPO products can degrade into benzene at high temperatures (37–70°C) and release it into the air from closed packages. Benzene is a known carcinogen linked to leukaemia, particularly acute myeloid leukaemia (AML).

However, current evidence does not show an increased cancer risk in acne patients using BPO. Two large studies support this: one found no higher AML prevalence among BPO users, and another found no increased risk of lymphoma, leukaemia or other internal cancers compared to matched controls. For further information and supporting evidence see the full version of the guideline (Data S1) and the methods report (Data S2).

While further research is needed, existing data support the safety of BPO in acne treatment.

### Considerations regarding clascoterone

Clascoterone was part of the evidence assessment, and all extracted data can be found in the full version of the guideline (Data S1), the methods report (Data S2) and the evidence report (Data S3). For clascoterone, a marketing‐authorization application was submitted to the European Medicines Agency (EMA) in October 2023. In April 2025, the EMA's Committee for Medicinal Products for Human Use (CHMP) initially rejected the application, stating that the benefit–risk ratio in adolescents (12–17 years) was not sufficiently demonstrated. After a re‐examination, the CHMP changed its position and on 25 August 2025 adopted a positive recommendation for the approval of Winlevi for the treatment of acne vulgaris in adults and for facial acne in adolescents from 12 to <18 years. On 21 October 2025, the European Commission granted the marketing authorization in the EU with those indications.

Because it was not yet approved at the time of the consensus conference and clinical experience was lacking, the group decided not to yet integrate it into the treatment algorithm and the recommendations.

### Choice of type of systemic antibiotic

Doxycycline and lymecycline can be recommended in preference to minocycline and tetracycline.

Azithromycin has been considered as an alternative antibiotic; however, at the time of guideline development, a recommendation by the EMA committee was published advising against its use for the treatment of acne, as current evidence does not sufficiently support its efficacy, and the ‘benefits do not outweigh the risks’, particularly with regard to antimicrobial resistance.^2^ For further information and supporting evidence, see the full version of the guideline (Data S1), the methods report (Data S2) and the evidence report (Data S3).

### Recommended treatment duration with systemic antibiotics

Table 3 presents recommendations for the treatment duration of systemic antibiotics. It is emphasized that systemic antibiotic treatment beyond 3 months should remain exceptional and should be carefully weighed against the risk of resistance.

**TABLE 3:** Recommended treatment duration with systemic antibiotics.

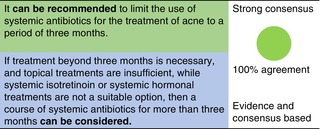

#### Reasoning

It is emphasized that systemic antibiotic treatment beyond 3 months should remain exceptional and should be carefully weighed against the risk of resistance. Most of the included trials on systemic antibiotic treatment of acne have a treatment duration of 12 weeks, with some extending up to 16 or 18 weeks.

A duration of 3 months may be too short for the treatment of severe acne or in case of truncal involvement, hence treatment continuation may be advisable to continue a treatment with partial response and continuing improvement. In addition, a prolonged treatment may be necessary in case of recurrence after previous successful treatment cycles, if other treatments are not a suitable alternative.

Indirect evidence from other indications such as chronic obstructive pulmonary disease (COPD), asthma, schizophrenia, bronchiectasis or cystic fibrosis is available, with treatment durations of more than 3 months.^3^, ^4^, ^5^, ^6^, ^7^ Examining the evidence table with the long‐term safety results in these other indications, the group did not identify AEs of particular relevance that may arise, especially when systemic antibiotics are used for more than 3 months (see evidence table S5). However, the development of antibiotic resistance is a major concern that should limit the prolonged use of systemic antibiotics. Treatment of acne with longer courses of topical or systemic antibiotics may lead to the induction of antibiotic resistance. This may contribute to increased healthcare burden and the development of antibiotic‐resistant bacteria, which is an ongoing public health concern. For further information, see section: Risk of antibiotic resistance.

### Treatment during pregnancy

This chapter is based on expert opinion/existing narrative reviews^8^ and national databases on drug safety during pregnancy. Table 4 presents recommendations for treatment during pregnancy.

**TABLE 4:** Treatment during pregnancy.

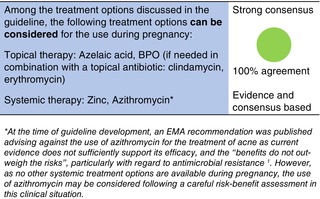

Erythromycin has not been included as a recommended treatment option in the guideline for the general acne patient population due to the high rates of antibiotic resistance; however, it can be considered as an additional option specifically for pregnant women, where other choices are limited.

Systemic corticosteroids can be considered in cases of conglobate acne with very strong inflammation, high pain levels, systemic symptoms or fulminant progression. For possible harm, see respective assessments.

There is a strong contraindication for systemic isotretinoin during pregnancy and in women trying to conceive a child due to a high teratogenic risk. Effective contraception is mandatory.^9^, ^10^

### Considerations on isotretinoin and dosage

Table 5 presents recommendations for isotretinoin dosage.

**TABLE 5:** Considerations on isotretinoin and dosage.

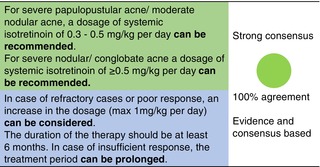

An evidence‐based recommendation regarding a cumulative dose required to prevent relapse or the need for an isotretinoin retrial cannot be provided at this time.

Based on clinical experience, most members of the guideline group treat with isotretinoin for at least 6 months, whereas the patient should be clear of inflammatory lesions for at least 1–2 months, depending on the severity.

As differences in pharmacokinetics between different brands of isotretinoin cannot be ruled out, it is advisable to prescribe a certain product and to use this same preparation throughout a treatment period.^11^

In clinical practice, an initial ‘flare up’ of acne has been observed after the initiation of systemic isotretinoin.

In cases of conglobate acne with very strong inflammation, high pain level, systemic symptoms or fulminant progression, the combination of isotretinoin and systemic glucocorticosteroids can be considered to gain clinical control to reduce strong inflammation. Suggestions on glucocorticosteroid dosing (0.5–1 mg/kg prednisone daily for 4–5 weeks) have been published as expert opinion–based recommendations by Greywal et al.^12^ For further information and supporting evidence, see the full version of the guideline (Data S1), the methods report (Data S2) and the evidence report (Data S3).

### Isotretinoin considerations with respect to EMA directive and selection of systemic antibiotics versus systemic isotretinoin

### Isotretinoin and risk of abnormal wound healing

### Consideration on isotretinoin and the risk of depression

For these chapters, please see the full version of the guideline (Data S1).

### Risk of antibiotic resistance

As outlined in the chapter ‘Recommended treatment duration with systemic antibiotics’, systemic antibiotics should generally be limited to a maximum of 3 months, with longer use considered only in exceptional cases when alternatives are unsuitable. Treatment of acne with longer courses of topical or systemic antibiotics may increase the likelihood of inducing antibiotic resistance. Such resistance contributes to increased mortality and extended hospitalizations attributable to antibiotic‐resistant pathogens, representing a critical public health challenge worldwide, including across Europe. It is well known that one broad‐spectrum antibiotic can select for multi‐resistance against a number of different antibiotics.^13^ Furthermore, it has been shown that even low concentrations of antibiotics, well below the MIC value, may select for even high‐level resistance.^14^, ^15^ The use of antibiotics to treat acne may lead to resistance in local *Cutibacterium acnes* and other local cutaneous bacteria including staphylococci, and importantly, also in species of the patients' total microbiome on skin and mucosal surfaces. Resistance may spread from non‐pathogenic/commensal to pathogenic species.

For detailed information on the risk of antibiotic resistance and the full version of the updated chapter, please see the full version of the guideline (Data S1).

### Use of hormonal antiandrogenic contraceptives or other combined hormonal contraceptives

For treatment recommendations with respect to specific acne subtypes, see chapter 2 ‘Induction therapy’. Combined oral hormonal contraceptives are widely used for pregnancy prevention and other health benefits, such as acne treatment. These contraceptives contain a combination of oestrogen and progestin, which work together to inhibit ovulation and regulate menstrual cycles. The oestrogen component is usually ethinyl estradiol (EE), a synthetic form of oestrogen, though some newer formulations use alternatives such as 17β‐estradiol, which closely resembles natural human oestrogen, as well as oestradiol valerate or estetrol. Progestins, synthetic forms of progesterone, are designed to enhance contraceptive efficacy by preventing excessive endometrial growth and suppressing ovulation and are categorized by generations.^16^, ^17^

Early‐generation progestins (first, second and third) are derived from testosterone and can have androgenic effects when used alone and therefore may worsen acne. Newer generations of progestins have been developed to minimize androgenic effects and may offer additional benefits like antiandrogenic and anti‐mineralocorticoid properties. Therefore, these newer progestins lead to fewer issues like acne and excessive hair growth (hirsutism), but may carry increased risks of venous thromboembolism (VTE).^16^, ^17^

Several COCs are licensed for the treatment of acne and patients should be counselled on their risks and benefits relevant to the primary treatment objective, individualized risk factors and local regulatory practice. The use of COCs to treat acne outside their licensed indications could carry additional risks and patients should be counselled on potential side effects and long‐term health impact as per the Summary of Product Characteristics (SmPC) and national regulatory guidance.

Hormonal antiandrogenic contraceptives or combined oral contraceptives (COCs) can be considered as adjunctive therapy in female patients with moderate to severe papulopustular, nodular or conglobate acne, particularly when contraception is desired or when acne is associated with hormonal influences or androgen excess. Systemic therapy with hormonal antiandrogenic contraceptives or other combined hormonal contraceptives (see Table 6) is not recommended for the treatment of comedonal or mild to moderate papulopustular acne, except when the primary intent is contraception or the management of concurrent gynaecological conditions, such as menstrual irregularities or other endocrinological conditions.

**TABLE 6:** Different contraceptives and their possible impact on acne.

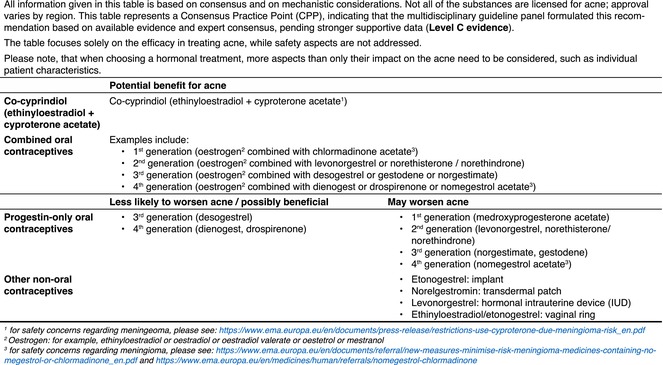

Table 6 gives an overview of the different generations of contraceptives and their possible impact on acne.

Table 6 is based on consensus and on mechanistic considerations only. The table focuses solely on the efficacy in treating acne, while safety aspects are not addressed. Please note that when choosing a hormonal treatment, more aspects than only their impact on the acne need to be considered, such as safety, contraindications and individual patient characteristics. Please note that the use of hormonal treatments for acne is partly off‐label, as approvals vary by region and product; prescribing COCs outside their licensed indications may carry additional risks, and patients should be counselled on potential side effects and long‐term health impacts according to the Summary of product characteristics (SmPC) and national regulatory guidance. While some hormonal antiandrogenic or combined hormonal contraceptives are primarily used for contraception, their anti‐acne efficacy differs by formulation and is mainly associated with progestins exhibiting antiandrogenic activity.

For detailed information on the efficacy and safety of hormonal antiandrogenic contraceptives or other combined hormonal contraceptives, please see the full version of the guideline (Data S1), the methods report (Data S2) and the evidence report (Data S3).

### Use of spironolactone

Due to its antiandrogenic properties, spironolactone is used off‐label to treat acne in women. This treatment is particularly beneficial for women with acne linked to hormonal imbalances, such as those associated with polycystic ovary syndrome (PCOS). As new evidence on spironolactone's efficacy has been published and as the guideline group perceived a need for additional systemic treatment options, it decided to include spironolactone into the guideline despite the fact that it is not licensed for acne in Europe.

For treatment recommendations with respect to specific acne subtype, see chapter 2 ‘Induction therapy’. For detailed information on the efficacy and safety of spironolactone, please see the full version of the guideline (Data S1), the methods report (Data S2) and the evidence report (Data S3).

#### Dosing recommendation and treatment duration

Start with 50 mg, increasing to 100 mg after 4 weeks if well tolerated (e.g. no breast tenderness, fatigue, headache and dizziness/hypotension).

Patients should be informed about the gradual onset of effectiveness; continued improvement has been observed in clinical trials over 6 months' duration.

#### Monitoring

No laboratory monitoring is needed for healthy patients under 45; individual factors may warrant exceptions.^16^

## AUTHOR CONTRIBUTIONS

A. Nast Conceptualization, supervision, visualization, writing—original draft, writing—review and editing. B. Al Wattar Conceptualization, visualization, writing—original draft, writing—review and editing. M. Beylot Barry Conceptualization, visualization, writing—original draft, writing—review and editing. H. Brüggemann Conceptualization, visualization, writing—original draft, writing—review and editing. Z. Bukvić Mokos Conceptualization, visualization, writing—original draft, writing—review and editing. D. M. Caruana Conceptualization, visualization, writing—original draft, writing—review and editing. K. Degitz Conceptualization, visualization, writing—original draft, writing—review and editing. C. Dessinioti Conceptualization, visualization, writing—original draft, writing—review and editing. B. Dréno Conceptualization, visualization, writing—original draft, writing—review and editing. R. J. B. Driessen Conceptualization, visualization, writing—original draft, writing—review and editing. H. Gollnick Conceptualization, visualization, writing—original draft, writing—review and editing. M. Hædersdal Conceptualization, visualization, writing—original draft, writing—review and editing. A. Katoulis Conceptualization, visualization, writing—original draft, writing—review and editing. S. Läuchli Conceptualization, visualization, writing—original draft, writing—review and editing. J. Lambert Conceptualization, visualization, writing—original draft, writing—review and editing. A. M. Layton Conceptualization, visualization, writing—original draft, writing—review and editing. G. Micali Conceptualization, visualization, writing—original draft, writing—review and editing. F. Ochsendorf Conceptualization, visualization, writing—original draft, writing—review and editing. T. Sathyapalan Conceptualization, visualization, writing—original draft, writing—review and editing. K. Z. Stangeland Conceptualization, visualization, writing—original draft, writing—review and editing. S. F. Thomsen Conceptualization, visualization, writing—original draft, writing—review and editing. D. Töröcsik Conceptualization, visualization, writing—original draft, writing—review and editing. A. Pennitz Conceptualization, project administration, visualization, writing—original draft, writing—review and editing.

## FUNDING INFORMATION

This update of the EuroGuiDerm guideline was funded through the EuroGuiDerm Centre for Guideline Development. The European Dermatology Forum is responsible for fundraising and holds all raised funds in one account. The EuroGuiDerm Team is not involved in fundraising or in the decision making on which guideline (GL) or consensus statement (CS) development is funded. The decisions on which GL/CS is funded are made by the EuroGuiDerm Board of Directors independently. The European Dermatology Forum (EDF) or any other body supporting the EuroGuiDerm is never involved in the guideline development and has no say on the content or focus of the guideline.

## CONFLICT OF INTEREST STATEMENT

This is a summary of the update of the EuroGuiDerm Guideline for the treatment of acne. For the full version of the guideline, the methods report (including COI disclosures) and the evidence report, see online Data S1, S2 and S3 or https://www.guidelines.edf.one/.

## ETHICAL APPROVAL

Not applicable to this article as no experiments were carried out.

## ETHICS STATEMENT

Not applicable to this article, as no patient photos, graphics or similar were used.

## DISCLAIMER

The EuroGuiDerm Guideline for the treatment of acne was developed in accordance with the EuroGuiDerm Methods Manual v1.3, which can be found on the website of the European Dermatology Forum (EDF), subsection Guidelines: https://www.guidelines.edf.one. This work is licensed under the Creative Commons Attribution‐NonCommercial‐4.0. For further information on copyright in case of translation, adaptation, commercial use, etc., see EDF website. Copyright © European Dermatology Forum.

## Supporting information


Data S1.

Data S2.

Data S3.

## Data Availability

The dataset generated and analysed during the current study is available online (see https://www.guidelines.edf.one/edf‐guidelines‐and‐consensus‐statements).
