# Supplementary material for: Update of the EuroGuiDerm evidence‐based guideline for the treatment of acne—Short version
Source: J Eur Acad Dermatol Venereol. 2026 Mar 18;40(7):1162–72. doi: 10.1111/jdv.70331 (PMC13308670; doi:10.1111/jdv.70331)
Supplement: Supplementary file 2 — Data S2. [file JDV-40-1162-s003.pdf]

Methods Report on the Development of the European Evidence-based (S3) Guideline for the Treatment of Acne – Update 2025

Antonia Pennitz[1], Isabell Vader[1], Alexander Nast[1]

[1] Division of Evidence-Based Medicine, Klinik für Dermatologie, Charité - Universitätsmedizin Berlin, Berlin, Germany

Corresponding author:

Prof Dr. med. Alexander Nast
Division of Evidence Based Medicine (dEBM)
Klinik für Dermatologie
Charité – Universitätsmedizin Berlin
Chariteplatz 1
10117 Berlin
Germany

Tel.: +49 30 450518 313
Fax: +49 30 450518 977
E-mail: acne@debm.de

Keywords

acne vulgaris, practice guideline, evidence-based medicine, methods, topical administration, oral administration

Table of contents

List of abbreviations V

1 Introduction 1

2 Participation of relevant interest groups 1

2.1 Nomination of experts 1

2.2 Expert methods group 2

2.3 Participation of patient representatives (update 2016) 2

3 Kick-off Meeting 2

4 Working group meetings 3

5 Methods (update 2025) 3

5.1 Literature search (update 2025) 3

5.2 Standardized inclusion/exclusion criteria (update 2025) 4

5.3 Data Screening and extraction (update 2025) 8

5.4 Methodological evaluation (update 2025) 8

5.5 Results (update 2025) 9

5.5.1 Systematic search for systematic reviews for acne 9

5.5.2 Systematic search for systematic reviews for long-term safety of antibiotics 10

5.5.3 Systematic search for the new topical treatments trifarotene and clascoterone 11

5.5.4 Systematic search for primary studies for acne following the search period of the AAD guidelines 12

6 Methods (update 2016) 13

6.1 Literature search (update 2016) 13

6.2 Standardized inclusion/exclusion criteria (update 2016) 13

6.3 Data Screening and extraction (update 2016) 14

6.4 Methodological evaluation (update 2016) 15

6.5 Results (update 2016) 15

7 Reviews (update 2016) 16

7.1 Laser and light treatment ... 17

7.2 Combined oral contraceptives pills ... 17

7.3 Patient preferences ... 17

7.4 Maintenance treatment ... 17

8 Evidence tables (update 2025) ... 18

9 Evidence tables (update 2016) ... 18

9.1 Categorising available evidence (update 2016) ... 18

9.2 Presentation of data in evidence tables (update 2016) ... 19

9.2.1 Individual summary of efficacy, safety and grade of evidence ... 19

9.2.2 Overall summary of efficacy, safety and level of evidence ... 22

9.2.3 Summary tables of the EU Acne Guideline 2016 ... 24

10 Development of recommendations/ consensus process ... 24

10.1 Relating severity and type of acne to clinically relevant patient groups / summary of recommendations (update 2016) ... 24

10.2 Consensus conference (update 2016 and 2025) ... 25

10.3 Strength of recommendation (update 2016 and 2025) ... 25

10.4 Results of consensus conference (update 2025) ... 26

10.5 Results of consensus conference (update 2016) ... 26

11 Limitations ... 28

11.1 Evidence (update 2025): ... 28

11.2 Evidence (update 2016): ... 29

11.3 Cost considerations (update 2016) ... 29

12 External review (update 2025) ... 29

13 External review (update 2016) ... 29

14 Dissemination and implementation (update 2025) ... 30

15 Dissemination and implementation (update 2016) ... 30

16 Evaluation (Update 2025) ... 30

17 Evaluation (Update 2016) ... 30

18 Funding and editorial independence (update 2025) ... 31

19 Funding and editorial independence (update 2016) ... 31

20 Future updates of the Guideline (update 2025) ... 31

21 Future updates of the Guideline (update 2016): ... 31

22 Declaration and management of conflicts of interest (update 2025) .. 32

23 Declaration and management of conflicts of interest (update 2016) .. 32

24 References ... 34

25 Appendices ... 35

25.1 Appendix A Members of the EU Guideline Group (update 2025) ... 35

25.2 Appendix B Members of the EU Guideline Group (update 2016) ... 36

25.3 Appendix C Search strategies (update 2025) ... 36

25.4 Appendix D Search strategies (update 2016) ... 42

25.5 Appendix E Methodology checklist – Barbaric et al. (Preview, date: 16. May 2015) (update 2016) ... 45

25.6 Appendix F Methodology checklist – Arowojolu et al. (2012) (update 2016) ... 46

25.7 Appendix G Conflicts of interests of the members of the experts and methods group (update 2025) ... 48

25.8 Appendix H Conflicts of interests of the members of the experts and methods group (update 2016) ... 50

List of abbreviations

ADR	adverse drug reaction

1 Introduction

This methods report is an update of the 2016 edition (1).Sections that have been added or modified are highlighted in blue text.

A comprehensive description of the updating process of the European evidence-based S3 Guideline for the Treatment of Acne 2025 (hereafter referred to as EU Acne Guideline update 2025 or the Guideline or update 2025) is provided. The Guideline was developed in accordance with the standard operating procedures of the European Dermatology Forum (EDF) (for further information, please see the EuroGuiDerm manual: https://www.guidelines.edf.one/uploads/attachments/cl27xp0q300d0l2jn87ub40jo-euroguide-manual-v1-3-20-03-03-for-website.pdf). The underlying methodology incorporated the quality criteria of the Appraisal of Guidelines Research & Evaluation II (AGREE II) Instrument (2-4), as well as the recommendations of the Cochrane Collaboration and the German Association of Scientific Medical Societies (AWMF) (5).

The update of the EU Acne Guideline is based on systematic literature searches, systematic evaluations of the search results, and a consensus conference based on formal consensus methodology (nominal group technique).

2 Participation of relevant interest groups

2.1 Nomination of experts

In November 2022, the supporting societies of the EDF – EuroGuiDerm Guidelines Center were contacted to suggest one member to join the guideline development group for this update of the Guideline.

For this update, both experts in microbiology and gynaecology were included. These were suggested by members of the guideline development group.

Nominations were confirmed by the EDF. To qualify as an expert, an individual had to satisfy at least some of the following criteria:

- extensive clinical experience in the treatment of acne,
- relevant publications in the field of acne,
- relevant experience in evidence-based medicine.

Emphasis was placed on selecting a representative panel of experts from across Europe who are still actively involved in patient treatment.

2.2 Expert methods group

The Division of Evidence Based Medicine (dEBM) at Charité – Universitätsmedizin Berlin was chosen as the methodological centre due to the experience and expertise in the development of guidelines in dermatology. The first European S3 Guideline for the Treatment of Acne in 2011 and the update in 2016 were also developed by the dEBM team.

2.3 Participation of patient representatives (update 2016)

Although extensive efforts were made to find patient representatives, these were unsuccessful due to the current lack of patient organisations in this area. Patients were, however, invited to join the external review.

For a detailed overview of all participating experts, see Appendix A Members of the EU Guideline Group (update 2025).

3 Kick-off Meeting

One online conference took place on 23rd August 2023 as kick-off meeting. In the meeting, the management of conflict of interests, the copyright agreement for the European Guideline and possibility of adaptations on a national level were discussed.

A discussion of the methodological approach for the 2025 update of the guideline took place. Consensus was reached to prioritize three key questions and to address these both evidence- and consensus-based and to establish that the previous guideline remains valid.

During the kick-off meeting, the expert group discussed and confirmed the interventions and the key topics to be considered and then reached a consensus on the three key topics of the Guideline.

As a result of the kick-off meeting, the following three key topics were selected:

1) Isotretinoin versus antibiotics

2) Hormonal treatments/ spironolactone
3) New topicals

It was decided to form three working groups, to organise topic-specific discussions and to specify the key questions and the PICO-schemes in the working groups.

4 Working group meetings

In January and February 2024, three working group meetings, one for each key topic, took place.

As a result of these meetings, the following three key questions and PICO schemes were specified:

1) For patients with mild to severe acne, which therapeutic option, isotretinoin or oral antibiotics, should be preferred in terms of effectiveness, quality of life, and (long-term) safety?

2) What is the effectiveness, quality of life and (long-term) safety of hormonal treatments for (*female*) patients with acne?

3) For patients with acne, what are the effectiveness, quality of life, and (long-term) safety of "new" topical treatments?

5 Methods (update 2025)

The methods of this evidence and consensus-based guideline follow a systematic review approach including systematic literature searches, a two-step screening approach using pre-defined exclusion/inclusion criteria as well as a risk of bias assessment.

For this guideline update, the literature search is based on systematic reviews. A search for RCTs is conducted for topics that are not sufficiently answered in existing systematic reviews.

5.1 Literature search (update 2025)

MEDLINE and EMBASE (via Ovid) were systematically searched for systematic reviews on acne and on long-term safety signals of the included antibiotics.

Since no systematic reviews fulfilled the inclusion criteria for the key question 3, a systematic search for primary studies on trifarotene and clascoterone in MEDLINE and Embase (via Ovid) was conducted.

We systematically searched MEDLINE via Ovid and the Cochrane CENTRAL trials database for RCTs on acne after the last search date (11/2022) of the most recent systematic review from Reynolds et al. (6) All search strategies are shown in Appendix C Search strategies (update 2025) and Appendix D Search strategies (update 2016).

5.2 Standardized inclusion/exclusion criteria (update 2025)

The inclusion and exclusion criteria for each key question are shown in Table 1, Table 2 and Table 3, respectively.

Table 1: PICO-scheme: Key question 1: Isotretinoin vs. antibiotics

	Inclusion	Exclusion
**P - Population**	Patients, aged 12 years or older, with mild, moderate or severe acne vulgaris, acne comedonica, acne papulopustulosa, acne conglobata, acne nodularis<br>• In case of missing reporting of severity, studies will be looked at separately and sensitivity analysis will be performed<br>Any population / disease for long term safety	Patients with chloracne, acne venenata, acne fulminans, acne necroticans, acne agminata, or rosacea, occupational acne
**I - Intervention**	Systemic Antibiotics with or without topical treatment:<br>• Erythromycin<br>• Tetracycline<br>• Doxycycline<br>• Minocycline<br>• Lymecycline<br>• Azithromycin<br>• Sarecycline<br>Isotretinoin (any dosing regimen) with or without topical treatment (goal: compare different dosing regimens)<br>Isotretinoin and other Antibiotics	Topical Antibiotics<br>Other systemic antibiotics<br>Physical treatments<br>Hormonal contraceptives and hormone-modifying treatments<br>Clindamycin: only of historical interest, is no longer used
**C - Comparison**	Stepwise approach:<br>1. Any of the included interventions (head to head)<br>2. Placebo (only if reported in systematic reviews)<br>For long-term safety data also:<br>• No treatment	

	Inclusion	Exclusion
**O - Outcomes**	**Step-wise approach:**<br>1. For head to head studies, include all outcomes specified below<br>2. For placebo-controlled studies, check which are reported in systematic reviews<br>Efficacy<br>• Clinician-rated improvement:<br>○ % change in acne lesion count from baseline (TL, IL, NIL)<br>○ change or final score on a validated acne severity scale<br>• Patient-reported improvement<br>○ Change in acne severity or symptoms (e.g. assessed using global acne score)<br>○ Participant's self-assessment of acne severity<br>• Time until onset of action<br>• Relapse<br>• Quality of Life (QoL)<br>Safety<br>• Resistance data<br>• Patients with AEs of special interest, e.g. depression, IBD, sexual side effects (see Drug Safety Update volume 16, issue 9: April 2023: 1 (7)),<br>• Patientswith SAEs<br>• Dropouts due to AEs	
**S – Study types**	Stepwise approach:<br>• Systematic reviews<br>• RCTs<br>• Long-term studies only on safety data	

Table 2: PICO-scheme: Key question 2: Hormonal treatments

	Inclusion	Exclusion
**P - Population**	• Female patients with acne of any severity, acne comedonica, acne papulopustulosa, acne conglobata, acne nodularis for hormonal treatments (incl. spironolactone)<br>• Male patients with acne of any severity for spironolactone<br>• Step-wise approach:<br>1) Studies with clear reporting of normal hormonal status<br>2) If not enough data are available, also look at trials with less clear reporting or mixed population<br>3) Subchapter focussing on comorbidities	• Patients with chloracne, acne venenata, acne fulminans, acne necroticans, acne agminata, or rosacea, occupational acne<br>• Male patients with acne for hormonal treatment<br>• Patients with PCOS or hirsutism<br>• Exclude if patient population is unclear with regard to hormonal status
**I - Intervention**	• Oral hormonal contraceptives with or without topical treatment:<br>○ Co-cyprindiol (ethinylestradiol + cyproterone acetate)<br>○ Class of combined oral contraceptives	• Other hormonal contraceptives and hormone-modifying treatments:<br>○ Class of progestogen-only oral contraceptives

	Inclusion	Exclusion
	▪ Sub-class of 2nd generation (oestrogen, for example ethinylestradiol or estradiol or mestranol combined with levonorgestrel or norethisterone)<br>▪ Sub-class of 3rd generation (oestrogen, for example ethinylestradiol combined with desogestrel or gestodene or norgestimate)<br>▪ Sub-class of 4th generation (oestrogen, for example ethinylestradiol or estradiol combined with dienogest or drospirenone or nomegestrol acetate)<br>○ Class of progestogen-only oral contraceptives<br>▪ Sub-class of 3rd generation: desogestrel<br>▪ Sub-class of 4th generation: Sub-class of 4th generation: dienogest, drospirenone<br>• Spironolactone with or without topical treatment	▪ Sub-class of 1st generation (for example medroxyprogesterone acetate)<br>▪ Sub-class of 2nd generation (for example: levonorgestrel, norethisterone/ norethindrone)<br>○ Sub-class of 3rd generation: norgestimate, gestodene<br>○ Sub-class of 4th generation: nomegestrol acetate<br>• Monotherapy with:<br>○ Topical treatments<br>○ Systemic antibiotics<br>○ Isotretinoin<br>○ Physical treatments
**C - Comparison**	• Any of the included interventions<br>• Topical Treatments<br>• Systemic antibiotics<br>• Physical treatments<br>• Isotretinoin<br>• Placebo<br>• No treatment	
**O - Outcomes**	Efficacy<br><br>• Clinician-rated improvement:<br>○ % change in acne lesion count from baseline (TL, IL, NIL)<br>○ Change or final score on a validated acne severity scale<br>• Patient-reported improvement:<br>○ Change in acne severity or symptoms (e.g. global acne score)<br>○ Participant's self-assessment of acne severity<br>○ Time until onset of action<br>○ Relapse<br>○ Quality of Life (QoL)<br>Safety<br><br>• Patients with AEs<br>• If possible, include AEs of special interest: If possible, patients with AEs of special interest<br>○ Spironolactone: electrolyte imbalances (hyperkalemia, hyponatraemia), hypovolemia, breast cancer<br>○ Oral hormonal contraceptives: thrombosis, breast cancer, depression, mood changes, suicidal ideation<br>• Patients with SAEs<br>• Dropouts due to AEs	
**S – Study types**	Stepwise approach:<br><br>1) Systematic reviews<br>2) RCTs	

	Inclusion	Exclusion
	3) (possibly CCTs also if 1 and 2 insufficient, possibly add narratively)	

Table 3: PICO-scheme: Key question 3: New topical treatments

	Inclusion	Exclusion
**P - Population**	Patients with acne of any severity, acne comedonica, acne papulopustulosa, acne conglobata, acne nodularis<br>• If possible, conduct subgroup analyses for severity (people with mild to moderate and moderate to severe acne)	Patients with chloracne, acne venenata, acne fulminans, acne necroticans, acne agminata, or rosacea, occupational acne
**I - Intervention**	New topical treatments licensed in Europe since publication of last update or ongoing EMA approval process with or without any systemic therapy or other topical treatment:<br>• Trifarotene<br>• Clascoterone	Monotherapy with:<br>• Other topical treatments<br>• Systemic antibiotics<br>• Isotretinoin<br>• Physical treatments<br>• Hormonal contraceptives and hormone-modifying treatments
**C - Comparison**	• Any of the included interventions<br>• Other topical treatments<br>• Any of the included interventions<br>• Oral antibiotics<br>• Isotretinoin<br>• Topical treatments<br>• Physical treatments<br>• No treatment<br>• Placebo	
**O - Outcomes**	Efficacy<br>• Clinician-rated improvement:<br>○ % change in acne lesion count from baseline (TL, IL, NIL)<br>○ Change or final score on a validated acne severity scale<br>• Patient-reported improvement:<br>○ Change in acne severity or symptoms (e.g. global acne score)<br>○ Participant's self-assessment of acne severity<br>• Time until onset of action<br>• Relapse<br>• Quality of Life (QoL)<br>Safety<br>• Patients with AEs<br>• If possible, include AEs of special interest: for clascoterone: hormonal imbalances/ adrenal suppression<br>• Patients with SAEs<br>• Dropouts due to AEs	
**S – Study types**	Stepwise approach:<br>• Systematic reviews<br>• RCTs	

5.3 Data Screening and extraction (update 2025)

All identified records were screened for inclusion/exclusion by one assessor. Each selected abstract was included in the full text screening. One assessor screened all full texts for inclusion using the pre-defined inclusion/exclusion criteria. For included studies, data was extracted by one assessor with subsequent check of accuracy by another assessor.

Any discrepancies were resolved through discussion. The final evidence tables are described in detail in Chapter 8.

5.4 Methodological evaluation (update 2025)

The AMSTAR-II tool (8) was used to assess the methodological quality of all included systematic reviews. To assess risk of bias in randomized controlled trials, the Cochrane Risk of Bias tool 2 (9) was used.

5.5 Results (update 2025)

5.5.1 Systematic search for systematic reviews for acne

The search for systematic reviews for acne generated 1866 hits. Two additional publications were identified through hand search. After de-duplication 1293 records remained. Of these, the latest 790 records were screened (from search date until 2017). 62 publications were eligible for full-text evaluation. Data was extracted from two systematic reviews for key question 1 and two for key question 2. No systematic review was included for key question 3 (see Figure 1).

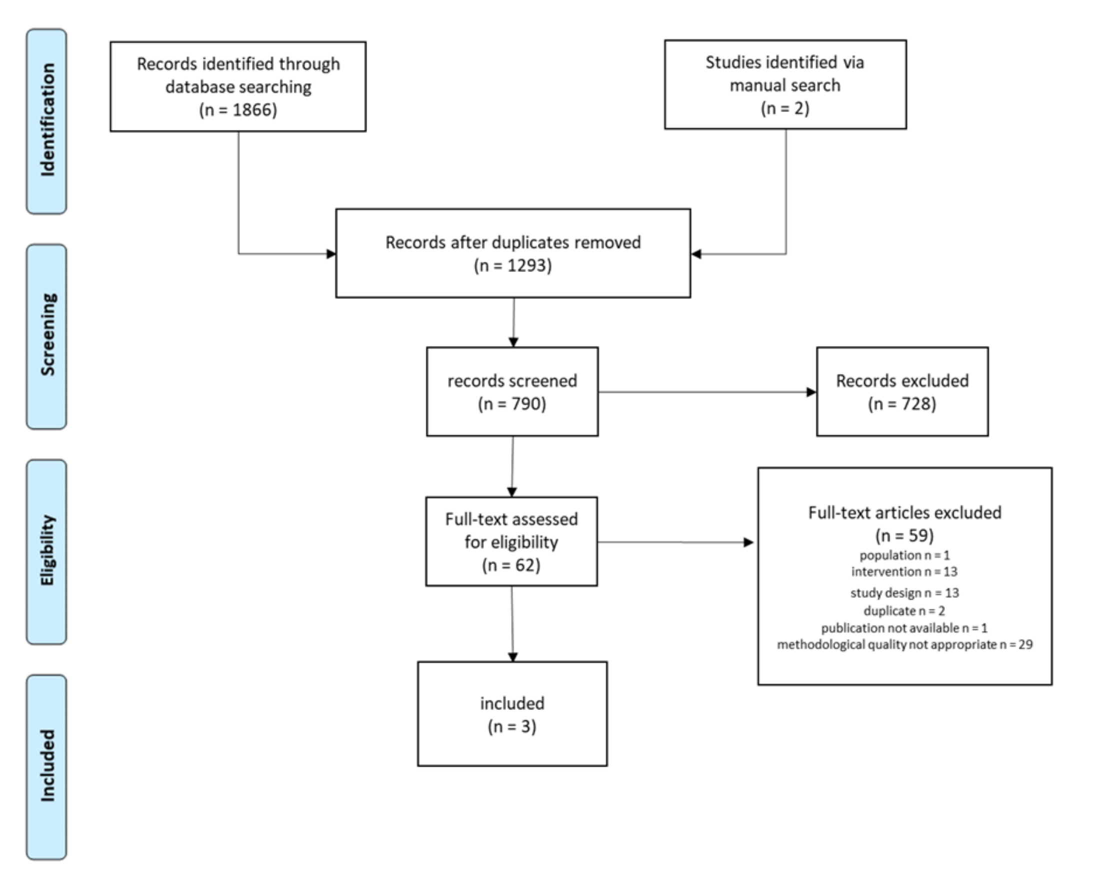

Figure 1: PRISMA-flowchart for the search of systematic reviews for acne

5.5.2 Systematic search for systematic reviews for long-term safety of antibiotics

The search for long-term safety aspects of antibiotics generated 5689 hits. After de-duplication 4589 records remained. Of these, the latest 1372 records were screened (from search date until 2021). 103 publications were eligible for full-text evaluation. Data was extracted from 12 systematic reviews for key question 1. We extracted data from three systematic reviews directly, and extracted data from 27 primary studies that were identified and reported in 9 systematic reviews (see Figure 2).

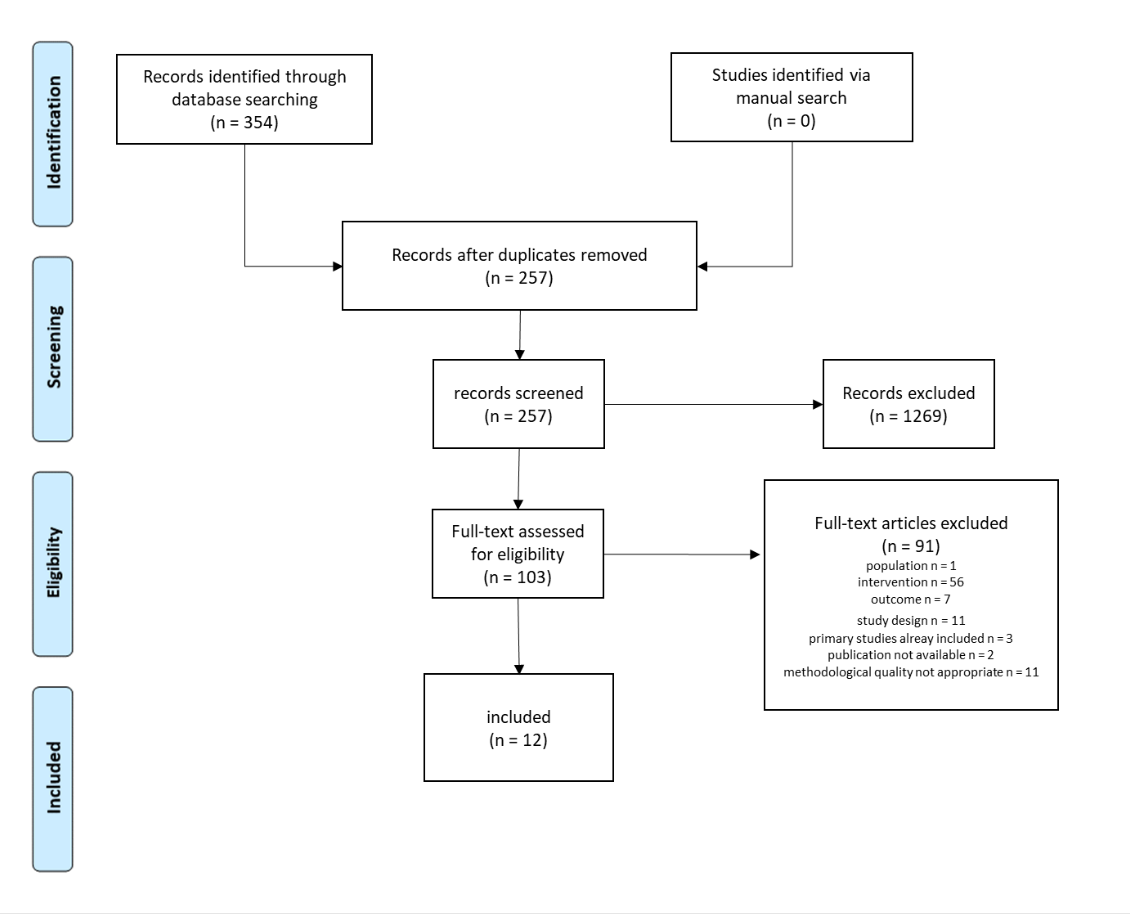

Figure 2: PRISMA-flowchart for the search of systematic reviews for long-term safety of antibiotics

5.5.3 Systematic search for the new topical treatments trifarotene and clascoterone

The search for RCTs for the new topical treatments trifarotene and clascoterone generated 354 hits. After de-duplication 257 records were screened. 56 publications were eligible for full-text evaluation. Data was extracted from 9 RCTs reported in 8 publications for key question 3. Additional safety aspects from two studies were extracted (see Figure 3).

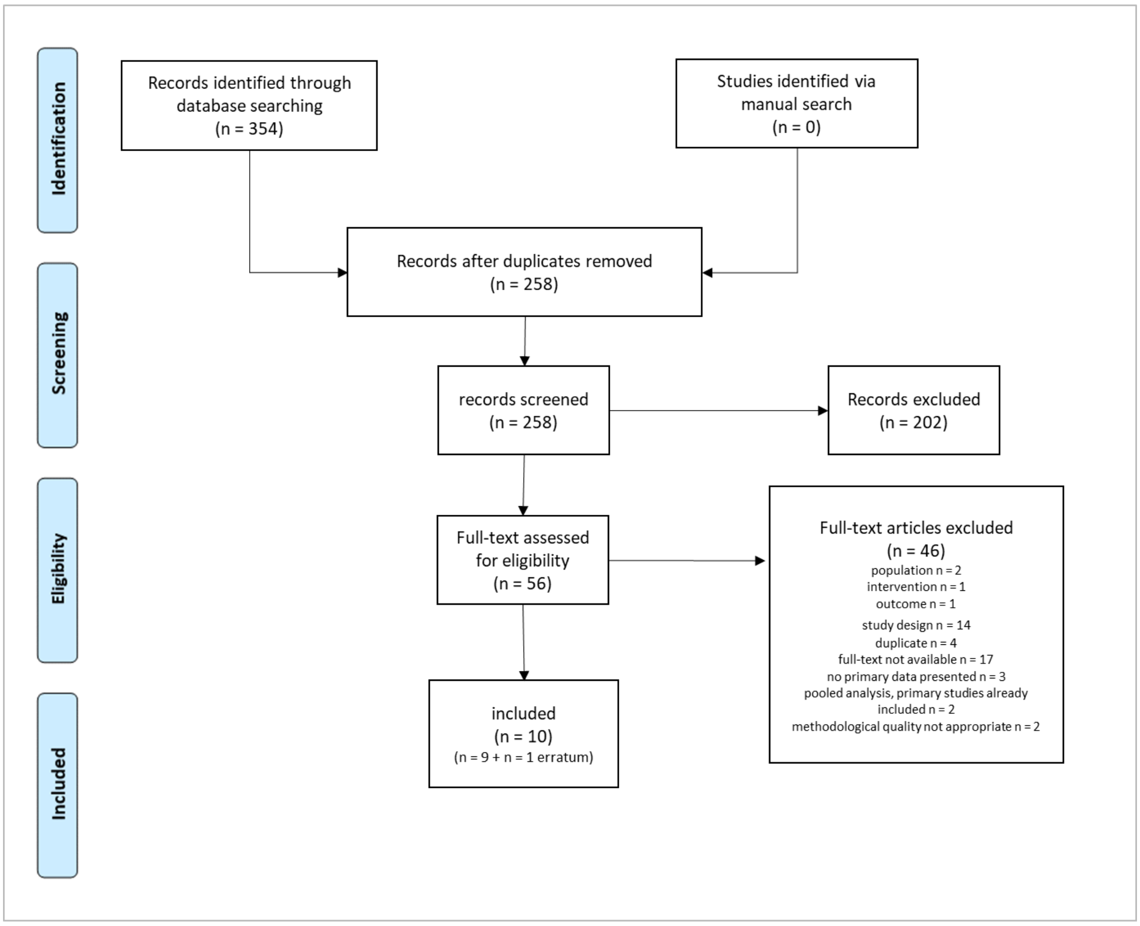

Figure 3: PRISMA-flowchart for primary studies on new topical treatments

5.5.4 Systematic search for primary studies for acne following the search period of the AAD guidelines

The search for RCTs for acne following the search period of the AAD guidelines generated 1347 hits. After de-duplication 1035 records were screened. 48 publications were eligible for full-text evaluation. Data was extracted from four RCTs for key question 1, two for key question 2 and none for key question 3 (see Figure 4).

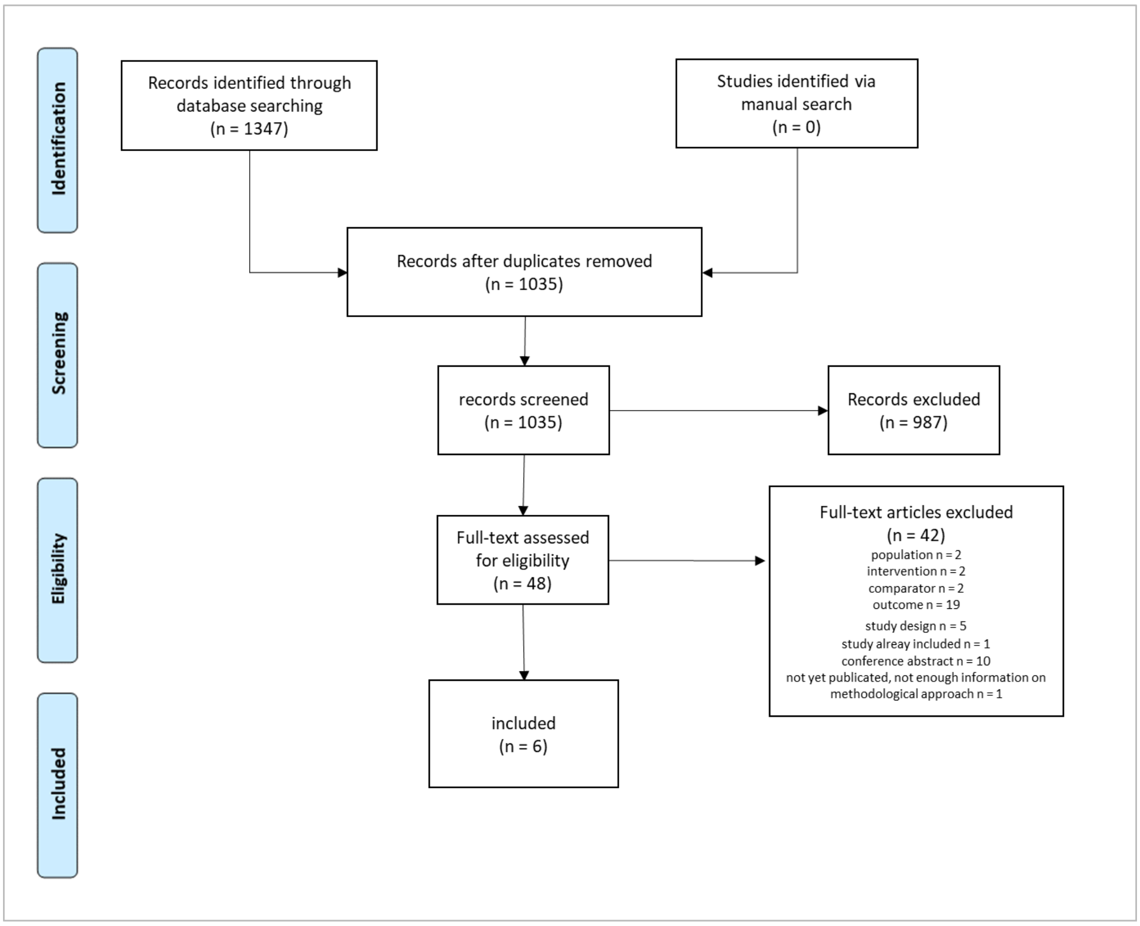

Figure 4: PRISMA-flowchart for primary studies on acne following the search period of the AAD guidelines

6 Methods (update 2016)

The methods of this evidence and consensus-based Guideline follow a systematic review approach including systematic literature searches, a two-step screening approach using pre-defined exclusion/inclusion criteria as well as a risk of bias assessment.

The nominal group process for consensus based decisions is described in Chapter 10.

6.1 Literature search (update 2016)

MEDLINE, MEDLINE In-Process and EMBASE (via OvidSP) were systematically searched (for a sample search strategy, see Appendix D). For topical and systemic treatments, the search covered 2010 to 5th July 2015. The inception dates were determined by the literature search periods covered in the previous EU Acne Guideline.

6.2 Standardized inclusion/exclusion criteria (update 2016)

The included interventions are listed in Table 1. Only randomized controlled trials evaluating the below-listed anti-acne treatments including patients with acne were eligible for inclusion. Studies had to report lesion count at baseline and follow-up, or a mean/median change in lesion count as an outcome.

Table 4: Interventions included in the guideline

Systemic treatments	Topical treatments	Light therapies
Antibiotics<br>• Erythromycin<br>• Clindamycin<br>• Tetracycline<br>• Doxycycline<br>• Minocycline<br>• Lymecycline	Antibiotics<br>• Erythromycin<br>• Clindamycin<br>• Tetracycline<br>• Nadifloxacin	Intense pulsed light
Isotretinoin	Azelaic acid	Photodynamic therapy
Combined oral contraceptives pills	Benzoyl peroxide	Blue light, red light, visible light
Zinc	Retinoids<br>• Adapalene<br>• Isotretinoin<br>• Tretinoin	Laser
	Fixed combinations:<br>Adapalene/BPO<br>BPO/clindamycin<br>Erythromycin/tretinoin<br>Erythromycin/isotretinoin<br>Erythromycin/zinc<br>Clindamycin/tretinoin<br>Clindaymcin/zinc	

Exclusion criteria (adapted from previous EU Acne Guideline 2011) were as follows:

- Study does not address management of active acne,
- more than 20% of the patients have chloracne, acne venenata, acne fulminans, acne necroticans, acne agminata, or rosacea,
- occupational acne,
- fewer than 12 patients randomized per study arm.

6.3 Data Screening and extraction (update 2016)

All identified records were screened for inclusion/exclusion by two independent assessors (SR, CD). Each selected abstract was included in the full text screening. Two assessors (SR, UA) screened all full texts for inclusion using the predefined inclusion/exclusion criteria. For included studies, data was extracted independently by two assessors (SR, UA) using a standardized data extraction form (MS Excel sheet) containing the following items:

- Author, year
- Intervention, control intervention(s)
- Number of randomized participants
- Severity of acne
- Study duration
- Percentage reduction in lesion count from baseline to time point of evaluation
- Other outcomes/statistics
- Comments
- Safety
- Number of drop outs due to adverse events

The forms were compared and any discrepancies were reviewed by a third assessor (RE) and resolved through discussion. The final evidence tables are described in detail in Chapter 8.

6.4 Methodological evaluation (update 2016)

A basic methodological evaluation took place. An assessment of study conduct in regards to blinding (evaluator, assessor, investigator and/or patient), the generation of the randomization list and in regards to the statistical analyses took place. These were the basic criteria for the assignment of the grade of evidence, see explanations for ‘Grade of evidence’ in Section 89.29.2.1.

6.5 Results (update 2016)

The update search generated 990 hits. After de-duplication 806 records were screened. The two independent assessors (SR, CD) determined that 87 publications were eligible for full-text evaluation. Two of these 87 publications had been identified through reference list screening and packaging inserts. Data was extracted from 47 articles (including one article reporting data for two studies).

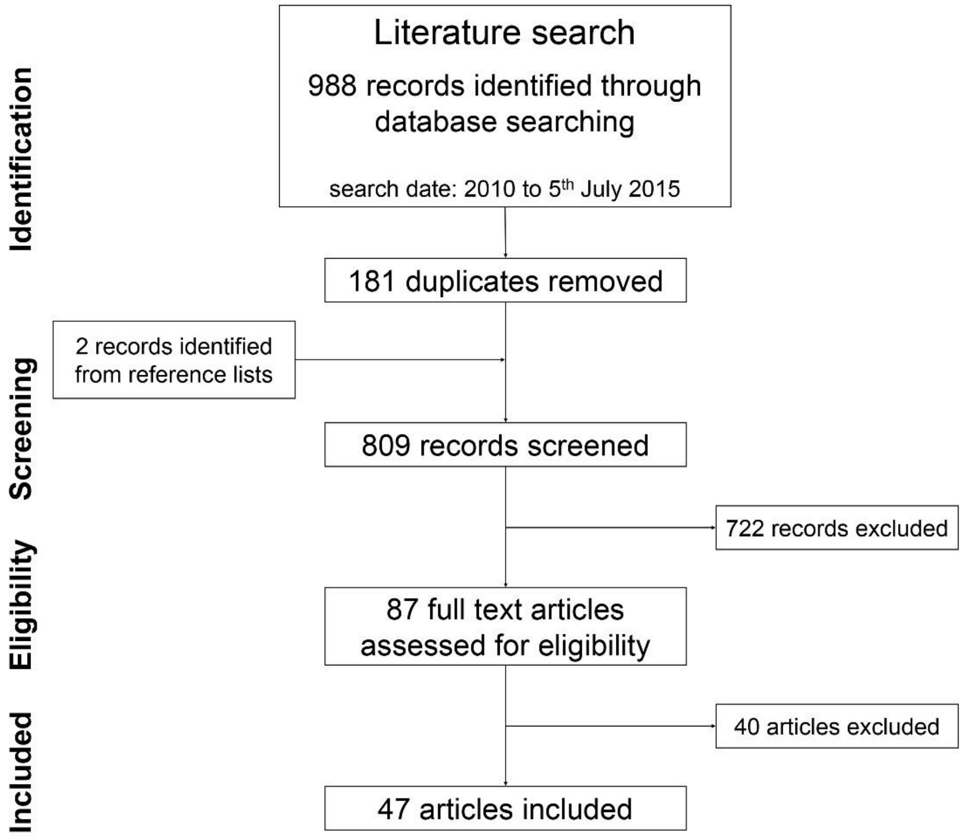

Figure 5: Flow of information – update search for induction treatment of acne

As this is an update of the EU Acne Guideline 2011, altogether, data from 210 records (222 RCTs) was extracted, see evidence tables, Chapter 8.

Included in the EU Acne Guideline 2016 - as tabular summaries - are the results of 154 studies (126 from previous guideline version, 28 from update search) as decided by the experts with regards to clinical relevance.

7 Reviews (update 2016)

During the kick-off meeting it was decided to use Cochrane reviews for the areas of ‚laser and light therapy’ and ‘hormone therapy’. Furthermore, the dEBM team conducted two systematic reviews independently, one for maintenance therapy and one for patient preferences; the results of which were also used as evidence base.

7.1 Laser and light treatment

For reasons of feasibility the group decided to use the Cochrane Review currently being developed by Barbaric et al. The authors contacted and provided the guidelines group with a preliminary draft. However, unfortunately, the final version of the Cochrane review was not finished by the time the guideline was finalised. The guidelines group did take the results of the systematic review of the 2011 version of the guideline together with the preliminary results of the Cochrane review into account to phrase the current recommendations of the update. The preliminary version of the Cochrane systematic review “Light therapies for acne” by Barbaric et al. (10) was checked for sufficient methodological quality (see Appendix E, checklist by Scottish Intercollegiate Guidelines Network [SIGN]). Detailed results from the Cochrane review could unfortunately not be displayed in the guidelines as it was still preliminary.

7.2 Combined oral contraceptives pills

The Cochrane review “Combined oral contraceptive pills for treatment of acne” by Arowojolu et al. (2012) (11) served as a base for the induction treatment with hormonal agents. Author conclusions were included in the Guideline. The reviews had been assessed and an acceptable methodological quality was determined using the “Methodology Checklist 1: Systematic Reviews and Meta-analyses” by SIGN (see Appendix F).

7.3 Patient preferences

The dEBM team conducted a separate systematic review on the available evidence of patient preferences in acne treatment. The results were published separately (12) and taken into consideration for the guideline.

7.4 Maintenance treatment

The dEBM team conducted an independent systematic review on acne maintenance treatment defined as ‘maintenance is the treatment period that follows a successful induction therapy at the end of which patients had achieved a pre-

defined treatment goal'. The results were published separately (13) and taken into consideration for the guideline.

8 Evidence tables (update 2025)

Four new evidence tables, one for each key question, for the induction treatment of acne are appended to the EU Acne Guideline 2025 as separate tables.

9 Evidence tables (update 2016)

Three evidence tables (MS Excel) for the induction treatment of acne are appended to the EU Acne Guideline 2016; the first two tables (comedonal and papulopustular acne) contain multiple sheets, separated by treatment. The last table, conglobate acne, contains only one sheet due to scarce evidence.

9.1 Categorising available evidence (update 2016)

Only RCTs reporting lesion counts were included and systematically assessed. Studies highlighted in green have been added as a result of the update literature search.

Attempts were made to match the study populations in the included trials to the different acne types as defined by the expert group (comedonal/ papulopustular/ conglobate). Attempts were also made to identify sources of indirect evidence to serve as a base for the assessment of efficacy in the given acne types. Certain studies provided evidence applicable to different acne types (e.g. inflammatory lesion counts applicable to papulopustular acne and non-inflammatory lesion counts applicable as indirect evidence for comedonal acne) and hence were included in both evidence tables.

1) Comedonal acne: A trial on comedonal acne was categorized as such if (a) this had been clearly stated by the authors and/or (b) this designation could be confirmed by patient baseline data provided (comedo count provided, few or no inflammatory lesions). Due to the paucity of studies on comedonal acne, indirect evidence was generated by means of looking at the percentage decrease in non-inflammatory lesions (NIL) in trials on patients with other acne types.

2) Papulopustular acne: A trial on papulopustular acne was categorized as such if (a) this had been clearly stated by the authors of the trial and/or if (b) this

designation could be confirmed by patient baseline data. For papulopustular acne, the EU Guideline group had agreed that inflammatory lesions (IL) count as outcome measure would provide the best evidence.

3) Nodular/conglobate acne: A trial on nodular/conglobate acne was categorized as such if (a) this was clearly stated by the authors and/or (b) the study reported a respective nodule and/or cyst count at the beginning of the trial. The percentage reduction in nodules (NO) or cysts (CY) served as the main outcome measure.

9.2 Presentation of data in evidence tables (update 2016)

On each sheet, headings and subheadings name the comparisons and then list the included studies. Underneath all of the studies within one comparison, summaries of efficacy and safety/ tolerability are provided taking all studies into account (some exceptions apply, see corresponding details below).

9.2.1 Individual summary of efficacy, safety and grade of evidence

Extracted items (Figure 6) are explained below:

Author(s)	Interventions	N	S	D	B	% ↓IL	Other Outcomes / statistics	Comments	Summary of efficacy	Safety	Drop outs	Summary of safety	Grade of evidence

Figure 6: Heading of evidence tables – example: papulopustular acne

“Author(s)”: States the name of the first author and the year of publication; in case of data from the same study group being published in separate publications, the first author of each publication is listed. The reference number of the study corresponds to the number in the reference list (provided in parentheses).

“Interventions”: Lists the investigated medications with concentration, application regime.

“N = number”: Total number of patients randomized is provided; for studies identified in the current literature search numbers were extracted for each treatment arm separately.

“S = severity”: Grade of severity (1 - mild, 2 - moderate or 3 - severe) as defined by authors of the study or if no such categorization was provided, as assessed

by the guideline group taking into consideration the lesion count at baseline or the scale/score given at baseline (for example, Burke-Cunliffe/Leeds score). See chapter 2.1.1 Acne grading system of guideline text.

"D = duration": Duration of study in weeks stated; in cases where a study lasted longer than 12 weeks, the methodologists attempted to extract outcome data for week 12 (or as close to this point as possible); in this case, two information are provided the first number gives the overall length of the study in weeks, whereas the second number gives the time point at which outcome data were extracted.

"B = Blinding": Data was extracted as provided by the authors (I - investigator-blinded; P - patient-blinded; E - evaluator-blinded; A - assessor-blinded; 1x - single-blinded; 2x - double-blinded).

"% □ NIL/IL = Percentage reduction in lesion count": Percentage reduction in lesion count from baseline to time point of evaluation.

Table 'Comedonal acne': The mean percentage reduction in non-inflammatory lesions is stated. Where this was not available, the percentage reduction in comedones (open and/or closed) is listed. In absence of the mean percentage reduction of lesion count, the median percentage reduction was extracted.

Table 'Papulopustular acne': The mean percentage reduction in inflammatory lesions is stated. Where this was not available or calculable, the median percentage reduction in IL is stated. The reporting of papules and pustules was seen as equivalent to IL. If studies reported mean percentage reduction in papules and pustules separately, we calculated the mean percentage reduction in IL (adding papules [PA] count and pustules [PU] count, divided by 2). If none of the above mentioned lesion counts were available, we reported the mean percentage reduction in total lesion count (total lesion count = NIL + IL count).

Table 'Conglobate acne': The mean percentage reduction in nodules and cysts is provided. If this was not available or calculable, the mean percentage reduction in nodules or cysts separately, or in IL was extracted.

“Other statistics”: Any other statistical information provided in the paper and considered relevant.

“Comments”: Summary of additional information about the methods such as randomization, data analyses (ITT), other relevant information (e. g. split-face, age if study on children, only abstract available, etc.).

“Summary of efficacy”: NIL, IL, NO, CY or their components (papules, pustules, open or closed comedones) were taken into account. We defined a treatment to be superior to another treatment if there is a difference ≥ 10% in efficacy outcome (see chapter 3).

“Safety”: The number of patients with at least one adverse event and the three most common adverse events as stated by the author(s).

“Drop-outs”: Number of drop-outs due to adverse events.

“Summary of safety”: A global comparison of safety data was done taking into consideration the number of patients with at least one adverse event, the three most common adverse events and drop-out rates due to adverse events. If no such data was available the authors’ conclusion was added. If studies failed to report safety/ tolerability aspects no summary of safety/ tolerability was drawn (reported as ‘insufficient data’).

Summary of safety was not done for studies comparing verum versus vehicle/placebo. Direct comparisons of active treatments are the main focus of the guideline in terms of safety.

“Grade of evidence”: Each trial included in the Guideline was evaluated with regard to its methodology and assigned a grade of evidence according to a modified grading system used in previous guidelines (14, 15).

A Randomized, double-blind clinical trial of high quality (for example, sample-size calculation, flow chart of patient inclusion, intention-to-treat [ITT] analysis, sufficient sample size)

B Randomized clinical trial of lesser quality (for example, only single-blind, no ITT)

C Comparative trial with severe methodological limitations (for example, not blinded, very small sample size)

9.2.2 Overall summary of efficacy, safety and level of evidence

For each comparison the overall evidence was assessed, a 'level of evidence' assigned and a summary for efficacy and safety/ tolerability given (Figure 7).

Summary efficacy: comparable efficacy azelaic acid = BPO (range reduction IL: azelaic acid: 45 - 84%, BPO: 44 - 84%) (LE 2)<br>Azelaic acid = BPO: 1 A study, 1 B study, 1 C study
Summary safety / tolerability: superior safety/tolerability azelaic acid > BPO (LE 4)<br>Azelaic acid > BPO: 1 A study, 1 C study

Figure 7: Summary efficacy and safety – example: papulopustular acne

Level of evidence (LE): In addition to assigning a grade of evidence to individual trials, the methodologists assigned levels of evidence to the various treatment options. The levels of evidence, which can be regarded as an overall rating of the available efficacy and safety/ tolerability data for each treatment option, were defined as follows:

1 ***Further research is very unlikely to change our confidence in the estimate of effect.***

At least two trials are available that were assigned a grade of evidence A and the results are predominantly consistent with the results of additional grade B or C studies.

2 ***Further research is likely to have an important impact on our confidence in the estimate of effect and may change the estimate.***

At least three trials are available that were assigned a grade of evidence B and the results are predominantly consistent with respect to additional grade C trials.

3 ***Further research is very likely to have an important impact on our confidence in the estimate of effect and is likely to change the estimate.***

Conflicting evidence or limited amount of trials, mostly with a grade of evidence of B or C.

4 ***Any estimate of effect is very uncertain.***

Little or no systematic empirical evidence; included trials are extremely limited in number and/ or quality.

The assignment of the different grades of evidence to a resulting level of evidence is shown in Table 2.

One trial with a grade of evidence A is equivalent with respect to its impact on the level of evidence to two trials with a grade of evidence B.

One study with a grade of evidence A is equivalent with respect to its impact on the level of evidence to four trials with an evidence grade of C.

Table 5: Generation of level of evidence

Level of evidence	Number of studies with specific grade of evidence
Summary of efficacy:	
1	At least 2 A studies
2	At least 1 A study and 1 B study
3	At least 1 A study
4	Less than 1 A study
Summary of safety/ tolerability:	
1	At least 3 A studies
2	At least 2 A studies and 1 B study
3	At least 2 A studies
4	Less than 2 A studies

Summary efficacy: Within each comparison, each study was assigned a grade of evidence. If the studies came to the same conclusion, they were grouped together/added, and then compared to studies/grouped studies with other conclusions. Hereby the grades of evidence were added/subtracted, as appropriate, the result was than transformed to a final level of evidence (see Table 2).

Summary safety/ tolerability: Results of studies within a comparison were grouped in the same manner as was done for summary of efficacy. Grades of evidence of the studies were transformed into a level of evidence for the safety/ tolerability conclusion.

Safety and tolerability criteria to achieve a higher level of evidence were stricter than for the efficacy assessment (see Table 2).

9.2.3 Summary tables of the EU Acne Guideline 2016

Due to the large amount of available treatment options with a multitude of possible comparisons, only selected comparisons on efficacy and safety/ tolerability were transferred into the summary tables.

Selection of presented comparisons:

Monotherapy with topical antibiotics was not considered due to the risk of the development of antibiotic resistance. Different dosages and frequencies of application were pooled whenever possible. For combination treatments, only marketed fixed combinations were included.

For comedonal acne, only topical treatments were selected as appropriate.

There is only one study investigating patients with comedonal acne therefore no summary of direct evidence for safety/ tolerability could be included. Summary of efficacy for comedonal acne is based on indirect evidence from NIL counts in trials on papulopustular acne.

10 Development of recommendations/ consensus process

10.1 Relating severity and type of acne to clinically relevant patient groups / summary of recommendations (update 2016)

To reflect frequent clinical situations for which guidance is needed, a fourth group summarizing severe papulopustular acne/moderate nodular acne was introduced. With this, four relevant clinical patient populations where defined: a) comedonal acne; b) mild to moderate papulopustular acne; c) severe papulopustular acne/moderate nodular acne, and d) conglobate acne.

Since evidence for severe papulopustular acne could not be directly extracted from clinical trials, attempts were made to differentiate studies on mild to moderate papulopustular acne versus moderate to severe papulopustular acne.

10.2 Consensus conference (update 2016 and 2025)

All recommendations were discussed and voted on via an online consensus conference using formal consensus methodology. The conferences were chaired by Professor Dr. med. Alexander Nast.

First, the existing evidence was presented to the group and discussed with regard to efficacy, safety, patient preference and other relevant factors, for example, antibiotic resistance or pathophysiological reasoning.

For each recommendation, the number of supporting experts was documented. Three levels of consensus were defined and distinguished: ‘strong consensus’ (agreement of ≥ 90 % of the members of the expert group) – this was generally aimed at, ‘consensus’ (75 to 89 % agreement) or ‘weak consensus’ (50 to 74 % agreement). All consented text passages are presented in boxes highlighted in grey throughout the EU Acne Guideline 2016.

10.3 Strength of recommendation (update 2016 and 2025)

To make the recommendations precise, a, standardized language was used to express the strength of recommendation throughout the EU Acne Guideline:

1) is strongly recommended

Good efficacy data, reasonable safety profile, good balance of possible benefits and harms, patient preference for the medication, high level of evidence and directness of available evidence.

2) can be recommended

Good efficacy data, good balance of possible benefits and harms, good patient acceptance, limitations with respect to the level of evidence and the directness of the evidence.

3) can be considered

Limitations with respect to efficacy and/ or limitations with respect to safety and or very relevant limitations with respect to available evidence (very little or no trials available while strong expert opinion is in favour).

4) is not recommended

Insufficient efficacy or less favourable balance of possible benefits and harms

5) may not be used under any circumstances

Harmful intervention with very unfavourable balance of possible benefits and harms

6) a recommendation for or against treatment X cannot be made at the present time (open recommendation)

Due to a lack of evidence, it is impossible to make a recommendation for or against treatment X at the present time. Insufficient data from clinical trials; promising case reports or expert opinions may exist.

10.4 Results of consensus conference (update 2025)

The consensus conference took place on March 17th, 2025 with the following experts participating (in alphabetical order): Bassel Al, Holger Brüggemann, Zrinka Bukvic Mokos, Dawn Caruana, Klaus Degitz, Clio Dessinioti, Brigitte Dréno, Rieke Driessen, Harald Gollnick, Merete Hædersdal, Alexander Katoulis, Severin Läuchli, Julien Lambert, Alison Layton, Giuseppe Micali, Falk Ochsendorf, Katarina Zak Stangeland, Simon Francis Thomsen, Daniel. All votes passed with 100% agreement of those entitled to vote (without COI).

10.5 Results of consensus conference (update 2016)

The first consensus conference took place on September 30th, 2015 with the following experts participating (in alphabetical order): Z. Bukvic Mokos, K. Degitz, B. Dréno, A. Finlay, H. Gollnick, M. Haedersdal, J. Lambert, A. Layton, H. Lomholt, F. Ochsendorf, C. Oprica. The second consensus conference took place on October 2nd with the following experts participating (in alphabetical order): Z. Bukvic Mokos, K. Degitz, A. Finlay, H. Gollnick, J. Lambert, A. Layton, J. López Estebaranz, H. Lomholt, F. Ochsendorf, C. Oprica, T. Simonart

All votes passed with a strong consensus (>75% agreement) except for the first column in the summary of recommendations for induction therapy of comedonal

acne. The voting results are shown in the following two tables (equivalent to the treatment algorithm):

Results of the consensus conference voting for therapeutic recommendations for induction therapy acne:

<table>
<tr><th></th><th>Comedonal acne</th><th>Mild-to-moderate papulopustular acne</th><th>Severe papulopustular/ moderate nodular acne</th><th>Severe nodular/ conglobate acne</th></tr>
<tr><td>High strength of recommendation</td><td rowspan="4">6 of 10 in favour</td><td rowspan="4">9 of 11 in favour</td><td rowspan="4">10 of 10 in favour</td><td rowspan="4">10 of 10 in favour</td></tr>
<tr><td>Medium strength of recommendation</td></tr>
<tr><td>Low strength of recommendation</td></tr>
<tr><td>Alternatives for females</td></tr>
</table>

Results of the consensus conference voting for therapeutic recommendations for maintenance therapy acne:

<table>
<tr><th></th><th>Comedonal acne</th><th>Mild-to-moderate papulopustular acne</th><th>Severe papulopustular/ moderate nodular acne</th><th>Severe nodular/ conglobate acne</th></tr>
<tr><td>High strength of recommendation</td><td rowspan="3">11 of 11 in favour</td><td rowspan="3">11 of 11 in favour</td><td rowspan="3">11 of 11 in favour</td><td rowspan="3">11 of 11 in favour</td></tr>
<tr><td>Medium strength of recommendation</td></tr>
<tr><td>Low strength of recommendation</td></tr>
</table>

**Alternatives for fe-males**			11 of 11 in favour

A majority voting on authorship took place considering in the following criteria: "An author is defined as an expert who contributed at least in 1 of the following 4 activities: kick-off conference, active writing, first consensus conference, second consensus conference." The criteria passed with 10 of 10 in favour of the criteria.

11 Limitations

11.1 Evidence (update 2025):

This is the third update of the European Guidelines for the Treatment of Acne. Over the time, methodological standards have changed. Due to a limitation in resources, not all previously developed parts of the guidelines, not all previous sections of the guideline could be updated to the latest methodological standards.

For many comparisons, no direct evidence was available. Indirect evidence from studies with different acne types had to be used, expert opinion had to be taken into consideration wherever data was missing or inconclusive.

Interventions used for a long time and licenced before the time of high quality RCTs often can not be supported with respective high-quality studies.

Any safety assessment based on RCT data has strong limitations, as the number of patients included in RCTs is usually too low to pick up rare adverse events. In addition, head-to-head evaluations between different treatment options are difficult, as different adverse events are difficult to compare and rate according to their relevance, especially as their severity is usually not reported. Especially for the systemic treatments, long term safety data is important to better assess the impact on microbiome, antibiotic resistance or other effects or ad-verse events that may occur or continue after treatment discontinuation.

Follow up periods are generally missing and data on relapse and maintenance of remission would be important to take into consideration as well.

Due to limitations in funding, during this update, no new search for data on patient preference with performed, however, any data reported in the newly included trials would have been taken into consideration.

Due to limited funding, not all parts of the guideline have been updated.

11.2 Evidence (update 2016):

The evaluation of available evidence was based on 10% difference. We were unable to conduct a more elaborate analysis (e.g. risk ratio calculation) due to suboptimal reporting in many publications such as missing measures of statistical dispersion.

An extensive search for newly developed guidelines published since 2011 was not performed because the dEBM team produced the first version and this is an update. The group was aware of the Canadian Acne Guideline published in 2015, which in itself was based on the 2011 EU Acne Guideline and of the Malaysian Acne Guideline 2012.

11.3 Cost considerations (update 2016)

No economic aspects were evaluated. It would have gone beyond the scope of this Guideline to consider the pricing and reimbursement regimes in every single European country. The differences throughout Europe are too large, as are those in patients' willingness and ability to pay for medication, and in the availability of generics. European guidelines are always meant to be used as a source for national and local adaptation, and pharmaco-economic considerations should be taken into account at these levels.

12 External review (update 2025)

This information will be added soon.

13 External review (update 2016)

The Guideline underwent an extensive external review. From November 16th through December 13th 2015 the Guideline was available online for comments and amendments. This period of online availability was announced using the

following mailing lists: EDF Board, EDF Guideline Committee, EDF Members, EADV Board, Union Européenne des Médecins Spécialistes (UEMS).

Additionally, every participant was encouraged to invite all potentially interested parties to review and comment on the guideline.

The EU Guidelines Group received and evaluated 74 comments. A document summarizing all comment, management and responses is available at the dEBM.

14 Dissemination and implementation (update 2025)

Dissemination and implementation will be done according the respective EuroGuiDerm SOP.

15 Dissemination and implementation (update 2016)

The Guideline will be published online on the EDF website (https://www.guidelines.edf.one/). Additionally, a short version of the Guideline alongside with this methods report (online only) will be published in the Journal of the European Academy of Dermatology and Venereology.

Furthermore, all involved experts are invited to give talks and present the results and recommendations of the Guideline at conferences.

Implementation will be pursued at a national level by local medical societies. Materials such as an online version, a short version (see above) and a therapeutic algorithm will be supplied. The EDF is planning to include the Guideline in the EDF Guidelines App.

16 Evaluation (Update 2025)

Because no further funding for this Guideline is available, no formal Europe wide evaluation program has been planned at this point. National societies are invited to evaluate the impact on a national level (e.g. assessment of awareness, treatment adhesion and patient changes).

17 Evaluation (Update 2016)

Because no further funding for this Guideline is available, no formal Europe wide evaluation program has been planned at this point. Strategies for evaluating the

impact on a national level (e.g. assessment of awareness, treatment adhesion and patient changes) are in preparation.

18 Funding and editorial independence (update 2025)

The development of this EuroGuiDerm guideline was funded through the EuroGuiDerm Centre for Guideline Development. The European Dermatology Forum (EDF) is responsible for fundraising and holds all raised funds in one account. The EuroGuiDerm Team is not involved in fundraising or in the decision making on which GL/CS development is funded. The decisions on which GL/CS is funded are made by the EuroGuiDerm Board of Directors independently. The EDF or any other body supporting guideline work is never involved in the development of this guideline and had no say on its content or focus.

19 Funding and editorial independence (update 2016)

The European S3 Guideline for the Treatment of Acne was funded by the EDF. At the beginning of the project, cooperate partners of the EDF with an interest in the field of acne were contacted and invited to provided funding for an update of the EU Guideline. The group itself was not informed about declarations of interest for support and final contributions for the cooperate partners. For means of transparency, the sources of support are declared after the finalisation of the guidelines. Contribution was given as an unrestricted educational grant to the EDF. Supporting bodies and the EDF treasurer had no influence on the guidelines contents at any stage of the guidelines development.

20 Future updates of the Guideline (update 2025)

This guideline has a validity until March 2030. The dEBM and the EDF office will be responsible to reinitiate the update in time to prolong validity or if any new information require an update sooner than 2030.

21 Future updates of the Guideline (update 2016):

In accordance with the standard operating procedures of the EDF, the European S3 Guideline for the Treatment of Acne will need to be updated after 31. December 2020. In case of new interventions being licensed or relevant new studies or reports are being published (e. g. new occurrence of highly relevant adverse

events) the EDF subcommittee on acne will evaluate the need for an earlier update.

22 Declaration and management of conflicts of interest (update 2025)

Prior to the kick-off meeting all authors and methodologists were asked to declare all personal financial conflicts of interest. All declarations were continuously updated and classified as none (with respect to acne), or personal financial conflicts of interest (see Table 6 and Appendix G Conflicts of interests of the members of the experts and methods group (update 2025).At the beginning of the consensus conference the declarations were discussed by the expert panel with respect to possible bias and consequences for the voting process. No conflicts of interest leading to the exclusion of an expert were identified. Alexander Nast is the guideline coordinator and did not vote. He does not have any personal-financial conflicts of interests.

Table 6: Conflicts of interests and voting restrictions

Personal financial conflict of interest	Name	Consequence
None	Beylot Barry, Bukvic Mokos, Brüggemann, Caruana, Degitz, Dessinioti, Driessen, Gollnick, Hædersdal, Sathyapalan, Stangeland, Töröcsik	None
Yes	Al Wattar, Dréno, Katoulis, Läuchli, Lambert, Layton, Micali, Ochsendorf, Thomsen	Voting will be deducted
Not declared	Kadurina, Sepp, Väkevä	No voting possible

23 Declaration and management of conflicts of interest (update 2016)

Prior to the kick-off meeting all authors and methodologists were asked to complete an adapted "Form for Disclosure of Potential Conflicts of Interest" of the International Committee of Medical Journal Editors (ICMJE). All declarations were continuously updated (see Appendix H) and classified as none (with respect to acne), mild/moderate [institutional or personal] (e. g. grants for research,

consultancy for scientific programs, CME talks, professional societies; received by the institution or as personal honoraria with respect to acne) or severe (e. g. employee, shareholder, patents, royalties, speakers bureaus, investor talks) conflicts of interest (see Table 6). At the beginning of the consensus conference the declarations were discussed by the expert panel with respect to possible bias. No conflicts of interest leading to the exclusion of an expert were identified.

Table 7: Classification of conflicts of interests of expert panel and methodologists

**Category**	**Names**
none	Bettoli, Lomholt, Simonart
mild/moderate institutional	Alsharif, Dressler, Erdmann, Haedersdal, Rosumeck, Werner
mild/moderate personal	Bukvic Mokos, Degitz, Dréno, Finlay, Gollnick, Lambert, Layton, López Estebaranz, Nast, Ochsendorf, Oprica
severe	*none*

24 References

1. Nast A, Rosumeck S, Erdmann R, Alsharif U, Dressler C, Werner RN. Methods report on the development of the European evidence-based (S3) guideline for the treatment of acne - update 2016. J Eur Acad Dermatol Venereol. 2016;30(8):e1-e28.
2. Brouwers MC, Kho ME, Browman GP, Burgers JS, Cluzeau F, Feder G, et al. AGREE II: advancing guideline development, reporting and evaluation in health care. CMAJ. 2010;182(18):E839-42.
3. The AGREE Reporting Checklist: a tool to improve reporting of clinical practice guidelines. Bmj. 2016;354:i4852.
4. Brouwers MC, Kerkvliet K, Spithoff K. The AGREE Reporting Checklist: a tool to improve reporting of clinical practice guidelines. Bmj. 2016;352:i1152.
5. Arbeitsgemeinschaft der Wissenschaftlichen Medizinischen Fachgesellschaften (AWMF) - Ständige Kommission Leitlinien. AWMF-Regelwerk „Leitlinien“2023 10.02.2025]; Auflage 2.1. Available from: https://www.awmf.org/regelwerk/.
6. Reynolds RV, Yeung H, Cheng CE, Cook-Bolden F, Desai SR, Druby KM, et al. Guidelines of care for the management of acne vulgaris. J Am Acad Dermatol. 2024;90(5):1006.e1-.e30.
7. Isotretinoin (Roaccutane): new safety measures to be introduced in the coming months, including additional oversight on initiation of treatment for patients under 18 years: Drug Safety Update volume 16, issue 9; 2023 [Available from: https://www.gov.uk/drug-safety-update/isotretinoin-roaccutanev-new-safety-measures-to-be-introduced-in-the-coming-months-including-additional-oversight-on-initiation-of-treatment-for-patients-under-18-years#warnings-for-sexual-dysfunction.
8. Shea BJ, Reeves BC, Wells G, Thuku M, Hamel C, Moran J, et al. AMSTAR 2: a critical appraisal tool for systematic reviews that include randomised or non-randomised studies of healthcare interventions, or both. Bmj. 2017;358:j4008.
9. Sterne JAC, Savović J, Page MJ, Elbers RG, Blencowe NS, Boutron I, et al. RoB 2: a revised tool for assessing risk of bias in randomised trials. Bmj. 2019;366:l4898.
10. Barbaric J, Abbott R, Posadzki P, Car M, Gunn LH, Layton AM, et al. Light therapies for acne. Cochrane Database Syst Rev. 2016;9(9):Cd007917.
11. Arowojolu AO, Gallo MF, Lopez LM, Grimes DA. Combined oral contraceptive pills for treatment of acne. Cochrane Database Syst Rev. 2012;2012(7):Cd004425.
12. Dressler C, Rosumeck S, Nast A. Which anti-acne treatments do patients prefer? Systematic review. (accepted for publication). J Dtsch Dermatol Ges. 2016.
13. Dressler C, Rosumeck S, Nast A. How Much Do We Know about Maintaining Treatment Response after Successful Acne Therapy? Systematic Review on the Efficacy and Safety of Acne Maintenance Therapy. Dermatology. 2016;232(3):371-80.
14. Nast A, Kopp IB, Augustin M, Banditt KB, Boehncke WH, Follmann M, et al. [S3-Guidelines for the therapy of psoriasis vulgaris]. J Dtsch Dermatol Ges. 2006;4 Suppl 2:S1-126.
15. Nast A, Dreno B, Bettoli V, Degitz K, Erdmann R, Finlay AY, et al. European evidence-based (S3) guidelines for the treatment of acne. J Eur Acad Dermatol Venereol. 2012;26 Suppl 1:1-29.

25 Appendices

25.1 Appendix A Members of the EU Guideline Group (update 2025)

Project leader and coordinator	Prof. Alexander Nast, MD<br>Division of Evidence Based Medicine (dEBM)<br>Klinik für Dermatologie<br>Charité – Universitätsmedizin Berlin<br>Charitéplatz 1<br>10117 Berlin<br>Germany<br>Tel.: +49 30 450518 313<br>Fax: +49 30 450518 977<br>E-mail: acne@debm.de<br>http://www.debm.de<br>http://www.derma.charite.de
Project office (methods group)	Antonia Pennitz, MD<br>Isabell Vader, MPH<br>Division of Evidence Based Medicine (dEBM)
Expert group	Prof. Bassel H. Al Wattar, MD (United Kingdom)<br>Prof. Marie Beylot Berry, MD (France)<br>Prof. Holger Brüggemann, MD (Denmark)<br>Prof. Zrinka Bukvic Mokos, MD, PhD (Croatia)<br>Dawn Caruana, MD (Malta)<br>Prof. Klaus Degitz, MD (Germany)<br>Clio Dessinioti, MD (Greece)<br>Prof. Brigitte Dréno, MD (France)<br>Rieke J.B. Driessen, MD (The Netherlands)<br>Prof. Harald Gollnick, MD (Germany)<br>Prof. Merete Haedersdal, MD (Denmark)<br>Prof. Miroslava Kadurina, MD (Bulgaria)<br>Prof. Alexander Katoulis, MD (Greece)<br>Prof. Julien Lambert, MD (Belgium)<br>PD Severin Läuchli, MD (Switzerland)<br>Prof. Alison Layton, MD (United Kingdom)<br>Prof Giuseppe Micali, MD (Italy)<br>Prof. Falk Ochsendorf, MD, MME (Germany)<br>Prof. Thozhukat Sathyapalan, MD (United Kingdom)<br>Prof. Norbert Sepp, MD (Austria)<br>Prof. Katarina Zak Stangeland, MD (Norway)<br>Prof. Simon Francis Thomsen, MD (Denmark)<br>Daniel Töröcsik, MD (Hungary)<br>Liisa Väkevä, MD (Finland)
Moderation of the consensus conferences	Prof. Alexander Nast, MD

25.2 Appendix B Members of the EU Guideline Group (update 2016)

Each member of the EU Guideline Group has specific responsibilities. At all stages of the guideline process, these responsibilities need to be defined.

Project leader and co-ordinator	Alexander Nast, MD<br>Division of Evidence Based Medicine (dEBM)<br>Klinik für Dermatologie<br>Charité – Universitätsmedizin Berlin<br>Chariteplatz 1<br>10117 Berlin<br>Germany<br>Tel.: +49 30 450518 313<br>Fax: +49 30 450518 977<br>E-mail: alexander.nast@charite.de<br>http://www.debm.de<br>http://www.derma.charite.de
Project office (methods group)	Stefanie Rosumeck, M.A.<br>Corinna Dressler, M.Sc., Ph. D.<br>Ricardo Niklas Werner, MD<br>Ricardo Erdmann<br>Ubai Alsharif<br>Division of Evidence Based Medicine (dEBM)
Expert group	Vincenzo Bettoli, MD (Italy)<br>Hans Bredsted Lomholt (Denmark)<br>Prof. Zrinka Bukvic Mokos, MD, PhD(Croatia)<br>Klaus Degitz, MD (Germany)<br>Brigitte Dréno, MD (France)<br>Andrew Finlay, MD (United Kingdom)<br>Harald Gollnick, MD (Germany)<br>Merete Haedersdal, MD (Denmark)<br>Julien Lambert, MD (Belgium)<br>Alison Layton, MD (United Kingdom)<br>Jose Luis Lopez Estebaranz, MD (Spain)<br>Falk Ochsendorf, MD (Germany)<br>Cristina Oprica, MD (Sweden)<br>Thierry Simonart, MD (Belgium)
Moderation of the con-sensus conferences	Alexander Nast, MD

25.3 Appendix C Search strategies (update 2025)

Search strategy for systematic reviews for acne

Database: MEDLINE via Ovid (08th January 2024)

No.	Search term	Results
1	exp Acne Vulgaris/	13647
2	acne*.ti,ab.	23613
3	1 or 2	26234

**4**	cochrane database of systematic reviews.jn. or search.tw. or meta analysis.pt. or MEDLINE.tw. or systematic review.tw.	727588
**5**	3 and 4	979

Database: Embase via Ovid (08th January 2024)

No.	Search term	Results
**1**	acne fulminans/ or acne vulgaris/ or acne cystica/ or acne conglobata/	16141
**2**	acne*.ti,ab.	36535
**3**	1 or 2	39470
**4**	(meta analysis or systematic review or MEDLINE).tw.	587655
**5**	3 and 4	887

Search strategy for systematic reviews for long-term safety of antibiotics

Database: Medline via Ovid (27th February 2024)

No.	Search term	Results
**1**	ae.fs.	2035329
**2**	safe*.ti,ab.	1192925
**3**	de.fs.	3164215
**4**	adverse.ti,ab.	702515
**5**	co.fs.	2272012
**6**	side effect*.ti,ab.	308115
**7**	complication*.ti,ab.	1154178
**8**	ci.fs.	659501
**9**	tolerated.ti,ab.	162305
**10**	tolerance.ti,ab.	287540
**11**	harm*.ti,ab.	258076
**12**	toxicity.ti,ab.	474700
**13**	risk.ti.	608713
**14**	Pregnancy complications/dt	8795
**15**	Clinical Trial, Phase IV.pt.	2479

No.	Search term	Results
16	Drug hypersensitivity/	25267
17	Tolerability.ti,ab.	60948
18	to.fs.	488886
19	toxicology/	27287
20	Drug induced.ti,ab.	45276
21	Negative effects.ti,ab.	40338
22	or/1-21	9868742
23	(Microbiome* or microbiomi* or Microflora* or Microbiota* or Microbial*).ti,ab.	345440
24	microbiota/ or gastrointestinal microbiome/	76111
25	Drug Resistance, Bacterial/ or Tetracycline Resistance/	51213
26	(Resistanc* or resistant* or susceptib*).ti,ab.	1639674
27	or/23-26	1950095
**28**	**22 or 27**	**11041008**
29	Erythromycin*.ti,ab.	23583
30	erythromycin/ or azithromycin/ or erythromycin estolate/ or erythromycin ethylsuccin-ate/	20999
31	tetracyclines/ or tetracycline/	26449
32	Tetra#yclin*.ti,ab.	45191
33	Doxycycline/	11024
34	Doxy#yclin*.ti,ab.	17160
35	Minocycline/	6609
36	Mino#yclin*.ti,ab.	8200
37	Lymecycline/	131
38	Lyme#yclin*.ti,ab.	137
39	Azithromycin*.ti,ab.	12032
40	Macrolides/	14113
41	Ma#rolide*.ti,ab.	19403
42	Sare#yclin*.ti,ab.	63
43	94O110CX2E.rn.	34
44	or/29-43	127583

No.	Search term	Results
**45**	**28 and 44**	**85664**
**46**	cochrane database of systematic reviews.jn. or search.tw. or meta analysis.pt. or MEDLINE.tw. or systematic review.tw.	737134
47	45 and 46	1988

Database: Embase via Ovid (27th February 2024)

No.	Search term	Results
**1**	ae.fs.	1384720
**2**	safe*.ti,ab,kw.	1796985
**3**	Adverse.ti,ab,kw,ox.	1350009
**4**	po.fs.	723458
**5**	co.fs.	1891314
**6**	exp adverse drug reaction/	654120
**7**	Complication*.ti,ab.	1704775
**8**	Drug safety/	569410
**9**	to.fs.	608939
**10**	Side effect*.ti,ab.	455567
**11**	Risk.ti.	844511
**12**	Tolerated.ti,ab.	274367
**13**	Tolerance.ti,ab.	365579
**14**	Harm.ti,ab.	90476
**15**	Side reaction*.ti,ab.	5540
**16**	Drug withdrawal/	264659
**17**	Health risks.ti,ab.	34489
**18**	Potential risks.ti,ab.	18487
**19**	Toxic effects.ti,ab.	52645
**20**	Toxicity.ti,ab.	657707
**21**	Toxicities.ti,ab.	106178
**22**	or/1-21	8708777
**23**	bacterial microbiome/	2414

No.	Search term	Results
24	microflora/	33729
25	bacterial flora/ or feces microflora/ or intestine flora/ or lung microbiota/ or mouth flora/ or skin flora/ or vagina flora/	133736
26	(Microbiome* or Microflora* or Microbiota* or Microbial*).ti,ab.	398136
27	(resistanc* or resistant* or susceptib*).ti,ab.	2032610
28	antibiotic resistance/ or tetracycline resistance/ or exp macrolide resistance/	202706
29	or/23-28	2449942
**30**	**22 or 29**	**10577351**
31	Erythromycin*.ti,ab.	27527
32	erythromycin stearate/ or erythromycin/ or erythromycin ethylsuccinate/ or erythromycin estolate/ or erythromycin plus isotretinoin/	84907
33	tetracycline derivative/ or tetracycline/	106047
34	Tetra#yclin*.ti,ab.	49627
35	doxycycline/	69369
36	Doxy#yclin*.ti,ab.	26017
37	minocycline/	29609
38	Mino#yclin*.ti,ab.	11124
39	lymecycline/	858
40	Lyme#yclin*.ti,ab.	186
41	azithromycin/	54873
42	Azithromycin*.ti,ab.	19148
43	macrolide/	38632
44	Ma#rolide*.ti,ab.	25413
45	Sare#yclin*.ti,ab.	96
46	sarecycline/	149
47	or/31-46	302892
**48**	**30 and 47**	**190058**
49	(meta analysis or systematic review or MEDLINE).tw.	594898
**50**	**48 and 49**	**3701**

Search strategy for RCTs on the new topical treatments trifarotene and clascoterone

Database: Medline (09th August 2024)

No.	Search term	Results
1	Trifaroten*.mp.	61
2	Clascoteron*.mp.	56
3	1 or 2	110

Database: Embase (09th August 2024)

No.	Search term	results
1	trifarotene/	136
2	Trifaroten*.ti,ab,kf.	79
3	clascoterone/	126
4	Clascoteron*.ti,ab,kf.	64
5	or/1-4	244

Search strategy for RCTs on acne after the last search date of the AAD Guidelines

Database: Medline via Ovid (23rd August 2024)

No	Search term	results
1	exp randomized controlled trial/	621147
2	controlled clinical trial.pt.	95592
3	randomized.ab.	657523
4	placebo.ab.	251021
5	clinical trials as topic.sh.	203120
6	randomly.ab.	440380
7	trial.ti.	316118
8	or/1-7	1621908
9	exp animals/ not humans.sh.	5251228
10	8 not 9	1494471
11	exp Acne Vulgaris/	13987
12	acne*.ti,ab.	24515

No	Search term	results
13	11 or 12	27161
14	10 and 13	2981
15	limit 14 to yr="2022 -Current"	424

Database: Cochrane CENTRAL trials database (23rd August 2024)

ID	Search	Hits
#1	(acne*):ti,ab,kw (Word variations have been searched)	6162
#2	MeSH descriptor: [Acne Vulgaris] explode all trees	1901
#3	#1 or #2 with Publication Year from 2022 to 2024, in Trials	923

25.4 Appendix D Search strategies (update 2016)

Search strategy for topical and systemic treatments:

Databases: Ovid MEDLINE®

#	**Search Statement**
1.	exp acne/
2.	"acne*".ab,ti.
3.	1 or 2
4.	exp benzoyl peroxide/
5.	"benzoyl peroxid*".ab,ti.
6.	exp Retinoids/
7.	"retinoid*".ab,ti.
8.	exp Naphthalenes/
9.	"adapalene".ab,ti.
10.	exp isotretinoin/
11.	"isotretinoin".ab,ti.
12.	exp retinoic acid/
13.	"tretinoin".ab,ti.

#	Search Statement
14.	exp dicarboxylic acids/
15.	"azelaic acid".ab,ti.
16.	exp zinc/
17.	"zinc".ab,ti.
18.	exp Antibiotics, Antitubercular/
19.	exp clindamycin/
20.	"clindamycin*".ab,ti.
21.	exp doxycycline/
22.	"doxycyclin*".ab,ti.
23.	exp erythromycin/
24.	"erythromycin*".ab,ti.
25.	exp lymecycline/
26.	"lymecyclin*".ab,ti.
27.	exp minocycline/
28.	"minocyclin*".ab,ti.
29.	"nadifloxacin".ab,ti.
30.	exp tetracycline/
31.	"tetracyclin*".ab,ti.
32.	4 or 5 or 6 or 7 or 8 or 9 or 10 or 11 or 12 or 13 or 14 or 15 or 16 or 17 or 18 or 19 or 20 or 21 or 22 or 23 or 24 or 25 or 26 or 27 or 28 or 29 or 30 or 31
33.	Randomized Controlled Trials as Topic/
34.	randomized controlled trial/
35.	Random Allocation/
36.	Double-Blind Method/
37.	Single Blind Method/
38.	clinical trial/

#	**Search Statement**
39.	clinical trial, phase I.pt.
40.	clinical trial, phase II.pt.
41.	clinical trial, phase III.pt.
42.	clinical trial, phase IV.pt.
43.	controlled clinical trial.pt.
44.	randomized controlled trial.pt.
45.	multicenter study.pt.
46.	clinical trial.pt.
47.	exp Clinical Trials as topic/
48.	33 or 34 or 35 or 36 or 37 or 38 or 39 or 40 or 41 or 42 or 43 or 44 or 45 or 46 or 47
49.	(clinical adj trial$).tw.
50.	((singl$ or doubl$ or treb$ or tribl$) adj (blind$3 or mask$3)).tw.
51.	Placebos/
52.	placebo$.tw.
53.	randomly allocated.tw.
54.	(allocated adj2 random$).tw.
55.	49 or 50 or 51 or 52 or 53 or 54
56.	48 or 55
57.	case report.tw.
58.	letter/
59.	historical article/
60.	57 or 58 or 59
61.	56 not 60
62.	3 and 32 and 61
63.	limit 62 to yr="2010 -Current"

25.5 Appendix E Methodology checklist – Barbaric et al. (Preview, date: 16. May 2015) (update 2016)

S I G N	**Methodology Checklist 1: Systematic Reviews and Meta-analyses**<br><br>SIGN gratefully acknowledges the permission received from the authors of the AMSTAR tool to base this checklist on their work: *Shea BJ, Grimshaw JM, Wells GA, Boers M, Andersson N, Hamel C,. et al. Development of AMSTAR: a measurement tool to assess the methodological quality of systematic reviews. BMC Medical Research Methodology 2007, **7**:10 doi:10.1186/1471-2288-7-10. Available from* [*http://www.biomedcentral.com/1471-2288/7/10*](http://www.biomedcentral.com/1471-2288/7/10) *[cited 10 Sep 2012]*

Study identification (*Include author, title, year of publication, journal title, pages*)<br><br>Barbaric J, Abbott R, Car M, Gunn LH, Layton AM, Majeed A, Car J. Light therapies for acne. *Cochrane Database of Systematic Reviews* 2015, Issue 7. Art. No.: CD007917. DOI: 10.1002/14651858.CD007917.pub2 (Preview, date: 16. May 2015)

Guideline topic: European Evidence-based (S3) Guideline for the treatment of acne (ICD L70.0) Update 2016	Key Question No: Which kind of light therapies is effective to treat acne patients?

**Before** completing this checklist, consider:<br><br>Is the paper relevant to key question? Analyse using PICO (Patient or Population Intervention Comparison Outcome). IF NO reject. IF YES complete the checklist.
Checklist completed by: Stefanie Rosumeck, M.A. and Corinna Dressler, M.Sc., Ph.D.
**SECTION 1: INTERNAL VALIDITY**

**In a well conducted systematic review:**		**Does this study do it?**	
1.1	The research question is clearly defined and the inclusion/ exclusion criteria must be listed in the paper.	Yes X<br><br>**If no reject**	No □
1.2	A comprehensive literature search is carried out.	Yes X<br><br>Not applicable<br><br>**If no reject**	No □<br><br>□
1.3	At least two people should have selected studies.	Yes X	No □<br><br>Can't say □
1.4	At least two people should have extracted data.	Yes X	No □<br><br>Can't say □
1.5	The status of publication was not used as an inclusion criterion.	Yes X	No □
1.6	The excluded studies are listed.	Yes X	No □

1.7	The relevant characteristics of the included studies are provided.	Yes X No □
1.8	The scientific quality of the included studies was assessed and reported.	Yes X No □
1.9	Was the scientific quality of the included studies used appropriately?	Yes X No □
1.10	Appropriate methods are used to combine the individual study findings.	Yes X No □<br>Can't say □ Not applicable □
1.11	The likelihood of publication bias was assessed appropriately.	Yes □ No □<br>Not applicable X
1.12	Conflicts of interest are declared.	Yes X No □
**SECTION 2: OVERALL ASSESSMENT OF THE STUDY**		
2.1	What is your overall assessment of the methodological quality of this review?	High quality (++) X<br>Acceptable (+) □<br>Low quality (-)□<br>Unacceptable – reject 0 □
2.2	Are the results of this study directly applicable to the patient group targeted by this guideline?	Yes X No □
2.3	**Notes:**<br>1.10. Only 3 RCT could be combined for a meta-analysis. Other included studies were reported narratively<br>1.11. Authors planned to test for publication bias, but the number of included trials was too low for a funnel plot.	

25.6 Appendix F Methodology checklist – Arowojolu et al. (2012) (update 2016)

SIGN	**Methodology Checklist 1: Systematic Reviews and Meta-analyses**<br>SIGN gratefully acknowledges the permission received from the authors of the AMSTAR tool to base this checklist on their work: *Shea BJ, Grimshaw JM, Wells GA, Boers M, Andersson N, Hamel C,. et al. Development of AMSTAR: a measurement tool to assess the methodological quality of systematic reviews. BMC Medical Research Methodology 2007,* ***7****:10 doi:10.1186/1471-2288-7-10. Available from http://www.biomedcentral.com/1471-2288/7/10 [cited 10 Sep 2012]*

Study identification (*Include author, title, year of publication, journal title, pages*)<br><br>Arowojolu AO, Gallo MF, Lopez LM, Grimes DA. Combined oral contraceptive pills for treatment of acne. *Cochrane Database of Systematic Reviews* 2012, Issue 7. Art. No.: CD004425. DOI: 10.1002/14651858.CD004425.pub6.			
Guideline topic: European Evidence-based (S3) Guideline for the treatment of acne (ICD L70.0) Update 2016		Key Question No: Should combined oral contraceptive pills be used to treat acne in female patients?	
**Before** completing this checklist, consider:<br><br>Is the paper relevant to key question? Analyse using PICO (Patient or Population Intervention Comparison Outcome). IF NO reject. IF YES complete the checklist.			
Checklist completed by: Stefanie Rosumeck, M.A. and Corinna Dressler, M.Sc., Ph.D.			
**SECTION 1: INTERNAL VALIDITY**			
**In a well conducted systematic review:**		**Does this study do it?**	
1.1	The research question is clearly defined and the inclusion/ exclusion criteria must be listed in the paper.	Yes X<br><br>**If no reject**	No □
1.2	A comprehensive literature search is carried out.	Yes X<br><br>Not applicable<br><br>**If no reject**	No □<br><br>□
1.3	At least two people should have selected studies.	Yes □	No X<br><br>Can't say □
1.4	At least two people should have extracted data.	Yes X	No □<br><br>Can't say □
1.5	The status of publication was not used as an inclusion criterion.	Yes X	No □
1.6	The excluded studies are listed.	Yes X	No □
1.7	The relevant characteristics of the included studies are provided.	Yes X	No □
1.8	The scientific quality of the included studies was assessed and reported.	Yes X	No □
1.9	Was the scientific quality of the included studies used appropriately?	Yes X	No □
1.10	Appropriate methods are used to combine the individual study findings.	Yes X	No □

		Can't say ◻	Not applicable ◻
1.11	The likelihood of publication bias was assessed appropriately.	Yes ◻<br>Not applicable	No ◻<br>X
1.12	Conflicts of interest are declared.	Yes X	No ◻
**SECTION 2: OVERALL ASSESSMENT OF THE STUDY**			
2.1	What is your overall assessment of the methodological quality of this review?	High quality (++) ◻<br>Acceptable (+) X<br>Low quality (-)◻<br>Unacceptable – reject 0 ◻	
2.2	Are the results of this study directly applicable to the patient group targeted by this guideline?	Yes X	No ◻
2.3	**Notes:**<br>1.3: only one author assessed identified title and abstracts<br>1.8: Assessment was done according to Higgins 2011 ("adequacy of sample size, randomization protocol, allocation concealment, inclusion and exclusion criteria, blinding, the extent of premature withdrawals and loss to follow up and method of analysis"); only 'allocation concealment' was noted for each study in 'Characteristics of included studies' section; an overall summary stated in the 'Risk of bias in included studies' paragraph somewhat allows for a judgement of the separate studies if desired<br>1.9.: No specific sensitivity analysis were performed but statement in paragraph 'Quality of the evidence'<br>1.10.: Very few studies were combined in meta-analyses. Some of these were pooled in spite of statistical heterogeneity (e.g. Analysis 3.3. I^2=79%, Analysis 13.1. I^2=71%, Analysis 13.2 I^2=68%); no explanation for heterogeneity, sensitivity analysis or meta-regression was provided – unclear why no random-effects models were chosen<br>1.11.: less than 10 publication per comparison<br>1.12.: Authors attempted to extract financial support of each included study; only one author of the systematic review declared conflicts of interest; no funding for preparation of the systematic review		

25.7 Appendix G Conflicts of interests of the members of the experts and methods group (update 2025)

Title	First name	Last name	Personal financial conflicts of interst
Prof.	Bassel H.	Al Wattar	Besin, Theramex, Gedeon Richter Exeltis (honorarium for providing unrestricted educational events)

Prof.	Marie	Beylot Barry	None
Prof.	Zrinka	Bukvic Mokos	None
Prof.	Holger	Brügge-mann	None
Dr.	Dawn	Caruana	Paid speaker for two educational meetings by CeraVe and La Roche Posay in 2024
Prof.	Klaus	Degitz	None
Dr.	Clio	Dessini-oti	None
Dr.	Rieke J. B.	Driessen	None
Prof.	Brigitte	Dréno	Galderma (board member and speaker), Fabre (board mem-ber and speaker), La Roche-Posay (board member and speaker)
Prof.	Harald	Gollnick	None
Prof.	Merete	Hæders-dal	- Personal financial: none<br>- Equipment, research collaboration and lecturing: Cyno-sure-Lutronic<br>- Research collaboration: Damae, Leo Pharma, La Roche-Posay, Procter & Gamble<br>- Equipment: GM Medical<br>- Equipment, research collaboration: Michelson Diagnos-tics, Venus Concept
Prof.	Miroslava	Kadurina	2022: None, 2025: Not declared
Prof.	Alexander	Katoulis	UCB, Janssen, Pharmaserve Lilly, Amgen, AbbVie, Genesis Pharma, Leo Pharma, Pfizer, L' Oreal, Galderma, Pierre Fa-bre, Novartis (Honoraria for lectures, advisory boards and travel grants)
PD	Severin	Läuchli	Galderma (speaker honorarium), Urgo (advisory board), Almi-rall (travel grant), Novartis (advisory board), Abbvie (advisory board, speaker honorarium), Sandoz (advisory board)
Prof.	Julien	Lambert	Mylan and Galderma (advisory board member) Roche-Posay (speaker)
Prof.	Alison	Layton	Galderma Pharma, LEO Pharma, La Roche-Posay, L'Oréal, Viatris, Novartis, Origimm Biotechnology, Beiersdorf and Sanofi (honoraria for acting as a consultant, attending

			advisory boards or providing unrestricted presentations involved in educational events and national and international meetings which have involved discussing dermocosmetics, active topical and oral agents for acne and the development of an acne vaccine)
Prof.	Giuseppe	Micali	Galderma (speaker honorarium), L'Oréal (speaker honorarium), Viatris (speaker honorarium)
Prof.	Alexander	Nast	none
Prof.	Falk	Ochsendorf	Mylan, Pierre-Fabre, Infectopharma, Derma-Live (speaker fees for delivering scientific content during educational meetings, advisory board meetings)
Prof.	Thozhukat	Sathyapalan	None
Prof.	Norbert	Sepp	Not declared
Prof.	Katarina Zak	Stangeland	None
Prof.	Simon Francis	Thomsen	AbbVie, Almirall, Boehringer, CSL, Eli Lilly, Galderma, Incyte, Janssen, LEO Pharma, Novartis, Pfizer, Sanofi, Servier, Symphogen, UCB, Union Therapeutics (speaker/advisory board/consultant)
Dr.	Daniel	Töröcsik	Beiersdorf (honoraria for consulting on research activities)
Dr.	Liisa	Väkevä	2022: None, 2025: Not declared
	Martin	Dittmann	None
Dr.	Antonia	Pennitz	none
	Isabell	Vader	none

25.8 Appendix H Conflicts of interests of the members of the experts and methods group (update 2016)

		Ubai Alsharif	Vincenzo Bettoli	Zrinka Bukvic Mokos	Klaus Degitz
**1**	**Work Under Consideration for these Guidelines**				

		Ubai Alsharif	Vincenzo Bettoli	Zrinka Bukvic Mokos	Klaus Degitz
1.1	Grant	European Dermatology Forum for evidence generation and project coordination	No	No	No
1.2	Consulting fee or honorarium	No	No	No	No
1.3	Support for travel to meetings for the study or other purposes	No	No	No	No
1.4	Fees for participation in review activities such as data-monitoring boards, statistical analysis, end-point committees, and the like	European Dermatology Forum for evidence generation and project coordination	No	No	No
1.5	Payment for writing or reviewing the manuscript	European Dermatology Forum for evidence generation and project coordination	No	No	No
1.6	Provision of writing assistance, medicines, equipment, or administrative support	No	No	No	No
1.7	Other	No	No	No	No
**2**	**Relevant Financial Activities Outside Submitted Work**				
2.1	Board membership	No	No	No	STIEFEL in 2012
2.2	Consultancy	No	No	No	No
2.3	Employment	No	No	No	No
2.4	Expert testimony	No	No	No	No
2.5	Grants/grants pending	dEBM has received research grants from Pfizer (systematic review on psoriasis maintenance therapy) and	No	No	No

		**Ubai Alsharif**	**Vincenzo Bettoli**	**Zrinka Bukvic Mokos**	**Klaus Degitz**
		GlaxoSmithKline (systematic review on time until onset of action of treatments for acne vulgaris). GlaxoSmithKline is a manufacturer of anti-acne treatments (BPO/clindamycin); dEBM has received compensation for participation in a clinical trial on scar treatment from MERZ; dEBM has a pending grant from MEDA for a systematic review outside of the field of acne			
2.6	Payment for lectures including service on speakers bureaus	No	No	GlaxoSmithKline for lecture titled 'Current Concepts on the Pathogenesis of Acne' (lecture in Croatian language) in Zagreb on February 14, 2014 for Croatian dermatologists	Stiefel, MEDA Pharma
2.7	Payment for manuscript preparation	No	No	No	Stiefel for an interview

		Ubai Alsharif	Vincenzo Bettoli	Zrinka Bukvic Mokos	Klaus Degitz
2.8	Patents (planned, pending, or issued)	No	No	No	No
2.9	Royalties	No	No	No	No
2.10	Payment for development of educational presentations	No	No	No	No
2.11	Stock/stock options	No	No	No	No
2.12	Travel, accommodations, and meeting expenses unrelated to activities listed	No	No	No	No
2.13	Other (err on the side of full disclosure)	No	No	No	No
**3**	**Other relationships**	No	No	No	No

		Brigitte Dréno	Corinna Dressler	Ricardo Erdmann
**1**	**Work Under Consideration for these Guidelines**			
1.1	Grant	No	European Dermatology Forum for evidence generation and project coordination	European Dermatology Forum for evidence generation and project coordination
1.2	Consulting fee or honorarium	No	No	No
1.3	Support for travel to meetings for the study or other purposes	No	No	No
1.4	Fees for participation in review activities such as data-monitoring boards, statistical analysis, end-point committees, and the like	No	European Dermatology Forum for evidence generation and project coordination	European Dermatology Forum for evidence generation and project coordination

		Brigitte Dréno	Corinna Dressler	Ricardo Erdmann
1.5	Payment for writing or reviewing the manuscript	No	European Dermatology Forum for evidence generation and project coordination	European Dermatology Forum for evidence generation and project coordination
1.6	Provision of writing assistance, medicines, equipment, or administrative support	No	No	No
1.7	Other	No	No	No
**2**	**Relevant Financial Activities Outside Submitted Work**			
2.1	Board membership	Galderma, MEDA Pharma, Pierre Fabre Pharma, La Roche-Posay	No	No
2.2	Consultancy	No	No	No
2.3	Employment	No	No	No
2.4	Expert testimony	No	No	No
2.5	Grants/grants pending	Galderma, Pierre Fabre Pharma	dEBM has received research grants from Pfizer (systematic review on psoriasis maintenance therapy) and GlaxoSmithKline (systematic review on time until onset of action of treatments for acne vulgaris). GlaxoSmithKline is a manufacturer of anti-acne treatments	dEBM has received research grants from Pfizer (systematic review on psoriasis maintenance therapy) and GlaxoSmithKline (systematic review on time until onset of action of treatments for acne vulgaris). GlaxoSmithKline is a manufacturer of anti-acne treatments (BPO/clindamycin);

		**Brigitte Dréno**	**Corinna Dressler**	**Ricardo Erdmann**
			(BPO/clindamycin); dEBM has received compensation for participation in a clinical trial on scar treatment from MERZ; dEBM has a pending grant from MEDA for a systematic review outside of the field of acne	dEBM has received compensation for participation in a clinical trial on scar treatment from MERZ; dEBM has a pending grant from MEDA for a systematic review outside of the field of acne
2.6	Payment for lectures including service on speakers bureaus	Galderma, MEDA Pharma, Pierre Fabre Pharma, La Roche-Posay	No	No
2.7	Payment for manuscript preparation	No	No	No
2.8	Patents (planned, pending, or issued)	No	No	No
2.9	Royalties	No	No	No
2.10	Payment for development of educational presentations	Galderma, Pierre Fabre Pharma, La Roche-Posay	No	No
2.11	Stock/stock options	No	No	No
2.12	Travel, accommodations, and meeting expenses unrelated to activities listed	No	No	No
2.13	Other (err on the side of full disclosure)	No	No	No

		Brigitte Dréno	Corinna Dressler	Ricardo Erdmann
**3**	**Other relationships**	No	No	No

		Andrew Y. Finlay	Harald Gollnick	Merete Hædersdal	Julien Lambert	Alison Layton
**1**	**Work Under Consideration for these Guidelines**					
1.1	Grant	No	No	No	No	No
1.2	Consulting fee or honorarium	No	No	No	No	No
1.3	Support for travel to meetings for the study or other purposes	No	No	No	No	No
1.4	Fees for participation in review activities such as data-monitoring boards, statistical analysis, end-point committees, and the like	No	No	No	No	No
1.5	Payment for writing or reviewing the manuscript	No	No	No	No	No
1.6	Provision of writing assistance, medicines, equipment, or administrative support	No	No	No	No	No
1.7	Other	No	No	No	No	No
**2**	**Relevant Financial Activities Outside Submitted Work**					
2.1	Board membership	No	European Dermatology Forum, German Dermatological Society	No	MEDA, Galderma, Pierre Fabre	No
2.2	Consultancy	Galderma Global Alliance (World and European),	No	No	MEDA	GlaxoSmithKline, MEDA Pharma, Galderma,

		Andrew Y. Finlay	Harald Gollnick	Merete Hædersdal	Julien Lambert	Alison Layton
		Novartis Advisory Board meetings, Napp Advisory Board, Archimedes Advisory Board, Amgen Advisory Board				L'Oréal on an ad hoc basis
2.3	Employment	No	No	No	No	Harrogate and District NHS Foundation Trust, Consultant Dermatologist, self employed private practitioner as Consultant Dermatologist, working at BMI Duchy Hospital, Harrogate
2.4	Expert testimony	No	No	No	No	No
2.5	Grants/grants pending	No	No	Almirall, Galderma, Leo Pharma, Lumenis, Lutronic, Procter & Gamble	No	GlaxoSmithKline, Galderma

		**Andrew Y. Finlay**	**Harald Gollnick**	**Merete Hædersdal**	**Julien Lambert**	**Alison Layton**
2.6	Payment for lectures including service on speakers bureaus	No	GlaxoSmithKline, Galderma, Intendis, Novartis, IMTM	Galderma	MEDA	Galderma, GlaxoSmithKline, MEDA Pharma
2.7	Payment for manuscript preparation	No	No	No	No	No
2.8	Patents (planned, pending, or issued)	No	No	Leo Pharma	No	No
2.9	Royalties	Joint copyright owner of the DLQI, CDLQI and FDLQI. Royalties go to Cardiff University	No	No	No	No
2.10	Payment for development of educational presentations	No	No	No	No	GlaxoSmithKline, MEDA Pharma, Galderma
2.11	Stock/stock options	No	No	No	No	No
2.12	Travel, accommodations, and meeting expenses unrelated to activities listed	No	No	No	No	No
2.13	Other (err on the side of full disclosure)	Joint copyright owner of the Cardiff Acne Disability Index. This is freely available with no charge.	No	Loan of laser devices from Candela/ Syneron and Palomarc/ Cynosure	No	Act as CI/PI for a number of NIHR clinical trials some of wich are sponsored by pharmaceutical

		Andrew Y. Finlay	Harald Gollnick	Merete Hædersdal	Julien Lambert	Alison Layton
						companies (Galderma, Leo Pharma, Intendis, Wyeth, Novartis). Resource supporting these studies is provided to the NHS Organisation I work for
3	**Other relationships**	No	No	No	No	Member of the Global Alliance and European Acne Panel. This is supported by unrestricted educational grants from Galderma. The groups embrace many international dermatologists who work to improve outcomes for acne and report on recent clinical trials.

		Hans Bredsted Lomholt	Jose Luis López Este-baranz	Alexander Nast	Falk Ochsendorf
**1**	**Work Under Consideration for these Guidelines**				
1.1	Grant	No	No	European Dermatology Forum for evidence gener-ation and project coordina-tion	No
1.2	Consulting fee or honorarium	No	No	No	No
1.3	Support for travel to meetings for the study or other pur-poses	No	No	No	No
1.4	Fees for participation in re-view activities such as data-monitoring boards, statistical analysis, end-point commit-tees, and the like	No	No	European Dermatology Forum for evidence gener-ation and project coordina-tion	No
1.5	Payment for writing or re-viewing the manuscript	No	No	European Dermatology Forum for evidence gener-ation and project coordina-tion	No
1.6	Provision of writing assis-tance, medicines, equipment, or administrative support	No	No	No	No
1.7	Other	No	No	No	No
**2**	**Relevant Financial Activities Outside Submitted Work**				
2.1	Board membership	No	No	No	No
2.2	Consultancy	No	Galderma, MEDA Pharma	No	Expert mee-tings: Vichy Laboratories, MEDA Pharma, Gla-xoSmithKline Stiefel Pharma,

		**Hans Bredsted Lomholt**	**Jose Luis López Este-baranz**	**Alexander Nast**	**Falk Ochsendorf**
					Galderma La-boratories
2.3	Employment	No	No	No	No
2.4	Expert testimony	No	No	No	No
2.5	Grants/grants pending	No	No	dEBM has received re-search grants from Pfizer (systematic review on pso-riasis maintenance ther-apy) and GlaxoSmithKline (systematic review on time until onset of action of treatments for acne vul-garis). GlaxoSmithKline is a manufacturer of anti-acne treatments (BPO/clindamycin); dEBM has received compensa-tion for participation in a clinical trial on scar treat-ment from MERZ; dEBM has a pending grant from MEDA for a systematic re-view outside of the field of acne	No
2.6	Payment for lectures includ-ing service on speakers bu-reaus	No	MEDA Pharma, Bayer, Gla-xoSmithKline	Intendis/Bayer Healthcare, Pfizer, Boehringer Ingel-heim, Novartis, MEDA, Bi-ogen Idec, Abbott (now Abbvie)	Lectures dur-ing con-gresses/CME activities: MEDA Pharma, Glax-oSmithKline Stiefel

		**Hans Bredsted Lomholt**	**Jose Luis López Este-baranz**	**Alexander Nast**	**Falk Ochsendorf**
					Pharma, Gal-derma Labor-atories
2.7	Payment for manuscript pre-paration	No	No	No	No
2.8	Patents (planned, pending, or issued)	No	No	No	No
2.9	Royalties	No	No	No	No
2.10	Payment for development of educational presentations	No	No	No	No
2.11	Stock/stock options	No	No	No	No
2.12	Travel, accommodations, and meeting expenses unre-lated to activities listed	No	No	No	No
2.13	Other (err on the side of full disclosure)	No	No	No	No
**3**	**Other relationships**	No	No	No	No

		**Christina Oprica**	**Stefanie Rosumeck**	**Thierry Simonart**
**1**	**Work Under Consideration for these Guidelines**			
1.1	Grant	No	European Dermatology Forum for evidence generation and pro-ject coordination	No
1.2	Consulting fee or honorarium	No	No	No
1.3	Support for travel to meetings for the study or other pur-poses	No	No	No

		Christina Oprica	Stefanie Rosumeck	Thierry Simonart
1.4	Fees for participation in review activities such as data-monitoring boards, statistical analysis, end-point committees, and the like	No	European Dermatology Forum for evidence generation and project coordination	No
1.5	Payment for writing or reviewing the manuscript	No	European Dermatology Forum for evidence generation and project coordination	No
1.6	Provision of writing assistance, medicines, equipment, or administrative support	No	No	No
1.7	Other	No	No	No
**2**	**Relevant Financial Activities Outside Submitted Work**			
2.1	Board membership	No	No	No
2.2	Consultancy	No	No	No
2.3	Employment	No	No	No
2.4	Expert testimony	No	No	No
2.5	Grants/grants pending	No	dEBM has received research grants from Pfizer (systematic review on psoriasis maintenance therapy) and GlaxoSmithKline (systematic review on time until onset of action of treatments for acne vulgaris). GlaxoSmithKline is a manufacturer of anti-acne treatments (BPO/clindamycin); dEBM has received compensation for participation in a clinical trial on scar treatment from	No

		Christina Oprica	Stefanie Rosumeck	Thierry Simonart
			MERZ; dEBM has a pending grant from MEDA for a systematic review outside of the field of acne	
2.6	Payment for lectures including service on speakers bureaus	Galderma symposium in Stockholm and Oslo	No	No
2.7	Payment for manuscript preparation	No	No	No
2.8	Patents (planned, pending, or issued)	No	No	No
2.9	Royalties	No	No	No
2.10	Payment for development of educational presentations	No	No	No
2.11	Stock/stock options	No	No	No
2.12	Travel, accommodations, and meeting expenses unrelated to activities listed	No	No	No
2.13	Other (err on the side of full disclosure)	No	No	No
**3**	**Other relationships**	No	No	No

		Ricardo N. Werner
**1**	**Work Under Consideration for these Guidelines**	
1.1	Grant	European Dermatology Forum for evidence generation and project coordination
1.2	Consulting fee or honorarium	No
1.3	Support for travel to meetings for the study or other purposes	No
1.4	Fees for participation in review activities such as data-monitoring	European Dermatology Forum for evidence generation and project coordination

		**Ricardo N. Werner**
	boards, statistical analysis, end-point committees, and the like	
1.5	Payment for writing or reviewing the manuscript	European Dermatology Forum for evidence generation and project coordination
1.6	Provision of writing assistance, medicines, equipment, or administrative support	No
1.7	Other	No
**2**	**Relevant Financial Activities Outside Submitted Work**	
2.1	Board membership	No
2.2	Consultancy	No
2.3	Employment	No
2.4	Expert testimony	No
2.5	Grants/grants pending	dEBM has received research grants from Pfizer (systematic review on psoriasis maintenance therapy) and GlaxoSmithKline (systematic review on time until onset of action of treatments for acne vulgaris). GlaxoSmithKline is a manufacturer of anti-acne treatments (BPO/clindamycin); dEBM has received compensation for participation in a clinical trial on scar treatment from MERZ; dEBM has a pending grant from MEDA for a systematic review outside of the field of acne
2.6	Payment for lectures including service on speakers bureaus	No
2.7	Payment for manuscript preparation	No
2.8	Patents (planned, pending, or issued)	No
2.9	Royalties	No
2.10	Payment for development of educational presentations	No
2.11	Stock/stock options	No

		**Ricardo N. Werner**
2.12	Travel, accommodations, and meeting expenses unrelated to activities listed	No
2.13	Other (err on the side of full dis-closure)	No
**3**	**Other relationships**	No
