# Supplementary material for: Update of the EuroGuiDerm evidence‐based guideline for the treatment of acne—Short version
Source: J Eur Acad Dermatol Venereol. 2026 Mar 18;40(7):1162–72. doi: 10.1111/jdv.70331 (PMC13308670; doi:10.1111/jdv.70331)
Supplement: Supplementary file 3 — Data S3. [file JDV-40-1162-s002.pdf]

Table of contents

Key question 1a: Isotretinoin vs. antibiotics: Efficacy 2
Key question 1b: Safety of long term antibiotic use 14
Key question 2: Hormonal treatments 29
Key question 3: New topical treatments 44

Key question 1a: Isotretinoin vs. antibiotics: Efficacy

Research question

For patients with moderate to severe acne, which therapeutic option, isotretinoin or oral antibiotics, should be preferred in terms of effectiveness, quality of life, and (long-term) safety?

Evidence tables

Extraction at the level of systematic reviews

Head-to-head comparisons prioritized, placebo comparison only extracted if no head-to-head available

AAD

<table>
<tr><th>Reference</th><th>Characteristics</th><th>Results</th><th>Comment</th></tr>
<tr>
<td>Reynolds RV. Guidelines of care for the management of acne vulgaris. J Am Acad Dermatol. 2024 May;90(5):1006.e1-1006.e30. doi: 10.1016/j.jaad.2023.12.017. Epub 2024 Jan 30. PMID: 38300170. https://pubmed.ncbi.nlm.nih.gov/38300170/</td>
<td>SEARCH: MEDLINE and Embase from 2014 to 05 June 2021, updated periodically until 10 May 2022.
Type of study: RCTs
Intervention: topical therapies, systemic antibiotics, hormonal agents, isotretinoin, physical modalities, complementary / alternative therapies, diet
Population: adults, adolescents and preadolescents (≥9 years) with acne vulgaris</td>
<td>1) Doxycycline vs. Azitromycin
<table>
<tr><th>Outcome</th><th>Studies</th><th>Doxy</th><th>Azi</th><th>Estimate</th><th>Certainty</th></tr>
<tr><td>Physician global assessment (GA) ≥12 wks (assessed with clear or almost clear)</td><td>1 RCT (Maleszka R 2011)</td><td>55/115 (47.8 %)</td><td>47/109 (43.1 %)</td><td>RR 1.11 (95% CI 0.83; 1.48)</td><td>moderate</td></tr>
<tr><td>TLC ≥12 wks (change from baseline)</td><td>1 RCT (Maleszka R 2011)</td><td>N=115 (-57±22)</td><td>N=109 (-51±21)</td><td>MD 6 lower (95% CI 11.63 lower; 0.37 lower)</td><td>moderate</td></tr>
<tr><td>TLC 12 wks (assessed with 80-100% reduction)</td><td>2 RCTs (Babaeinejad S 2011; Ullah G 2014)</td><td>174/243 (71.6%)</td><td>91/243 (37.4%)</td><td>RR 1.68 (95% CI 0.56; 5.0)</td><td>low</td></tr>
<tr><td>ILC 12 wks (assessed with ≥75% reduction)</td><td>1 RCT (Maleszka R 2011)</td><td>68/115 (59.1%)</td><td>56/109 (51.4%)</td><td>RR 1.15 (95% CI 0.91; 1.46)</td><td>moderate</td></tr>
<tr><td>ILC 12 wks (assessed with 80-</td><td>1 RCT (Kus S 2005)</td><td>15/24 (62.5 %)</td><td>11/21 (52.4 %)</td><td>RR 1.19 (95% CI 0.71; 1.99)</td><td>moderate</td></tr>
</table>
</td>
<td>AMSTAR-II: critically low

additional references for comparison 2:
Leyden JJ 1996 (Reduction of P. acnes)
Margolis DJ 2021 (IBD)</td>
</tr>
</table>

Reference	Characteristics	Results	Comment

100% reduction)					
Patient GA at 12 wks	1 RCT (Kus S 2005)	**Narrative synthesis:** see supplement of publication page 276/700<br>Group 1: azithromycin 500 mg: 1st month, 3x/week, 2nd month 2x/week, 3rd month 1x/week.<br>Group 2: doxycycline 100 mg 2x/day for the 1st month, and 1x/day for following 2 months.<br>Results:<br>- no significant differences in terms of percentage reduction of […] patients' own assessment			moderate
Withdrawal due to AE	1 RCT (Kus S 2005)	0/24 (0%)	2/21 (9.5%)	RR 0.18 (95% CI 0.01; 3.47)	moderate
Any AE	1 (Babaeinejad SE 2011)	0/50 (0%)	4/50 (8.0%)	RR 0.11 (95% CI 0.01; 2.01)	moderate

2) Minocycline vs Doxycycline

Outcome	Studies	Mino	Doxy	Estimate	Certainty
Physician's global assessment (GA) 12 wks (assessed with 4-point scale)	1 RCT (Olafsson JH 1989)	27/31 (87.1%)	28/33 (84.8%)	RR 1.03 (95% CI 0.84; 1.25)	moderate
Patient's global assessment (GA) 12 wks (assessed with 4-point scale)	1 RCT (Olafsson JH 1989)	28/31 (90.3%)	28/33 (84.8%)	RR 1.06 (95% CI 0.89; 1.28)	moderate
Withdrawal due to AE	1 RCT (Olafsson JH 1989)	3/39 (7.7%)	2/40 (5.0 %)	RR 1.54 (95% CI 0.27; 8.71)	moderate

Reference	Characteristics	Results	Comment
		(see nested tables below)	

Any AE	1 RCT (Olafsson JH 1989)	3/39 (7.7%)	2/40 (5.0 %)	RR 1.54 (95% CI 0.27; 8.71)	moderate

3) Doxycycline vs. Erythromycin

Outcome	Studies	Doxy	Ery	Estimate	Certainty
Physician global assessment (GA) 6 wks (change from baseline; assessed with 6-point scale)	1 RCT (Cao T 2018)	15 (1.1 ± 0.9)	14 (0.8 ± 0.1)	MD 0.3 higher (95% CI 0.16 lower; 0.76 higher)	low

4) Isotretinoin traditional dose vs. intermittent dose

Outcome	Studies	QD	intermittent	Estimate	Certainty
Physician's global assessment (GAGS) 24 wks	1 RCT (Lee JW 2011)	16	17	MD 1.75 lower (95% CI 3.38 lower; 0.12 lower)	moderate
Physician's global assessment (FDA global grade, 4-point scale) 6 months	1 RCT (Akman A 2007)	Group 1: 0.5 mg/kg/d for the first 10 days of each month for 6 months (n= 22)<br>Group 2: 0.5 mg/kg/d every day for 1 month, then 0.5 mg/kg/d for the first 10 days of each month for 5 months (n= 19)<br>Group 3: 0.5 mg/kg/d every day for 6 months (n= 19)<br>**Results:**<br>- FDA global grade in each group: significantly decreased compared with baseline at the end of treatment (P<0.001).<br>- Differences were also significant in each group at the end of follow-up period (P<0.001)<br>- no statistically significant differences between the 3 treatment groups according to repeated measure of ANOVA (P = 0.554)<br>- However, when evaluated according to FDA global grade: statistically significant difference			low

Reference	Characteristics	Results	Comment
		(see nested table below)	

		between groups 1 and 3 at the end of the 12-month follow-up period (P=0.002).<br>- difference between groups 2 and 3 was close to statistical significance (P=0.053).			
Total acne load, change from baseline at 16 weeks	1 RCT (Agarwal US 2011)	120 patients with acne<br>- Group A: isotretinoin 1 mg/kg/day,<br>- Group B: 1 mg/kg/alternate day,<br>- Group C: 1 mg/kg/day for 1 week/4 weeks<br>- Group D: 20 mg every alternate day<br>- Treatment for 16 weeks<br>- Follow up: 8 further weeks without treatment<br>**Results:**<br>mean acne score reduction (range) wk 16:<br>- Group A: 7.74 (0-27)<br>- Group B: 20 (1-53)<br>- Group C: 43.5 (1-169)<br>- Group D: 26.24 (4-79)			moderate
IL at 24 weeks	1 RCT (Lee JW 2011)	16	17	MD 3.87 lower (95% CI 5.57 lower; 2.17 lower)	moderate
NIL at 24 weeks	1 RCT (Lee JW 2011)	16	17	MD 4.53 lower (95% CI 6.89 lower; 2.17 lower)	moderate
Patient global assessment (GA) at 24 weeks	1 RCT (Lee JW 2011)	16	17	MD 0.25 lower (95% CI 0.72 lower; 0.22 higher)	low
Recurrence at 1 year after completion of 6-month treatment	1 RCT (Lee JW 2011)	2/16 (12.5%)	9/17 (52.9%)	RR 0.24 (95%CI 0.06; 0.93)	low

Reference	Characteristics	Results	Comment

Withdrawal due to AE	1 RCT (Lee JW 2011)	2/30 (6.7%)	0/30 (0%)	RR 5.00 (95%CI 0.25; 99.95)	moderate
Elevated LFT	3 RCTs (Lee JW 2011; Akman A 2007; Agrawal US 2011)	2/57 (3.5%)	0/115 (0%)	RR 5.25 (95%CI 0.56; 49.26); I^2= 0%	moderate
Elevated trigylceride	3 RCTs (Lee JW 2011; Akman A 2007; Agrawal US 2011)	3/57 (5.3%)	1/115 (0.9%)	RR 4.83 (95%CI 0.73; 32.02); I^2= 0%	moderate

5) Isotretinoin traditional dosage (0.5-1.0mg/kg/day) vs. low dosage (<0.5mg/kg/day)

Outcome	**Studies**	**Traditional dosage**	**Low dosage**	**Estimate**	**Certainty**
Physician global assessment (GAGS) 24 wks	1 RCT (Lee JW 2011)	16	17	MD 0.18 lower (1.78 lower; 1.42 higher)	moderate
Acne grade at week 16 (11 point-scale; face, back, chest)	1 RCT (King 1982)	13-cis-retinoic acid daily for 16 weeks:<br>- Group 1: 1.0 mg/kg/bw<br>- Group 2: 0.5 mg/kg/bw<br>- Group 3: 0.1 mg/kg/bw<br>**Results:**<br>- gradual reduction in acne grade over 16 weeks treatment with a final improvement of 70%.<br>- no difference in effectiveness between doses			

Reference	Characteristics	Results						Comment
		TLC, face	1 RCT (Strauss JS 1984)	Isotretinoin for 20 weeks<br>- Group 1: 1.0 mg/kg/bw<br>- Group 2: 0.5 mg/kg/bw<br>- Group 3: 0.1 mg/kg/bw<br>**Results:**<br>- Two-sided comparison* between dosage levels disclosed significance (p ≤ 0.05) for the face on comparison of 0.1 and 1.0 mg/kg/day only at 4 and 8 weeks' posttherapy and on comparison of 0.5 and 1.0 mg/kg/day only at 8 weeks' posttherapy.<br>*Comparison of the lesion counts at any observation period between different dosages.			moderate	
		IL at 24 weeks	1 RCT (Lee JW 2011)	16	17	MD 0.15 lower (1.51 lower; 1.21 higher)	moderate	
		NIL at 24 weeks	1 RCT (Lee JW 2011)	16	17	MD 0.83 lower (3.24 lower; 1.58 higher)	moderate	
		Patient global assessment at 24 weeks	1 RCT (Lee JW 2011)	16	17	MD 0.7 lower (1.13 lower; 0.27 higher)	low	
		Recurrence at 1 year after completion of 6-month treatment	1 RCT (Lee JW 2011)	2/16 (12.5%)	3/17 (17.6%)	RR 0.71 (95%CI 0.14; 3.70)	low	
		Withdrawal due to AE	1 RCT (Lee JW 2011)	2/30 (6.7%)	0/30 (0%)	RR 5.00 (95%CI 0.25; 99.95)	moderate	
		Different AE, including laboratory marker	See supplementary material v3; pages 397 ff./700					

6) Isotretinoin 20mg QD vs. 20mg EOD for 24 weeks

Outcome	Studies	Isotretinoin 20mg QD	20mg EOD for 24 weeks	Estimate	Certainty
Total acne load at 6 months	1 RCT (Dhaked DR 2016)	118	116	MD 2.08 lower (3.51 lower; 0.65 lower)	low
LC, > 90% reduction at 24 weeks	1 RCT (Dhaked DR 2016)	116/118 (98.3%)	109/116 (94.0%)	RR 1.05 (95%CI 0.99; 1.10)	low
Different AE, including laboratory marker	See supplementary material v3; pages 402 f. /700				

7) Isotretinoin 0.5-1.0mg/kg/day once daily vs. 0.5-1.0mg/kg/day divided as twice daily

Outcome	Studies	0.5 - 1.0 mg/ kg/d once daily	0.5 - 1.0 mg/ kg/d divided as twice/d	Estimate	Certainty
Global acne score at 20 weeks (median treatment duration: 22 weeks)	1 (Ahmad HM 2015)	- for pre- to posttreatment comparison both groups showed statistically significant clinical improvement (GAS group 1: 34 → 0; GAS group 2: 31 → 0)<br>- difference of posttreatment results of the two groups was not statistically significant (p=0.8)			low
Different AE, including laboratory marker	See supplementary material v3; pages 404 ff. /700				

8) Isotretinoin vs. Azithromycin

Reference	Characteristics	Results	Comment

Outcome	Studies	Isotretinoin	Azi	Estimate	Certainty
TLC 100% reduction at the end of treatment	1 RCT (Wahab MA 2008)	24/30 (80.0%)	6/30 (20.0%)	RR 4.00 (95%CI 1.91; 8.36)	low
TLC 75-99% reduction at the end of treatment	1 RCT (Wahab MA 2008)	5/30 (16.7%)	9/30 (30.0%)	RR 0.56 (95%CI 0.21; 1.46)	low
Recurrence during the post treatment period	1 RCT (Wahab MA 2008)	5/30 (16.7%)	10/30 (33.3%)	RR 0.50 (95%CI 0.19; 1.29)	low
Different AE, including laboratory marker	See supplementary material v3; pages 408 f. /700				

9) Isotretinoin vs. Minocycline

Outcome	Studies	Isotretinoin (1 mg/ kg/ d for 10 wks, then 0.5 mg/ kg/ d for 10wks)	Mino (100 mg/ d for 10 wks, then 50 mg/ d for 10wks)	Estimate	Certainty
ILC	1 RCT (Pigatto PD 1986)	Results:<br>- pts. with Iso improved while acne of mino-treated group remained unchanged, statistically significant different number of cysts at weeks 20 (p < 0.02)			low
Different AE, including laboratory marker	See supplementary material v3; pages 408 f. /700				

Reference	Characteristics	Results	Comment
		**10) Isotretinoin + topical agents vs. isotretinoin**	

Outcome	Studies	Iso + local clinda + adapalene	Isotretinoin	Estimate	Certainty
Physician global assessment, clear, at 6 months	1 RCT (Dhir R 2008)	13/30 (43.3%)	12/30 (40.0%)	RR 1.08 (95%CI 0.59; 1.97)	low
Physician global assessment, ≥ 75% reduction, at 6 months	1 RCT (Dhir R 2008)	22/30 (73.3%)	19/30 (63.3%)	RR 1.16 (95%CI 0.82; 1.64)	low
Withdrawal due to AE	1 RCT (Dhir R 2008)	0/30 (0.0%)	0/30 (0.0%)	n.a.	low
Flare ups (erythema, edema, fresh papular and pustular lesions)	1 RCT (Dhir R 2008)	6/30 (20.0%)	3/30 (10.0%)	RR 2.00 (95%CI 0.55; 7.27)	low

NICE

Reference	Characteristics	Results	Comment
National Institute for Health and Care Excellence. Acne vulgaris: management. Available at: https://www.nice.org.uk/guidance/ng198	**Search:** Medline and EMBASE from inception to 06 May 2020<br>**Type of studies**: RCTs<br>**Population:** people with mild to moderate or moderate to severe acne vulgaris<br>**Intervention:** topical treatments, oral antibiotics, oral hormonal treatments, oral isotretinoin, physical treatments<br>**Outcomes:** efficacy, safety	**Tetracycline vs. Minocycline**<br>- 1 study included (Khanna 1993) https://ijdvl.com/treatment-of-acne-vulgaris-with-oral-tetracyclines/)	**AMSTAR-II:** low

	Tetracycline 500 mg 2x/d	Minocycline 50 mg 2x/d
**Baseline**		
n	21	23
Initial ALS	90.4	92.5
**Post treatment**		
ALS at 12 weeks (p > 0.05)	24	29.4
**Acne lesion score (ALS) at 12 weeks**		
Excellent (> 75% reduction)	5 (23.8%)	6 (26.1%)

Reference	Characteristics	Results			Comment
		Good (50 - 74% reduction)	10 (47.6%)	12 (43.5%)	
		Fair (25 - 49% reduction)	0	1 (4.3%)	
		Poor (< 25% reduction)	0	0	
		Worse	0	0	
		**RoB 2.0 (evaluated by NICE): high**			

Extraction at the level of individual studies following the search period of the AAD guidelines

Reference	Study type	Characteristics	Intervention / Comparison	N	Results		
**Doxycycline vs. azithromycin**							
Nagar C. COMPARISON OF ORAL AZITHROMYCIN PULSE THERAPY VERSUS DAILY DOXYCYCLINE IN THE TREATMENT OF ACNE VULGARIS. Journal article. International journal of academic medicine and pharmacy. 2024;6(1):939-943. https://www.cochranelibrary.com/central/doi/10.1002/central/CN-02664571/full#0	RCT, open label<br><br>RoB 2.0 (overall): High risk of bias for efficacy and safety	**Population:**<br>acne vulgaris<br>> 12 years<br>**Study duration:** 12 weeks<br>**Funding:** encouragement and support from Varun Arjun Medical College & Rohilkhand Hospital, Uttar Pradesh	Azithromycin 500mg for 3 consecutive days in a week for 8 weeks (Group A)	40		**Group A**	**Group B**
					**Baseline**		
					Female	n= 44 (55%)	
					Mean age (years)	20.33 ± 5.16	20.30 ± 4.09
					Baseline Acne score	78.87 ± 29.14	73.88 ± 23.33
					**Post treatment**		
			Doxycycline 100mg daily for 8 weeks (Group B)	40	4 weeks post treatment follow up	8.75 ± 7.34	17.40 ± 4.21
					no response	3 (7.5%)	6 (15%)
					mild response	4 (10%)	11 (27.5%)
					moderate response	8 (20%)	7 (17.5%)
					good response	20 (50%)	13 (32.5%)
					excellent response	5 (12.5%)	3 (7.5%)
					Gastrointestinal side effects	20%	46%
**Isotretinoin vs. azithromycin**							
Rajar UDM. Comparing the effects of isotretinoin 20 mg versus azithromycin 500 mg in the management of acne vulgaris: a randomized clinical trial. Journal	RCT, no information on blinding<br><br>RoB 2.0 (overall): High risk of	**Population:**<br>Patients with acne vulgaris<br>Age: 20–30 years<br>No history of topical medication or systemic acne medication within previous 4<br>**Duration:** 12 weeks	Isotretinoin 20 mg/day + topical clindamycin lotion daily for 3 months	40		**Isotretinoin**	**Azithromycin**
					**Baseline**		
					Female	28 (50.9%)	27 (49.09%)
					Mean age (years)	24.56 ± 1.29	24.18 ± 2.01
					Acne severity index (MD ± SD)	3.1 ± 0.77	3.52 ± 0.5
					**Post treatment**		

<table>
<tr><th>Reference</th><th>Study type</th><th>Characteristics</th><th>Intervention / Comparison</th><th>N</th><th>Results</th></tr>
<tr><td>article. International journal of dermatology. 2023;62(8):1082-1087. doi:10.1111/ijd.16615</td><td>bias for efficacy</td><td>Funding: none</td><td>Azithromycin 500 mg/day, 10-day cycle (followed by a 20d rest) + topical clindamycin lotion daily for 3 months</td><td>40</td><td><table>
<tr><td>Acne severity index (MD ± SD)</td><td>1.6 ± 0.54</td><td>2.22 ± 0.69</td></tr>
<tr><td>Difference in acne severity grading scale compared to baseline (MD ± SD)</td><td>-1.5 ± 0.75</td><td>-1.3 ± 0.93</td></tr>
<tr><td>Between group result</td><td colspan="2">Between group MD: 0.2 ± 0.85 favored isotretinoin over azithromycin, but the difference was statistically non-significant (P = 0.29).</td></tr>
</table></td></tr>
<tr><td colspan="6">Isotretinoin (alternate days) vs. daily Isotretinoin</td></tr>
<tr><td rowspan="2">Dawood M. Efficacy of Low Dose Oral Isotretinoin on Alternate Day Vs Pulses Regimen for Acne Vulgaris Treatment: RCT. Journal article. Pakistan journal of medical and health sciences. 2022;16(4):116-117. doi:10.53350/pjmhs221 64116</td><td rowspan="2">RCT, no information on blinding

RoB 2.0 (overall): High risk of bias for efficacy</td><td rowspan="2">Population:
Patients with severe facial acne vulgaris
Study duration: 24 weeks
Funding: none</td><td>isotretinoin 1mg/kg on alternate days (group A)

nota bene: treatment duration not clearly reported</td><td>127</td><td rowspan="2"><table>
<tr><td></td><td>Group A</td><td>Group B</td></tr>
<tr><td colspan="3">Baseline</td></tr>
<tr><td>Female</td><td>56 (44.1%)</td><td>50 (39.37%)</td></tr>
<tr><td>Mean age (years)</td><td>17.86 ± 2.910</td><td>18.20 ± 2.592</td></tr>
<tr><td>Initial Acne load, mean (range)</td><td>114.6 (32-244)</td><td>110.4 (40-228)</td></tr>
<tr><td colspan="3">Post treatment</td></tr>
<tr><td>Acne load at 12 weeks, mean (range)</td><td>20.55 (05-25)</td><td>43.74 (11-75)</td></tr>
<tr><td>Acne load at 24 weeks, mean (range)</td><td>5.24 (0-12)</td><td>22.33 (0-43)</td></tr>
<tr><td>Between group result at 12 and 24 weeks</td><td colspan="2">Chi square Test: <0.001</td></tr>
</table></td></tr>
<tr><td>isotretinoin 1mg/kg/day for one week/four weeks (group B)

nota bene: treatment duration not clearly reported</td><td>127</td></tr>
<tr><td colspan="6">Low dose isotretinoin vs. standard treatment protocol</td></tr>
<tr><td>Jabbar A. Analyzing the Effects of Low-Dose Isotretinoin on Acne Vulgaris Against the Standard Treatment Protocol. Journal article. Pakistan journal of medical and health sciences.</td><td>RCT, no information on blinding

RoB 2.0 (overall): High risk of bias for</td><td>Population:
Patients aged 16-45 years severe acne vulgaris
Duration: 12 weeks
Funding: not reported</td><td>Oral isotretinoin 20 mg/day (group I) for 12 weeks</td><td>95</td><td><table>
<tr><td></td><td>Group I</td><td>Group II</td></tr>
<tr><td colspan="3">Baseline</td></tr>
<tr><td>Female</td><td colspan="2">110 (57.9%)</td></tr>
<tr><td>Mean age (years)</td><td>24.16 ± 10.52</td><td>25.8 ± 9.87</td></tr>
<tr><td>Mean weight (kg)</td><td>67.8 ± 32.80</td><td>68.3 ± 13.67</td></tr>
<tr><td>Mean GAGS score</td><td>25.9 ± 5.25</td><td>25.3 ± 5.36</td></tr>
</table></td></tr>
</table>

Reference	Study type	Characteristics	Intervention / Comparison	N	Results		
2022;16(12):554-556. doi:10.53350/pjmhs20221612554	efficacy and safety		Oral isotretinoin 80 mg twice daily (group II) for 12 weeks	95	Mean Duration of disease (years)	1.0 ± 3.7	1.6 ± 0.44
					**Post treatment**		
					Number of participants with efficacy (**Nota bene**: no information how efficacy was evaluated)	73 (76.8%)	50 (52.6%)
					Between group result at 12 weeks	P value not extracted as statistical method not reported	
					Complications	28 (29.5%)	46 (48.4%)

Risk of bias appraisal

Reference	Outcome	Judgement for "randomization process"	Judgement for "deviations from the intended interventions (assignment)"	Judgement for "missing outcome data"	Judgement for "measurement of the outcome"	Judgement for "selection of the reported result"	Overall risk of bias judgement
Nagar C., et al. International journal of academic medicine and pharmacy, 2024, 6(1), 939-943.	Total acne score	some concerns	high risk of bias	low risk of bias	high risk of bias	some concerns	**high risk of bias**
	side effects			low risk of bias	high risk of bias		**high risk of bias**
Rajar UDM, et al. International journal of dermatology, 2023, 62(8), 1082-1087	acne severity index (1-4)	some concerns	some concerns	low risk of bias	low risk of bias	some concerns	**high risk of bias**
Dawood M., et al. Pakistan journal of medical and health sciences, 2022, 16(4), 116-117 2022 Issue 05	Total acne load at 12 weeks and at 24 weeks	some concerns	some concerns	low risk of bias	high risk of bias	some concerns	**high risk of bias**
Jabbar A., et al. Analyzing the Effects of Low-Dose Isotretinoin on Acne Vulgaris Against the Standard Treatment Protocol. Journal article. Pakistan journal of medical and health sciences. 2022;16(12):554-556.	Efficacy	some concerns	some concerns	low risk of bias	high risk of bias	some concerns	**high risk of bias**
	Complications			low risk of bias	high risk of bias	some concerns	**high risk of bias**

Key question 1b: Safety of long term antibiotic use

Research question

What safety signals have been observed with long-term use of azithromycin, erythromycin, tetracycline, doxycycline, minocycline, lymecycline and sarecycline?

PICO-Question and screening criteria

	Inclusion	Exclusion
**Population**	Male and female patients with any disease	
**Intervention vs. Comparison**	Long term use* of antibiotics<br>• Erythromycin<br>• Tetracycline<br>• Doxycycline<br>• Minocycline<br>• Lymecycline<br>• Azithromycin<br>• Sarecycline<br>* long term defined as > 12 weeks	Other antibiotics
**Outcome**	Safety, as reported<br>Resistance<br>Effects on microbiome	
**Study design**	Systematic reviews	
**Time frame**	Stepwise and retroactive (until 2021)	

Evidence tables

Extraction at the level of systematic reviews

Reference	Characteristics	Results	Comment
Waitayangkoon P., et al. Long-Term Safety Profiles of Macrolides and Tetracyclines: A Systematic Review and Meta-Analysis. Journal of Clinical	**Search:** Medline and EMBASE from inception to October 2022<br>**Type of studies**: RCTs<br>**Population:** people with any chronic condition<br>**Intervention:**<br>- macrolides, tetracyclines compared with placebo<br>- treatment duration > 6 months<br>**Outcomes:** long-term safety information.	**Baseline information:**<br>- 52 RCTs included,<br>- 3151 participants on doxycycline<br>- 2519 participants on minocycline<br>- 3049 participants on azithromycin<br>- 262 participants on erythromycin<br>- […]	

Reference	Characteristics	Results	Comment
Pharmacology. 2024.64(2):164-177. https://pubmed.ncbi.nlm.nih.gov/37751595	**AMSTAR-II:** critically low quality (Heterogeneity not addressed)	**SAE and withdrawal due to AE**<br>**Results:** for details see table 3 in publication	

drug	Number of trials	Treatment arm n (%)	Placebo arm n (%)	RR (95% CI)
**SAE**				
Doxycycline	20	116/1611 (7.2)	121/1600 (7.6)	0.95 (0.75-1.21)
Minocycyline	14	301/1346 (22.4)	242/1173 (20.6)	1.08 (0.93-1.25)
Azithromycin	12	225/1517 (14.8)	208/1532 (13.6)	1.09 (0.92-1.30)
Clarithromycin	2	0	0	NA
Erythromycin	3	0	0	NA
Roxythromycin	1	0	0	NA
**Withdrawal due to AE**				
Doxycycline	20	88/1611 (5.5)	31/1523 (2.0)	**2.82 (1.88-4.22)**
Minocycyline	14	110/1346 (8.2)	65/1173 (5.5)	**1.48 (1.09-1.98)**
Azithromycin	12	97/1517 (6.3)	64/1532 (4.2)	**1.53 (1.13-2.08)**
Clarithromycin	2	52/382 (13.6)	42/381 (11.0)	1.25 (0.84-1.81)
Erythromycin	3	8/130 (6.2)	5/132 (3.8)	1.62 (0.55-4.83)
Roxythromycin	1	1/50 (2)	1/50 (2)	1.00 (0.06-15.55)

AE with statistically significant differences within comparisons

Doxycycline: for details see table 4 in publication

Adverse effect	Number of trials	Treatment arm n/N	Placebo arm n/N	RR (95% CI)
GI disorders	9	434/1004	276/985	1.54 (1.36-1.74)
Nausea/ vomiting	5	180/619	70/610	2.53 (1.97-3.26)
Diarrhea	5	90/607	70/728	1.54 (1.15-2.07)
Dermatologic disorders	6	234/650	95/643	2.43 (1.97-3.01)
Photosensitivity	6	148/868	35/856	4.17 (2.92-5.95)
Rash/erythema	3	86/494	60/490	1.42 (1.04-1.93)

Minocycline: for details see table 5 in publication

<table>
<tr><th>Reference</th><th>Characteristics</th><th>Results</th><th>Comment</th></tr>
<tr><td></td><td></td><td>
<table>
<tr><th>Adverse effect</th><th>Number of trials</th><th>Treatment arm n/N</th><th>Placebo arm n/N</th><th>RR (95% CI)</th></tr>
<tr><td>GI disorders</td><td>11</td><td>788/1235</td><td>432/1058</td><td>1.56 (1.43-1.69)</td></tr>
<tr><td>Nausea</td><td>6</td><td>98/507</td><td>50/515</td><td>1.99 (1.45-2.73)</td></tr>
<tr><td>Dermatologic disorders</td><td>8</td><td>339/1032</td><td>193/852</td><td>1.45 (1.25-1.69)</td></tr>
<tr><td>Rash</td><td>5</td><td>24/430</td><td>10/436</td><td>2.43 (1.18-5.03)</td></tr>
<tr><td>Skin/tooth discoloration</td><td>2</td><td>24/138</td><td>4/137</td><td>5.95 (2.12-16.71)</td></tr>
<tr><td>Neurological disorders</td><td>10</td><td>479/1212</td><td>315/1035</td><td>1.29 (1.16-1.46)</td></tr>
<tr><td>Dizziness</td><td>6</td><td>64/507</td><td>37/515</td><td>1.76 (1.19-2.58)</td></tr>
</table>
Azithromycin: for details see table 6 in publication
<table>
<tr><th>Adverse effect</th><th>Number of trials</th><th>Treatment arm n/N</th><th>Placebo arm n/N</th><th>RR (95% CI)</th></tr>
<tr><td>GI disorders</td><td>11</td><td>384/1428</td><td>275/1441</td><td>1.41 (1.23-1.61)</td></tr>
<tr><td>Nausea</td><td>6</td><td>73/477</td><td>46/478</td><td>1.59 (1.12-2.25)</td></tr>
<tr><td>Diarrhea</td><td>7</td><td>129/558</td><td>65/560</td><td>1.99 (1.51-2.62)</td></tr>
<tr><td>Hearing decrement</td><td>2</td><td>147/615</td><td>114/616</td><td>1.29 (1.04-1.60)</td></tr>
</table>
Erythromycin: for details see table 8 in publication
</td><td></td></tr>
<tr><td>Janjua S., et al. Prophylactic antibiotics for adults with chronic obstructive pulmonary disease: a network meta-analysis. Cochrane Database of Systematic Reviews. 2021.1:CD013198. https://pubmed.ncbi.nlm.nih.gov/33448349</td>
<td>SEARCH: Cochrane Airways Group Specialised Register of trials and clinical trials registries. on 22 January 2020.
Type of study: RCTs of ≥ 12 weeks duration
Intervention: antibiotics prophylactically compared with other antibiotics, or placebo,
Population: COPD
Outcomes: efficacy, all cause SAE, Drug resistance/Microbial sensitivity, Mortality
AMSTAR-II: high quality</td>
<td>Serious adverse events
- 9 studies included (n= 3180) in the NMA.
- Macrolides reduced odds of SAE compared with placebo (fixed effect-fixed class effect: OR 0.76, 95% CrI 0.62 to 0.93).
- probably little to no difference in effect of tetracycline + macrolide compared with placebo.
- macrolide treatment: 49 fewer people/1000 experienced SAE compared with placebo.
Information on resistance:
- only extracted for studies with a treatment duration > 12 wks
Doxycycline:
- Brill 2015 (n= 99 randomised): change in MIC of 3.74 (95% CI 1.46 to 16.19) with doxycycline (100 mg once daily) compared with placebo at 13 weeks.
- isolates from participants taking doxycycline: more likely to be resistant to doxycycline than from those taking placebo (OR 5.77, 95% CI 1.40 to 23.74; P = 0.02).
Erythromycin:</td>
<td></td></tr>
</table>

Reference	Characteristics	Results	Comment
		- He 2010 (n= 36 randomised): no significant group differences (erythromycin 250 mg, 3 times a day vs. placebo, 26 wks) in the emergence of antibiotic resistant organisms.<br>- Seemungal 2008 (n= 109 randomised): 1 participant had colonisation of S. pneumoniae resistant to erythromycin in the erythromycin (250 mg twice daily, 52 wks) treatment arm. All Haemophilus influenzae isolates (22/109) were found to be resistant to erythromycin.<br>**Azithromycin:**<br>- Albert 2011: already reported in Shim et al.<br>- Uzun 2014: (n= 92 randomised): fewer people had resistant bacteria when taking azithromycin (500 mg, 3x/week, 52 wks) compared with placebo (3 vs. 11; P = 0.036).<br>- Vermeersch 2019 (n= 301 randomised): no significant group differences between azithromycin (500 mg 1x/d for 3 days, followed by 250 mg every 2 days, 13 wks) and placebo for acquired macrolide-resistant bacteria. 1 participant in placebo group had newly acquired macrolide-resistant bacteria.<br>- Blasi 2010 (n= 22 randomised): 1 participant in the azithromycin group (500 mg daily, 3 times a week, 26 wks.) had erythromycin-resistant S. pneumoniae.<br>- Brill 2015 (n= 99 randomised), (azithromycin 250 mg, 3 times a week, 13 wks): most common organisms in sputum were S. pneumoniae and Streptococcus species. Antibtiotic resistance was increased, with a factor increase of mean inhibitory concentration of 6.23 (95% CI 1.66 to 23.35; P = 0.01) compared with placebo for sputum isolated cultures	
Mavranezouli I., et al. A systematic review and network meta-analysis of topical pharmacological, oral pharmacological, physical and combined treatments for acne vulgaris. British Journal of Dermatology. 2022.187(5):639-649. https://pubmed.ncbi.nlm.nih.gov/35789996	**OBJECTIVES:** identify best treatments for mild-to-moderate and moderate-to-severe acne [SR on NICE Guideline]<br>**Search:** up to May 2020<br>**Type of study:** RCTs<br>**Interventions:**<br>- topical pharmacological,<br>- oral pharmacological,<br>- physical and combined treatments for mild-to-moderate and moderate-to-severe acne, published<br>→ duration not reported<br>**Comparison**:<br>- each other<br>**Outcomes:**<br>- efficacy […]<br>- treatment discontinuation for any reason (reflecting acceptability)<br>- treatment discontinuation owing to side-effects (reflecting tolerability).<br>**AMSTAR-II:** moderate	**Outcome of interest:**<br>**discontinuation due to side-effects:**<br>mild-moderate acne:<br>- Tetracycline (oral) vs. Placebo: log OR 0.71 (credible interval -0.43 to 1.86); n= 489<br>- Macrolide (oral) vs. Placebo: log OR 3.43 (credible interval -0.23 to 9.42); n= 160<br>Moderate-severe acne:<br>- Tetracycline (oral) vs. Placebo: log OR 0.92 (credible interval -0.30 to 2.41); n= 1307	

Extraction at the level of studies included in systematic reviews

<u>Important note:</u> A systematic search for systematic reviews was conducted to evaluate the safety of long-term antibiotic use (defined as > 12 weeks). However, not all publications addressed this question directly. For instance, some reviews pooled different antibiotics, while others included primary studies investigating applications of ≤ 12 weeks. In such cases, the results of the systematic review were not extracted, but rather those of relevant primary studies that were included in the systematic reviews.

The cells of the table were highlighted in red if resistance or other microbiological outcomes were reported

<table>
<tr><th rowspan="2">Reference</th><th>Systematic review</th><th colspan="4">Level of included studies, relevant for the guideline question</th><th rowspan="2">Comment</th></tr>
<tr><th>Characteristics</th><th>Underlying disease</th><th>Drug exposed/ administered</th><th>N</th><th>Results</th></tr>
<tr><td>Truong R., et al. A systematic review of the impacts of oral tetracycline class antibiotics on antimicrobial resistance in normal human flora. JAC-antimicrobial Resistance. 2022.4(1):dlac009 .
https://pubmed.ncbi.nlm.nih.gov/35198979</td><td>Search: MEDLINE, EMBASE, the Cochrane Library (1940-2021) and conference proceedings (2014-21) for randomized controlled trials
Population: adults with different underlying diseases
Intervention: comparing daily oral tetracycline-class antibiotics to non-tetracycline controls. (duration not reported)
Outcomes:
- AMR to tetracyclines
- resistance to non-tetracyclines.
AMSTAR-II: low quality</td><td>Acne</td><td>100 mg Minocycline once daily (up to 18 wks)

(= regimen 2)</td><td>130</td><td>Ozolins 2004 (https://pubmed.ncbi.nlm.nih.gov/15610805/)
<table><tr><th></th><th>6 wks</th><th>12 wks</th><th>18 wks</th></tr><tr><td>Number of participants reporting gastrointestinal AE</td><td>14</td><td>8</td><td>2</td></tr><tr><td>Number of participants reporting Central nervous system AE</td><td>12</td><td>5</td><td>2</td></tr><tr><td>Number of participants reporting skin AE</td><td>5</td><td>4</td><td>2</td></tr><tr><td>Mean (SD) patient-assessed summed irritation score</td><td>2.0 (2.4)</td><td>2.1 (2.1)</td><td>2.0 (2.2)</td></tr></table><u>Drop outs due to AE:</u> n= 6
<u>Resistance:</u>
- No regimen promoted an overall increase in the frequency of propionibacteria resistant to the antibiotic in the regimen and more participants lost resistant isolates than gained them.
- Details see table 5 in publication and Web table 4
Risk of bias: moderate</td><td></td></tr>
<tr><td rowspan="2">Minichino A., et al. The gut-microbiome as a target for the treatment of schizophrenia: A systematic review and meta-analysis of randomised controlled trials of add-on strategies. Schizophrenia</td><td rowspan="2">Search: Web of Science, EMBASE, PsycINFO, Cochrane CENTRAL (inception to August 2019)
Population: patients with schizophrenia (every age)
Intervention: add-on treatment with antibiotics, antimicrobics, pre/probiotics, fecal transplant
Outcomes: Efficacy and Acceptability of treatment</td><td rowspan="2">Schizophrenia</td><td>Minocycline (200 mg/d)
24 wks

or placebo</td><td>54</td><td>Levkovitz 2010 (https://pubmed.ncbi.nlm.nih.gov/19895780/)
Number of patients with side effects:
minocycline group:
- indigestion: n= 2
- pigmentation: n= 2
- suicide attempt: n= 1
placebo group:
- no adverse events occurred
Risk of bias: low</td><td></td></tr>
<tr><td>Minocycline (200 mg/d)</td><td>144</td><td>Chaudry 2012 (https://pubmed.ncbi.nlm.nih.gov/22526685/)
Risk of bias: low</td><td>Included in Waitayangkoon</td></tr>
</table>

<table>
<tr><th rowspan="2">Reference</th><th>Systematic review</th><th colspan="4">Level of included studies, relevant for the guideline question</th><th rowspan="2">Comment</th></tr>
<tr><th>Characteristics</th><th>Underlying disease</th><th>Drug exposed/ administered</th><th>N</th><th>Results</th></tr>
<tr><td rowspan="3">Research. 2021.234:1-13.
https://pubmed.ncbi.nlm.nih.gov/32295752</td><td rowspan="3">5 studies on minocycline (as add on) included
1 study on azithromycin included, without report on AE, SAE, microbiological information
AMSTAR-II: low quality</td><td rowspan="3"></td><td>up to 12 months

or placebo</td><td></td><td><table>
<tr><th>Number of patients with side effects:</th><th>Minocycline</th><th>Placebo</th></tr>
<tr><td>nausea</td><td>n= 12</td><td>n= 11</td></tr>
<tr><td>headache</td><td>n= 9</td><td>n= 17</td></tr>
<tr><td>anorexia</td><td>n= 7</td><td>n= 5</td></tr>
<tr><td>vomiting</td><td>n= 5</td><td>n= 5</td></tr>
<tr><td>dizziness</td><td>n= 4</td><td>n= 14</td></tr>
<tr><td>skin discoloration</td><td>n= 3</td><td>n= 3</td></tr>
<tr><td>visual disturbance</td><td>n= 3</td><td>n= 6</td></tr>
<tr><td>tooth discoloration</td><td>n= 0</td><td>n= 6</td></tr>
<tr><td>rash</td><td>n= 0</td><td>n= 4</td></tr>
<tr><td>vertigo</td><td>n= 0</td><td>n= 3</td></tr>
<tr><td>oesophageal irritation</td><td>n= 0</td><td>n= 4</td></tr>
<tr><td>extrapyramidal (Parkinsonian) side effects</td><td>n= 0</td><td>n= 2</td></tr>
</table></td><td></td></tr>
<tr><td>Riperidone + Minocycline (200 mg per day)
16-week

or risperidone + placebo</td><td>92</td><td>Liu 2014 (https://pubmed.ncbi.nlm.nih.gov/24503176/)
incidence of adverse events:
- minocycline 61.9% vs. placebo: 71.4%
- χ2 = 0.875, df = 1, p = 0.355
- no significant differences in the frequency and types of adverse events reported between two groups (Extrapyramidal symptoms; Dry mouth, constipation, urinary hesitancy; Dizziness; Nausea; Weight gain >7%)
- for details see table 4 in publication
serious adverse events: none observed.
Risk of bias: low</td><td></td></tr>
<tr><td>Minocycline 200 mg/day for 2 weeks, then 300 mg/day for remainder of 12-month

Or Placebo</td><td>207</td><td>Deakin 2018 (https://pubmed.ncbi.nlm.nih.gov/30322824/)
SAE:
- n=11 in placebo group and n=18 in the minocycline group
→ mostly due to admissions for worsening psychiatric state (n=10 in the placebo group and n=15 in the minocycline group).
AE:
- gastrointestinal: n=12 in the placebo group, n=19 in the minocycline group
- psychiatric: n=16 in placebo group, n=8 in minocycline group
- nervous system: n=8 in the placebo group, n=12 in the minocycline group
- dermatological: n=10 in the placebo group, n=8 in the minocycline group
for further details: see supplement of publication, table S9
Risk of bias: low</td><td>Included in Waitayangkoon</td></tr>
</table>

Reference	Systematic review	Level of included studies, relevant for the guideline question				Comment
	Characteristics	Underlying disease	Drug exposed/ administered	N	Results	
			Minocycline (200 mg/day) for 16 wks.<br><br>or placebo	200	**Weiser 2019 (https://pubmed.ncbi.nlm.nih.gov/30455075/)**<br>**Adverse events:**<br>- No significant difference was found between groups in adverse events<br>- For details see table 3 in publication<br>**Risk of bias:** no Evaluation identified	
Singh S., et al. Interventions for bullous pemphigoid. Cochrane Database of Systematic Reviews. 2023.8:CD002292 https://pubmed.ncbi.nlm.nih.gov/37572360	**OBJECTIVES:** effects of treatments for bullous pemphigoid<br>**SEARCH METHODS:** to November 2021: Cochrane Skin Specialised Register, CENTRAL, MEDLINE, and Embase<br>**SELECTION CRITERIA:** RCTs of treatments for immunofluorescence-confirmed bullous pemphigoid.<br>**Outcomes:** healing of skin lesions and mortality<br>**AMSTAR-II:** high quality	Bullous pemphigoid	Doxycycline (200 mg per day)<br>for up to 12 months	140	**Williams, 2017 (https://pubmed.ncbi.nlm.nih.gov/28279484/)**<br>- safety analysis: n= 121<br>**adverse events by 52 weeks that were possibly, probably, or definitely related to study treatment** (n/N (%))<br>- Grade 3 (severe): 14/121 (11.6%)<br>- Grade 4 (life-threatening): 5/121 (4.1%)<br>- Grade 5 (death): 3/121 (2.5%)<br>**Drop outs**<br>- due to AE: n= 2<br>- due to death: n= 14<br>**Risk of bias:** low (blinding unclear)	Not extracted: Fivenson, 1994 (duration: 10 mo) reason: combination of tetracycline and Nicotinamid
Shim S. R., et al. Increased risk of hearing loss associated with macrolide use: a systematic review and meta-analysis. Scientific Reports. 2024.14(1):183. https://pubmed.ncbi.nlm.nih.gov/38167873	**Search:** PubMed, MEDLINE, Cochrane, and Embase (inception to May 2023)<br>**Outcome:** hearing loss, tinnitus, or ototoxicity<br>**Intervention/Exposure:** macrolide (azithromycin, clarithromycin, erythromycin, fidaxomicin, roxithromycin, spiramycin, and/or telithromycin)<br>**Type of study:** RCTs, observational studies (case-control, cross-section, and cohort studies)<br>**AMSTAR-II:** low	Cystic fibrosis	250 mg (weight <40 kg) or 500 mg (weight ≥40 kg) of oral Azithromycin 3 days a week for 168 days<br><br>vs. Placebo	185	Saiman 2003 (https://pubmed.ncbi.nlm.nih.gov/14519709/)<br>**Microbiology (at day 168):**<br>- **newly detected methicillin-susceptible S. aureus**: 10% fewer participants in the azithromycin group (95% CI, –19% to –3%; P=.01).<br>- **S. aureus was eradicated:** from 18% of participants in the azithromycin group and 12% of participants in the placebo group (95% CI, –16% to 5%; P=.46).<br>- emergence or eradication of multidrug resistant strains of P. aeruginosa or other potential pathogens, incl. nontuberculous mycobacteria: little difference between the 2 groups<br>- **P aeruginosa density:** decreased by 0.3 log colony forming units at day 168 in the azithromycin group and increased by 0.2 log colony forming units in the placebo group (mean difference, 0.5 log colony forming units; 95% CI, 0.4-0.6; P=.06)<br>**Adverse Events**<br>- **nausea:** 17% more participants in azithromycin group (95% CI, 5%-29%; P=.01),<br>- **diarrhea**: 15% more participants in azithromycin group (95% CI, 4%-25%; P=.009)<br>- **wheezing**: 13% more participants in azithromycin group (95% CI, 4%-23%; P=.007)<br>- **laboratory abnormalities:** no statistically significant differences in between azithromycin and placebo groups<br>- **hearing loss:** no evidence to suggest that hearing loss was more frequent in the azithromycin group (8 [18%] of 44) than in the placebo group (12 [24%] of 50; RR, 0.8; 95% CI, 0.3-1.7)	Included in Waitayangkoon

<table>
<tr><th rowspan="2">Reference</th><th>Systematic review</th><th colspan="4">Level of included studies, relevant for the guideline question</th><th rowspan="2">Comment</th></tr>
<tr><th>Characteristics</th><th>Underlying disease</th><th>Drug exposed/ administered</th><th>N</th><th>Results</th></tr>
<tr><td></td><td></td><td></td><td></td><td></td><td>- study drug discontinuation: azithromycin group because of sore feet and bruising (n=1), sinusitis (n=1), or rash and ankle pain (n=1).
Risk of bias: low</td><td></td></tr>
<tr><td></td><td></td><td>Malaria (Prophylaxis)</td><td>arm A: Azithromycin (750-mg loading dose followed by 250 mg/day) + doxycycline placebo arm D: Doxycycline (100 mg/day) + Azithromycin placebo Arm P: double placebo
For 20 wks</td><td>300</td><td>Taylor 2003 (https://pubmed.ncbi.nlm.nih.gov/12821468/)
- withdrawal due to AE: n= 8
- for details see table 1 in publication
Azithromycin vs. Doxycycline
<table>
<tr><th>AE</th><th>Azi n (%)</th><th>Doxy n (%)</th><th>RR (95% CI)</th></tr>
<tr><td>Heartburn</td><td>73 (1.92)</td><td>25 (1.17)</td><td>1.6 (1.04–2.7)</td></tr>
<tr><td>Paresthesia</td><td>78 (2.06)</td><td>5 (0.23)</td><td>8.8 (3.6–27.9)</td></tr>
<tr><td>Severe itching</td><td>19 (0.5)</td><td>2 (0.09)</td><td>5.3 (1.3–47.5)</td></tr>
<tr><td>Abdominal pain (severe)</td><td>2 (0.05)</td><td>10 (0.47)</td><td>8.85 (1.89–83.03)</td></tr>
<tr><td>Difficulty sleeping</td><td>49 (1.3)</td><td>49 (2.29)</td><td>1.77 (1.17–2.68)</td></tr>
</table>
Azithromycin vs. Placebo
<table>
<tr><th>AE</th><th>Azithromycin n (%)</th><th>Placebo n (%)</th><th>RR (95% CI)</th></tr>
<tr><td>Heartburn</td><td>73 (1.92)</td><td>2 (0.18)</td><td>10.48 (2.79–88.14)</td></tr>
<tr><td>Tinnitus</td><td>9 (0.24)</td><td>27 (2.48)</td><td>0.10 (0.04–0.21)</td></tr>
<tr><td>Paresthesia</td><td>78 (2.06)</td><td>11 (1.01)</td><td>2.04 (1.08–4.24)</td></tr>
<tr><td>Dermatological</td><td>160 (4.22)</td><td>25 (2.3)</td><td>1.84 (1.20–2.92)</td></tr>
<tr><td>Fever</td><td>11 (0.29)</td><td>15 (1.38)</td><td>0.21 (0.09–0.49)</td></tr>
<tr><td>Others</td><td>343 (9.05)</td><td>65 (5.97)</td><td>1.51 (1.16–2.01)</td></tr>
</table>
Doxycycline vs. Placebo
<table>
<tr><th>AE</th><th>Doxycycline n (%)</th><th>Placebo n (%)</th><th>RR (95% CI)</th></tr>
<tr><td>Anorexia</td><td>43 (2.0)</td><td>39 (3.58)</td><td>0.56 (0.35–0.89)</td></tr>
<tr><td>Heartburn</td><td>25 (1.17)</td><td>2 (0.18)</td><td>6.35 (1.58–55.3)</td></tr>
<tr><td>Severe abdominal pain</td><td>10 (0.47)</td><td>0 (0)</td><td>10.7 (1.14–∞)</td></tr>
<tr><td>Dizziness</td><td>77 (3.59)</td><td>19 (1.75)</td><td>2.06 (1.23–3.6)</td></tr>
<tr><td>Tinnitus</td><td>7 (0.33)</td><td>27 (2.48)</td><td>0.13 (0.05–0.31)</td></tr>
</table>
</td><td></td></tr>
</table>

<table>
<tr><th rowspan="2">Reference</th><th>Systematic review</th><th colspan="4">Level of included studies, relevant for the guideline question</th><th rowspan="2">Comment</th></tr>
<tr><th>Characteristics</th><th>Underlying disease</th><th>Drug exposed/ administered</th><th>N</th><th>Results</th></tr>
<tr><td rowspan="4"></td><td rowspan="4"></td><td></td><td></td><td></td><td>
<table>
<tr><th>AE</th><th>Doxycycline n (%)</th><th>Placebo n (%)</th><th>RR (95% CI)</th></tr>
<tr><td>Paresthesia</td><td>5 (0.23)</td><td>11 (1.01)</td><td>0.23 (0.06–0.72)</td></tr>
<tr><td>Difficulty sleeping</td><td>49 (2.29)</td><td>13 (1.2)</td><td>1.91 (1.02–3.84)</td></tr>
<tr><td>Dermatological</td><td>81 (3.78)</td><td>25 (2.3)</td><td>1.65 (1.04–2.69)</td></tr>
<tr><td>Fever</td><td>11 (0.51)</td><td>15 (1.38)</td><td>0.37 (0.15–0.87)</td></tr>
<tr><td>Others</td><td>188 (8.77)</td><td>65 (5.97)</td><td>1.47 (1.1–1.98)</td></tr>
</table>
Risk of bias: low
</td><td></td></tr>
<tr><td>coronary artery disease</td><td>600 mg of Azithromycin or placebo weekly for one year</td><td>4012</td><td>
Grayston 2005 (https://pubmed.ncbi.nlm.nih.gov/15843666/)
- SAE: n= 34 (12 in azithromycin vs. 22 in placebo)
- For details see table 4 in publication
<table>
<tr><th>AE</th><th>Azithromycin n (%)</th><th>Placebo n (%)</th><th>P value</th></tr>
<tr><td>Nausea</td><td>284</td><td>198</td><td><0.001</td></tr>
<tr><td>Abdominal pain</td><td>370</td><td>216</td><td><0.001</td></tr>
<tr><td>Diarrhea</td><td>724</td><td>446</td><td><0.001</td></tr>
<tr><td>Hearing loss</td><td>38</td><td>20</td><td>0.02</td></tr>
</table>
Risk of bias: low
</td><td></td></tr>
<tr><td>COPD</td><td>Azithromycin, 250 mg orally, once daily
up to 12 months

or Placebo</td><td>1142</td><td>
Albert 2011 (https://pubmed.ncbi.nlm.nih.gov/21864166/)
Resistance:
- 66 participants in azithromycin (12%) and 172 in placebo (31%) who had not had nasopharyngeal colonization at the time of enrollment became colonized during the course of the study (P<0.001).
- prevalence of resistance to macrolides: 52% (Azi) and 57% (Placebo) (P = 0.64)
- incidence of resistance to macrolides: 81% (Azi) and 41% (Placebo) (P<0.001)
- For further details see Appendix (of publication), Section G
(Serious) Adverse events/ withdrawal due to AE
- No significant differences in frequency of SAE or of AE leading to discontinuation of the study drug,
- audiogram-confirmed hearing decrement: 142 participants receiving azithromycin (25%) vs. 110 receiving placebo (20%) (P = 0.04).
- For further details see appendix (of publication), section F and H:
Risk of bias: low
</td><td>Included in Waitayangkoon</td></tr>
<tr><td>non-cystic-fibrosis</td><td>Azithromycin (250 mg</td><td>89</td><td>
Altenburg 2013 (https://jamanetwork.com/journals/jama/fullarticle/1672237)
Adverse events:
</td><td>Included in Waitayangkoon</td></tr>
</table>

Reference	Systematic review	Level of included studies, relevant for the guideline question				Comment
	Characteristics	Underlying disease	Drug exposed/ administered	N	Results	
		bronchiectasis	daily) or placebo for 12 months		- Diarrhea: (Azi: 9 (21%) vs. Placebo: 1 (3%); RR 8.36 (95% CI 1.10-63.15))<br>- Withdrawal due to AE: 1 patient in each group (2.3% vs 2.5%)<br>- For further AE (with statistically non-significant difference) see table 2 in publication<br>**A macrolide resistance rate:**<br>- 53 of 60 pathogens (88%) tested for sensitivity in 20 patients in the azithromycin group became macrolide resistant, compared with 29 of 112 pathogens (26%) in 22 patients in the placebo group (P< 0.001)<br>- For further details see table 2 in publication and e-results<br>**Risk of bias:** low	
		diverse	Macrolides<br>91 -180 d<br>181 - 365 d	1750	Dabekaussen 2022 (https://pubmed.ncbi.nlm.nih.gov/35862062/)<br>**Sensorineural Hearing Loss (SNHL)**<br>- participants who had SNHL had increased odds of having received a macrolide prescription compared with a penicillin prescription when all time frames from exposure were included (aOR, 1.31; 95% CI, 1.05-1.64)<br>- for further details see eTable 2 in publication<br>- significantly higher odds of macrolide exposure than penicillin exposure when diagnosis and testing occurred more than 180 days after antibiotic exposure (aOR, 1.79; 95% CI, 1.23-2.60)<br>**Quality (NOS):** good (retrospective case-control study)	Children, Adolescents, and Young Adults
Undela K., et al. Macrolides versus placebo for chronic asthma. Cochrane Database of Systematic Reviews. 2021.11:CD002997<br>https://pubmed.ncbi.nlm.nih.gov/34807989	**SEARCH**: Cochrane Airways Group Specialised Register up to March 2021.<br>**Type of study:**<br>- RCT<br>**Population:**<br>- children and adults with asthma<br>**Intervention/ Comparison:**<br>- treated with macrolides versus placebo ≥ 4 weeks.<br>**Outcomes:** […] adverse events (including mortality), withdrawal, [...]<br>**AMSTAR-II:** moderate	Asthma	azithromycin 250 mg per day for 5 days and then 1 capsule 3 times / week (26 wks)<br>or Placebo	109	Brusselle 2013 https://pubmed.ncbi.nlm.nih.gov/23291349/<br>**Safety**<br>- No significant differences were observed in the frequency of adverse events, serious adverse events or adverse events leading to discontinuation of the study drug<br>- for further details see Table S1 in supplement appendix of publication<br>**Oropharyngeal colonisation and resistance to macrolides**<br>- N= 46 participants<br>- erythromycin-resistant streptococci in the oropharynx at randomization: 11 subjects (47.8%) in AZI group and 9 subjects (39.1%) in the placebo group<br>- erythromycin-resistant oropharyngeal streptococci after 26-week: 87% of the subjects in AZI group and 35% in the placebo group were (p<0.001).<br>- proportion of streptococci resistant to erythromycin increased from 17.2% to 73.8% in AZI group and from 7.9% to 17.3% in the placebo group (p<0.001)<br>- percentage of macrolide-resistant streptococci decreased from 73.8% to 45.9% in AZI group during the 4-week washout period (p=0.104).<br>**RoB:** unclear risk of bias for blinding of outcome assessment, other domains: low risk of bias	Included in Waitayangkoon

Reference	Systematic review	Level of included studies, relevant for the guideline question				Comment
	Characteristics	Underlying disease	Drug exposed/ administered	N	Results	
			azithromycin 500 mg 3 times/week (48 wks)<br>or Placebo	420	Gibson 2017 https://pubmed.ncbi.nlm.nih.gov/28687413/<br>**Safety:**<br>- **overall SAE:** AZI in 16 (8%) patients vs. Placebo in 26 (13%) (p=0.27)<br>- **study withdrawals due to AE:** AZI (15 [7%]) vs. placebo (10 [5%]); p=0·34<br>- **drug-related adverse events:**<br>○ Diarrhoea: Azithromycin in 72 (34%) patients vs. placebo (39 [19%] (p=0.001)<br>○ no significant increase in the rate of other potentially drug-related adverse events (for further details see Table 4 in publication)<br>**Resistance**<br>- sputum culture in 180 patients: potentially pathogenic microorganisms 20 in placebo, 17 in azithromycin; p=0.58.<br>- 12 azithromycin-resistant pathogens detected after azithromycin treatment, (H. influenzae (n=4), P. aeruginosa (n=4), S. aureus (n=2), enteric gram-negative rod (n=1), and S. pneumoniae (n=1))<br>- 7 azithromycin-resistant pathogens after placebo (p=0.27 vs azithromycin) (H. influenzae (n=1), P. aeruginosa (n=4), enteric gram-negative rod (n=1), and S. pneumoniae (n=1))<br>- non-significant increase in azithromycin resistant organisms in sputum of patients treated with azithromycin compared with placebo (19/39 [49%] vs 12/42 [29%]; p=0·062).<br>- Azithromycin-resistant organisms in nose and throat swabs: similar in both groups at the end of treatment<br>- for further details see Table S8, S9, S10 in supplement appendix of publication<br>**RoB:** high risk of bias for incomplete outcome data, unclear risk of bias for selective reporting, other domains: low risk of bias	Included in Waitayangkoon
			azithromycin 250 mg (25–40 kg bodyweight) or 500 mg (> 40 kg bodyweight) once daily (30wks)<br>or Placebo	55	Strunk 2008 (https://pubmed.ncbi.nlm.nih.gov/18951618/)<br>No information on AE, SAE or resistance reported	children 6–17 years
			azithromycin 250 mg twice weekly (52 wks)		Wang 2014<br>Publication not available	

Reference	Systematic review	Level of included studies, relevant for the guideline question				Comment
	Characteristics	Underlying disease	Drug exposed/ administered	N	Results	
			or Placebo			
Nel Van Zyl K., et al. Effect of antibiotics on the human microbiome: a systematic review. International Journal of Antimicrobial Agents. 2022.59(2) https://pubmed.ncbi.nlm.nih.gov/34929293	**Search:** PubMed, Scopus and Web of Science online databases (up to 06/2021)<br>**Type of study:** mainly post hoc analyses or substudies of RCTs<br>**Underlying disease:** diverse<br>**Outcome:** microbiome of any human bodily site pre- and post-antibiotic treatment using 16S rRNA gene sequencing.<br>Increases or decreases in diversity, dissimilarity of microbial communities, and changes in taxonomic composition following antibiotic treatment were recorded as outcome measures.<br>**AMSTAR-II:** critically low<br>**Please note:** result of RoB/ Quality appraisal not reported on study level, for summary see supplement appendix of publication	Asthma	250 mg Azithromycin once daily for 5 days, then 250 mg azithromycin three times a week for 6 months<br>Or placebo	13	Lopes dos Santos Santiago 2017 (https://pubmed.ncbi.nlm.nih.gov/28486933/)<br>**Microbiological outcome:**<br>- overall composition of the oropharyngeal microbiome in patients with severe asthma is comparable to that of the healthy population<br>- Long term treatment (6 months) with azithromycin increased the species Streptococcus salivarius approximately 5-fold and decreased the species Leptotrichia wadei approximately 5-fold.<br>- significant decrease of L. buccalis/L. hofstadtii and of Fusobacterium nucleatum.<br>- 4/ 8 patients regained their initial microbial composition within one month after cessation of treatment.	Substudy to Brusselle et al. 2013
		Asthma	500 mg Azithromycin 3 times per week for 48 weeks<br><br>Or Placebo	61	Taylor 2019 (https://pubmed.ncbi.nlm.nih.gov/30875247/)<br>**Microbiological outcome:**<br>- Azithromycin did not affect bacterial load<br>- Azithromycin Therapy Reduces Sputum Phylogenetic Diversity<br>- Azithromycin did not significantly affect levels of Streptococcus pneumoniae, Staphylococcus aureus, Pseudomonas aeruginosa, or Moraxella catarrhalis.<br>- Azithromycin Therapy Reduces H. influenzae Load<br>- Azithromycin Therapy Increases Carriage of Antibiotic Resistance Genes<br>- Of the 89 antibiotic resistance genes detected, 5 macrolide resistance genes and 2 tetracycline resistance genes were increased significantly	Substudy to Gibson et al. 2013
		non-cystic fibrosis bronchiectasis	Erythromycin 400mg BID, 12 months<br><br>Or Placebo	86	Rogers 2014 (https://pubmed.ncbi.nlm.nih.gov/25458200/)<br>**change in microbiota composition between baseline and week 48:**<br>- significantly greater with erythromycin than with placebo (median Bray-Curtis score 0.52 [IQR 0.14–0.78] vs. 0.68 [0.46–0.93]; median difference 0.16, 95% CI 0.01–0.33; p=0.03).<br>**Subgroup analyses:**<br>- baseline airway infection dominated by P. aeruginosa:<br>>> erythromycin did not change microbiota composition significantly<br>- baseline airway infection dominated by organisms other than P. aeruginosa:<br>>> erythromycin caused a significant change in microbiota composition (p=0.03 [by analysis of similarity]) representing:<br>○ a reduced relative abundance of H influenzae (35.3% [5.5–91.6] vs. 6.7% [0.8–74.8]; median difference 12.6%, 95% CI 0.4–28.3; p=0.04; interaction p=0.02)<br>○ an increased relative abundance of P. aeruginosa (0.02% [0.00–0.33] vs. 0.13% [0.01–39.58]; median difference 6.6%, 95% CI 0.1–37.1; p=0.002; interaction p=0.45).<br>- For further details see table 2 in publication	

<table>
<tr><th rowspan="2">Reference</th><th>Systematic review</th><th colspan="4">Level of included studies, relevant for the guideline question</th><th rowspan="2">Comment</th></tr>
<tr><th>Characteristics</th><th>Underlying disease</th><th>Drug exposed/ administered</th><th>N</th><th>Results</th></tr>
<tr><td></td><td></td><td>Non-Cystic Fibrosis Bronchiectasis</td><td>Erythromycin 400mg BID, 12 months

Or Placebo</td><td>84</td><td>Choo 2018 (https://pubmed.ncbi.nlm.nih.gov/29669883/)
- relative abundance of oropharyngeal H. parainfluenzae: significant increase (P = 0.041)
- relative abundances of S. pseudopneumoniae: significant decreases (P = 0.024)
- relative abundances of A. odontolyticus: significant decreases (P = 0.027)
- number of subjects who carried erm(A), erm(B), erm(C), erm(F), mef(A/E), and msrA macrolide resistance genes: Erythromycin did not result in significant increase</td><td></td></tr>
<tr><td rowspan="2">Li K., et al. The efficacy of azithromycin to prevent exacerbation of non-cystic fibrosis bronchiectasis: a meta-analysis of randomized controlled studies. Journal Of Cardiothoracic Surgery. 2022.17(1):266. https://pubmed.ncbi.nlm.nih.gov/36221151</td><td rowspan="2">Search: PubMed, Embase, Web of science, EBSCO, and Cochrane library through July 2019
Type of studies: RCTs
Population: non-cystic fibrosis bronchiectasis.
Intervention: azithromycin versus placebo for (duration not specified)
AMSTAR-II: critically low</td><td rowspan="2">non-cystic fibrosis bronchiectasis</td><td>azithromycin (30 mg/kg) once a week for up to 24 months</td><td>89</td><td>Valery 2013 (https://pubmed.ncbi.nlm.nih.gov/24461664/)
Microbiological outcome
- carriage of azithromycin-resistant bacteria: significantly higher in children with azithromycin (19/41, 46%) than with placebo (4/37, 11%; OR 7.39 (95% CI 2.15; 25.39))
- for further details see table 4 in publication
Adverse events
- non-pulmonary infections: 71/112(63%) events in AZI vs. 132/209 (63%) events in placebo
- bronchiectasis-related events: 22/112 (20%) events in AZI vs. 48/209 (23%) events in placebo
- 63 admissions to hospital because of SAE (24 events in 11 children in AZI and 39 events in 19 children in placebo group).
o 41 were attributable to either exacerbation or an investigation related to bronchiectasis (e.g., bronchoscopy).
- for further details see table 5 in publication
Quality: Jadad-Score 4</td><td>children aged 1–8 years</td></tr>
<tr><td>500 mg azithromycin three times a week for 6 months</td><td>141</td><td>Wong 2012 (https://pubmed.ncbi.nlm.nih.gov/22901887/)
Microbiological outcome
- Macrolide resistance testing was not routinely undertaken
- 2/46 patients (4%) in AZI group developed macrolide-resistant S. pneumoniae at 6 months
- for further details see table 3 in publication
Adverse events<table><tr><th>AE</th><th>Azithromycin n/N (%)</th><th>Placebo n/N (%)</th></tr><tr><td>Any AE</td><td>59/71 (83%)</td><td>65/70 (93%)</td></tr><tr><td>Severe AE</td><td>4/71 (6%)</td><td>9/70 (13%)</td></tr><tr><td>Diarrhea</td><td>13/71 (18%)</td><td>4/70 (6%)</td></tr><tr><td>Nausea or vomiting</td><td>9/71 (13%)</td><td>5/70 (7%)</td></tr></table></td><td></td></tr>
</table>

<table>
<tr><th rowspan="2">Reference</th><th>Systematic review</th><th colspan="4">Level of included studies, relevant for the guideline question</th><th rowspan="2">Comment</th></tr>
<tr><th>Characteristics</th><th>Underlying disease</th><th>Drug exposed/ administered</th><th>N</th><th>Results</th></tr>
<tr><td></td><td></td><td></td><td></td><td></td><td>- for further details see table 4 in publication
Quality: Jadad-Score 5</td><td></td></tr>
<tr><td></td><td></td><td></td><td></td><td></td><td>Altenburg 2013</td><td>already extracted from Shim et al.</td></tr>
<tr><td rowspan="3">Sethi N. J., et al. Antibiotics for secondary prevention of coronary heart disease. Cochrane Database of Systematic Reviews. 2021.2:CD003610 .
https://pubmed.ncbi.nlm.nih.gov/33704780</td><td rowspan="3">SEARCH METHODS: CENTRAL, MEDLINE, Embase, LILACS, SCI-EXPANDED, and BIOSIS in December 2019
SELECTION CRITERIA:
- RCT
- antibiotics versus placebo or no intervention for secondary prevention of coronary heart disease (duration not specified)
- adult participants (>=18 years)
AMSTAR-II: high</td><td>Prevention of Acute Coronary Syndromes</td><td>Doxycycline (20 mg twice daily) for 6 months
Or Placebo</td><td>50</td><td>Brown 2004 (https://pubmed.ncbi.nlm.nih.gov/14962945/)
AE or resistance not addressed</td><td></td></tr>
<tr><td>coronary heart disease</td><td>100 mg doxycycline hydrochloride once daily for 4 months
or placebo</td><td>34</td><td>Sinisalo 1998 (https://pubmed.ncbi.nlm.nih.gov/9511041/)
Adverse events:
- mild diarrhea lasting for 1–2 days: 1 patient in each group
- mild photosensitive skin rash: 2 patients in the doxycycline group and one receiving placebo
Risk of bias: some concerns - low</td><td></td></tr>
<tr><td></td><td></td><td></td><td>Grayston 2005 (https://pubmed.ncbi.nlm.nih.gov/15843666/)</td><td>already extracted in Shim et al.</td></tr>
<tr><td rowspan="2">Golledge J.; T. P. Singh. Effect of blood pressure lowering drugs and antibiotics on abdominal aortic aneurysm growth: a systematic review and meta-analysis. Heart. 2021.107(18):1465-1471.
https://pubmed.ncbi.nlm.nih.gov/33199361</td><td rowspan="2">Search: Medline, Web of Science, The Cochrane Library from inception to August 2020
Type of studies: RCTs
Intervention: blood pressure-lowering medications or antibiotics
Outcomes: AAA growth and AAA-related events.
AMSTAR-II: critically low</td><td>abdominal aortic aneurysm</td><td>Azithromycin 600 mg once daily for 3 days and then 600 mg once weekly for 15 weeks or placebo</td><td>247</td><td>Karlsson 2009 (https://pubmed.ncbi.nlm.nih.gov/19563951/)
Number of patients with adverse events:
- active group: n= 13;
- 3 stopped taking the study medication: 1 due to diarrhea, 1 due to arthralgia, and 1 who had an allergic reaction which initially was thought to be caused by study medication but turned out to be a reaction to antihypertensive medication.
- control group: n= 8; all gastrointestinal
Risk of bias: some concerns - high</td><td></td></tr>
<tr><td>abdominal aortic aneurysm</td><td>Doxycycline 100mg orally BID for 2 years or placebo</td><td>161</td><td>Baxter 2020 (https://pubmed.ncbi.nlm.nih.gov/32453369/)
Frequency of Expected Adverse Events:
<table>
<tr><th>AE</th><th>Doxy (N= 129) n (%)</th><th>Placebo (N= 125) n (%)</th><th>Difference (95% CI)</th></tr>
<tr><td>Any symptom reported</td><td>121 (93.8%)</td><td>111 (88.8%)</td><td>5.0% (-1.9; 11.9%)</td></tr>
<tr><td>Visual disturbance</td><td>26 (20.2%)</td><td>43 (34.4%)</td><td>-14.2% (-25.1; -3.4 %)</td></tr>
<tr><td>Moderate to severe sunburn</td><td>38 (29.5%)</td><td>14 (11.2%)</td><td>18.3% (8.6; 28.8 %)</td></tr>
</table></td><td>Included in Waitayangkoon</td></tr>
</table>

<table>
<tr><th rowspan="2">Reference</th><th>Systematic review</th><th colspan="4">Level of included studies, relevant for the guideline question</th><th rowspan="2">Comment</th></tr>
<tr><th>Characteristics</th><th>Underlying disease</th><th>Drug exposed/ administered</th><th>N</th><th>Results</th></tr>
<tr><td rowspan="2"></td><td rowspan="2"></td><td></td><td></td><td></td><td>- for further details see eTable 3 in supplement of publication
Risk of bias: some concerns - high</td><td></td></tr>
<tr><td>abdominal aortic aneurysm</td><td>100 mg of doxycycline per day for 18 months or placebo</td><td>286</td><td>Meijer 2013 (https://pubmed.ncbi.nlm.nih.gov/24490266/)
<table>
<tr><th></th><th>Doxy (N= 144)
n (%)</th><th>Placebo (N= 142)
n (%)</th><th>Difference
(95% CI)</th></tr>
<tr><td colspan="4">SAE</td></tr>
<tr><td>Death</td><td>2 (1.4)</td><td>4 (2.8)</td><td>-1.4 (-13.2; 10.3)</td></tr>
<tr><td>Ruptures (Aneurysma)</td><td>0</td><td>2 (1.4)</td><td>-1.4 (-13.2; 10.3)</td></tr>
<tr><td>Other</td><td>3 (2.1)</td><td>2 (1.4)</td><td>0.7 (-11.1; 12.4)</td></tr>
<tr><td colspan="4">Treatment emergent AE (leading to withdrawal)</td></tr>
<tr><td>all</td><td>11 (7.6)</td><td>3 (2.1)</td><td>5.5 (-6.3; 17.1)</td></tr>
<tr><td colspan="4">Treatment emergent AE (mild and self-limiting)</td></tr>
<tr><td>all</td><td>17 (11.8)</td><td>18 (12.7)</td><td>0.9 (-10.3; 12.1)</td></tr>
</table>
- for further details see table 1 in publication appendix
Risk of bias: some concerns - high</td><td>Included in Waitayangkoon</td></tr>
</table>

Key question 2: Hormonal treatments

Research question

What is the effectiveness, quality of life and (long-term) safety of hormonal treatments for (female) patients with acne?

Evidence tables

Extraction at the level of systematic reviews

Cochrane review

<table>
<tr><th colspan="5">Extraction at the level of systematic reviews</th></tr>
<tr><th>Reference</th><th>Methodological quality</th><th>Characteristics I</th><th>Characteristics II</th><th>Comments</th></tr>
<tr><td rowspan="3">Arowojolu AO, Gallo MF, Lopez LM, Grimes DA. Combined oral contraceptive pills for treatment of acne. Cochrane Database Syst Rev. 2012 Jul 11;[7]:CD004425. https://pubmed.ncbi.nlm.nih.gov/22786490/</td><td>AMSTAR-2: low</td><td>Objective
• to examine whether any Combined oral contraceptive pills (COC) is more effective than other COCs, oral or topical anti-acne medications or placebo in the treatment of facial acne in women
Search period
• 2008-01/2012 as an update of the previous version of the review (2009) [https://pubmed.ncbi.nlm.nih.gov/19588355/]
Databases
• Medline, CENTRAL, POPLINE, LILACS, ClinicalTrials.gov, ICTRP
Population
• Women of any age with facial acne vulgaris
Intervention
• Any COC (estrogen and progestin) with or without concomitant acne treatment
Control
• COC, oral or topical acne treatment,
• no treatment or placebo</td><td>Relevant inclusion criteria
/
Relevant exclusion criteria
/
Types of studies
Randomized controlled trials
Outcomes
Effectiveness of drug treatment using at least one of the following outcomes:
1. Change in specific types of facial lesion [i.e., open or closed comedones, papules, pustules or nodules] counts from baseline to last available evaluation or the specific facial lesion counts at the last available evaluation;
2. Change in total facial lesion counts from baseline to last available evaluation or the total facial lesion counts at the last available evaluation;
3. Global assessments made by the clinician or the participant regarding improvement in skin condition [e.g. excellent, good or fair progress versus no change or worse];
4. Psychosocial function outcomes, such as quality of life and disability indices, and utility outcome [e.g. willingness to pay or accept treatment]; and
5. Early study discontinuation due to adverse events, including worsening of acne.</td><td></td></tr>
<tr><td colspan="4">Results</td></tr>
<tr><td colspan="4">Baseline characteristics
• 31 RCTs, 5 secondary articles
• Total of 12,579 participants</td></tr>
</table>

Extraction at the level of systematic reviews

- EE doses ranged from 20 µg to 50 µg; one trial used 17β-estradiol (E2) 1.5mg.
- trials included 11 types of progestin.
- Duration: 3 to 13 treatment cycles (mode=6 cycles)
- 24 comparisons:
    - COC to placebo: n= 6,
    - different COC groups: n= 17
    - COC to an antibiotic: n= 1
- support from pharmaceutical companies: n= 27
- Risk of bias regarding allocation concealment: low risk (n = 5), unclear risk (n = 25), high risk (n = 1)

Comparisons

- **COC versus placebo: 9 trials showed improvements regarding efficacy outcomes, one did not provide sufficient data for analysis**
    - Levonorgestrel (LNG) 100 µg / EE 20 µg vs. placebo:
        - Mean change in total lesion count: MD -9.98 [CI -16.51, -3.45], I^2= 0%, 2 studies, n= 572
        - Mean change in inflammatory lesion count: MD -2.95[-4.97, -0.93], I^2= 0%, 2 studies, n= 572
        - Mean change in non-inflammatory lesion count: MD -6.75 [-12.56, -0.94], I^2= 0%, 2 studies, n= 572
        - Clinician assessment of women with clear or almost clear lesions at cycle 6: Peto OR 1.56 [1.13, 2.18], I^2= 0%, 2 studies, n= 571
        - Participant self-assessment of acne lesion improvement: Peto OR 2.13 [1.47, 3.09], I^2= 0%, 2 studies, n= 572
        - Discontinuation due to non-acne AEs: treatment (9/174) vs control (6/176); Peto OR 1.54 [0.55, 4.31], 1 study, n= 350
        - Discontinuation due to lack of acne improvement: treatment (7/174) vs control (8/176); Peto OR 0.88 [0.31, 2.47], 1 study, n= 350
    - Norethindrone acetate (NA) 1 mg / EE 20-30-35 µg vs. placebo:
        - Clinician assessment of no, minimal or mild acne at cycle 6: Peto OR 1.86 [1.32, 2.62], 1 study, n= 555
        - Discontinuation due to any AE: treatment (20/297) vs. control (7/296) Peto OR 2.73 [1.26, 5.90], 1 study, n= 593
    - Norgestimate (NGM) 180-215-250 µg / EE 35 µg vs. placebo:
        - Mean change in total lesion count at cycle 6: MD -9.32 [-14.19, -4.45], I^2= 77.43%, 2 studies, n= 387
        - Mean change in inflammatory lesion count at cycle 6: -3.44 [-5.43, -1.44], I^2= 0%, 2 studies, n= 387
        - Mean change in comedone count at cycle 6: -5.81 [-9.77, -1.85], I^2= 79.36%, 2 studies, n= 387
        - Clinician global assessment of improved acne at cycle 6: Peto OR 3.86 [2.31, 6.44], I^2= 36.67%, 2 studies, n= 324
        - Participant self-assessment of improved acne at cycle 6: Peto OR 4.5 [2.37, 8.56], 1 study, n= 163
        - Discontinuation due to non-acne adverse event: treatment (18/246) vs. control (9/242), Peto OR 1.98 [0.91, 4.3], I^2= 0%, 2 studies, n= 488
        - Discontinuation due to worsening of acne: treatment (5/246) vs. control (1/242), Peto OR 3.75 [0.75, 18.71], I^2= 22.59%, 2 studies, n= 488
    - Dienogest 2 mg plus EE 30 µg vs. placebo:
        - Mean percentage change in inflammatory lesion count after cycle 6: MD -16.1 [-21.74, -10.46], 1 study, n= 768
        - Mean percentage change in total lesion count after cycle 6: MD -15.3 [-19.98, -10.62], 1 study, n= 774
        - Improvement of facial acne [clinical assessment]: Peto OR 3.87 [2.5, 5.99], 1 study, n= 780
        - Discontinuation due to adverse event: treatment (8/525) vs. control (3/264), Peto OR 1.33 [0.38, 4.67], 1 study, n= 789
        - Discontinuation due to reason other than adverse event: treatment (14/525) vs. control (16/264), Peto OR 0.4 [0.18, 0.86], 1 study, n= 789
    - Drospirenone (DRSP) 3 mg / EE 20 µg vs. placebo:
        - Mean percent change in total lesion count at cycle 6: MD 29.08 [3.13, 55.03], 1 study, n= 173
        - Mean percent change in inflammatory lesion count at cycle 6: MD 14.61 [5.18, 24.04], 1 study, n= 146
        - Mean percent change in non-inflammatory lesion count at cycle 6: MD 19.03 [5.13, 32.93], 1 study, n= 146
        - Mean percent change in papule count at cycle 6: MD 17.33 [5.6, 29.06], 1 study, n= 146
        - Mean percent change in pustule count at cycle 6: MD 1.73 [-11.48, 14.94], 1 study, n= 125*
        - Mean percent change in nodule count at cycle 6: MD 0.83 [-8.8, 10.46], 1 study, n= 62*
        - Mean percent change in open comedone count at cycle 6: MD -14.28 [-84.76, 56.2], 1 study, n= 141*

Extraction at the level of systematic reviews	
	* CAVE: Contrary to the headline, these three endpoints do not present statistically significant results<br>▪ Mean percent change in closed comedone count at cycle 6: MD 20.79 [3.57, 38.01], 1 study, n= 145<br>▪ Clear or almost clear [investigator assessment] at cycle 6: Peto OR 3.02 [1.99, 4.59], I^2= 0%, 2 studies, n= 575<br>▪ Participants classified as 'improved' at cycle 6 [Investigator assessment]: Peto OR 3.67 [1.46, 9.2], 1 study, n= 152<br>▪ Participants classified as 'improved' at cycle 6 [Participant assessment]: Peto OR 3.06 [1.06, 8.85], 1 study, n= 152<br>▪ Discontinuation due to adverse event: treatment (37/625) vs. control (24/626), Peto OR 1.57 [0.94, 2.62], I^2= 0%, 3 studies, n = 1251<br>▪ Discontinuation due reason other than adverse event: treatment (9/89) vs. control (11/90), Peto OR 0.71[0.28, 1.84], 1 study, n= 179<br>o Chlormadinone acetate (CMA) 2 mg / EE 30 µg vs. placebo:<br>▪ Responders [≥50% decrease in facial papules and pustules] at cycle 6.: Peto OR 2.31 [1.5, 3.55], 1 study, n= 377<br>▪ Discontinuation due to AEs: treatment (14/251) vs. control (1/126), Peto OR 3.49 [1.17, 10.4], 1 study, n= 377<br>• **Drospirenone (DRSP) 3 mg/EE 30 µg versus other COCs:**<br>o DRSP 3 mg / EE 30 µg vs. CPA 2 mg / EE 35 µg:<br>▪ Mean percentage change in total acne count at cycle 9: MD -2.5 [-26.96, 21.96], 1 study, n= 118<br>o DRSP 3 mg / EE 30 µg vs. LNG 150 µg / EE 30 µg:<br>▪ Discontinuation due to acne deterioration: treatment (4/282) vs. control (11/142), Peto OR 0.16 [0.05, 0.47], 1 study, n= 424<br>o DRSP 3 mg / EE 30 µg versus NGM 180-215-250 µg / EE 35 µg:<br>▪ Mean percentage change in ILC after cycle 6: MD -2.4 [-5.97, 1.17], 1 study, n= 1108<br>▪ Mean percentage change in TLC after cycle 6: MD -3.3 [-6.45, -0.15], 1 study, n= 1108, favours treatment<br>▪ Improvement of facial acne [clinical assessment]: treatment (527/551) vs. control (524/569), Peto OR 1.85 [1.14, 3.01], 1 study, n= 1120<br>▪ Improvement of facial acne [subject assessment]: treatment (512/550) vs. control (505/567), Peto OR 1.64 [1.09, 2.47], 1 study, n= 1117<br>▪ Discontinuation due to AE: treatment (18/566) vs. control (23/582), Peto OR 0.8 [0.43, 1.49], 1 study, n= 1148<br>▪ Discontinuation due to reason other than AE: treatment (17/566) vs. control (18/582), Peto OR 0.97 [0.5, 1.9], 1 study, n= 1148<br>o NOMAC 2 mg / E2 1.5 mg versus DRSP 3 mg / EE 30 µg:<br>▪ Clinician assessment of improved acne after cycle 13 [participants with acne at baseline]: treatment (248/512) vs. control (105/171), Peto OR 0.6 [0.42, 0.84], 1 study, n= 683<br>▪ Clinician assessment of worsening acne after cycle 13 [all participants]: treatment (154/1561) vs. control (21/522), Peto OR 2.14 [1.49, 3.05], 1 study, n= 2083<br>▪ Discontinuation due to AEs: treatment (290/1591) vs. control (56/535), Peto OR 1.77 [1.36, 2.3], 1 study, n= 2126<br>▪ Discontinuation due to acne: treatment (53/1591) vs. control (1/535), Peto OR 3.56 [1.91, 6.63], 1 study, n= 2126<br>• **DSG/EE vs. CPA/EE or DSG/EE:**<br>o DSG 25-125 µg / EE 40-30 µg versus CPA 2 mg / EE 35 µg:<br>▪ Photographic evaluation of mean change in acne at cycle 4: MD 0.1 [-0.1, 0.3], 1 study, n= 99<br>▪ Women with pustules or nodules at cycle 4: treatment (33/59) vs. control (31/62), Peto OR 1.27 [0.62, 2.58], 1 study, n= 121<br>▪ Mean change in comedone count at cycle 4 MD: 3.7 [-5.39, 12.79], 1 study, n= 121<br>▪ Mean change in papule count at cycle 4: MD -0.6 [-4.16, 2.96], 1 study, n= 121<br>▪ Mean change in pustule count at cycle 4: MD 2.3 [-0.15, 4.75], 1 study, n= 121<br>▪ Women with moderate acne at cycle 6: treatment (32/68) vs. control (29/68), Peto OR 1.19 [0.61, 2.34], 1 study, n= 136<br>▪ Women with severe acne at cycle 6: treatment (4/68) vs. control (2/68), Peto OR 2 [0.39, 10.21], 1 study, n= 136<br>▪ Mean comedone count at cycle 6: MD 2.9 [0.05, 5.75], 1 study, n= 136, favours control<br>▪ Mean papule count at cycle 6: MD 1.8 [-0.4, 4], 1 study, n= 136<br>▪ Mean pustule count at cycle 6: MD 0.8 [-0.35, 1.95], 1 study, n= 136<br>▪ Mean nodule count at cycle 6: MD 0 [-0.18, 0.18], 1 study, n= 136<br>▪ Discontinuation due to non-acne AE: treatment (6/84) vs. control (4/88), Peto OR 1.6 [0.45, 5.73], 1 study, n= 172<br>▪ Discontinuation due to worsening of acne: treatment (1/84) vs. control (1/88), Peto OR 1.05 [0.06, 16.9], 1 study, n= 172<br>o DSG 150 µg / EE 30 µg versus CPA 2 mg / EE 50 µg:<br>▪ Women with moderate or severe acne at cycle 6: treatment (12/26) vs. control (3/31), Peto OR 6.35 [1.96, 20.52], 1 study, n= 57

Extraction at the level of systematic reviews	
	▪ Women with self-assessed acne improvement at cycle 6: treatment (25/26) vs. control (28/31), Peto OR: 2.41 [0.32, 18.18], 1 study, n= 57<br>▪ Discontinuation due to side effects: treatment (2/32) vs. control (1/34), Peto OR 2.12 [0.21, 21.13], 1 study, n= 66<br>o DSG 150 µg / EE 30 µg versus GSD 75 µg / EE 30 µg:<br>▪ Women without acne at cycle 6: treatment (549/619) vs. control (486/561), Peto OR: 1.17 [0.82, 1.66], I^2= 70.68 %, 2 studies, n= 1180<br>▪ Women with mild acne at cycle 6: treatment (57/619) vs. control (68/561), Peto OR: 0.76 [0.52, 1.1], I^2= 67.61%, 2 studies, n= 1180<br>▪ Women with moderate or severe acne at cycle 6: treatment (13/619) vs. control (7/561), Peto OR: 1.78 [0.73, 4.32], I^2= 0%, 2 studies, n= 1180<br>▪ Women with mild or no acne: treatment (10/11) vs. control (5/8), Peto OR 5.05 [0.57, 44.42], 1 study, n= 19<br>▪ Women with improved acne score: treatment (10/11) vs. control (7/8), Peto OR 1.41 [0.08, 25.31], 1 study, n= 19<br>▪ Discontinuation due to side effects: treatment (40/710) vs. control (57/668), Peto OR 0.61 [0.4, 0.93], 1 study, n= 1378<br>o DSG 150 µg / EE 20 µg versus LNG 100 µg / EE 20 µg:<br>▪ Improvement in comedones at week 25: treatment (71/266) vs. control (49/258), Peto OR 1.55 [1.03, 2.32], 1 study, n= 524<br>▪ Worsening in comedones at week 25: treatment (27/266) vs. control (32/258), Peto OR 0.8 [0.46, 1.37], 1 study, n= 524<br>▪ Improvement in papules at week 25: treatment (63/266) vs. control (61/258), Peto OR 1 [0.67, 1.5], 1 study, n= 524<br>▪ Worsening in papules at week 25: treatment (31/266) vs. control (47/258), Peto OR 0.6 [0.37, 0.96], 1 study, n= 524<br>▪ Improvement in pustules at week 25: treatment (46/266) vs. control (32/258), Peto OR 1.47 [0.91, 2.38], 1 study, n= 524<br>▪ Worsening in pustules at week 25: treatment (23/266) vs. control (33/258), Peto OR 0.65 [0.37, 1.13], 1 study, n= 524<br>▪ Improvement in nodules at week 25: treatment (14/266) vs. control (17/258), Peto OR 0.79 [0.38, 1.63], 1 study, n= 524<br>▪ Worsening in nodules at week 25: treatment (8/266) vs. control (7/258), Peto OR 1.11 [0.4, 3.1], 1 study, n= 524<br>▪ Scores for Psychological General Well-Being Index at week 13: MD 1.9 [0.26, 3.54], 1 study, n= 720, favours treatment<br>▪ Scores for Psychological General Well-Being Index at week 25: MD 1.1 [-0.83, 3.03], 1 study, n= 720<br>▪ AEs related to treatment: treatment (31/500) vs. control (32/498), Peto OR 0.96 [0.58, 1.6], 1 study, n= 998<br>▪ AEs not related to treatment: treatment (79/500) vs. control (58/498), Peto OR 1.42 [0.99, 2.04], 1 study, n= 998<br>• **LNG/EE vs. other COCs:**<br>o LNG 150 µg / EE 30 µg versus DSG 150 µg / EE 30 µg:<br>▪ Mean acne severity score at cycle 6:MD 0.5 [0.09, 0.91], 1 study, n= 33, favours control<br>▪ Mean total lesion count at cycle 9: MD 6.3 [-9.93, 22.53], 1 study, n= 16<br>▪ Discontinuation due to side effects: treatment (1/17) vs. control (1/17), Peto OR 1.0 [0.06, 16.69], 1 study, n= 34<br>▪ Discontinuation due to worsening acne: treatment (4/28) vs. control (2/26), Peto OR 1.93 [0.36, 10.36], 1 study, n= 54<br>o LNG 150 µg / EE 30 µg versus CMA 2 mg / EE 30 µg:<br>▪ Women with >= 50% reduction in pustules and papules at cycle 12: treatment (45/98) vs. control (60/101), Peto OR: 0.58 [0.33, 1.02], 1 study, n= 199<br>▪ Women with Plewig score of 0 at cycle 12: treatment (38/70) vs. control (53/79), Peto OR 0.59 [0.30, 1.13], 1 study, n= 149<br>▪ Women with increased pustules or papules lesion count at cycle 12: treatment (8/70) vs. control (0/79), Peto OR 9.34 [2.25, 38.73], 1 study, n= 149<br>▪ Women with comedones improvement at cycle 12: treatment (51/66) vs. control (64/72), Peto OR 0.44 [0.18, 1.06], 1 study, n= 138<br>▪ Women with self-assessed acne improvement at cycle 12: treatment (61/70) vs. control (78/79), Peto OR: 0.16 [0.04, 0.57], 1 study, n= 149<br>o LNG 150 µg / EE 30 µg versus CPA 2 mg / EE 35 µg:<br>▪ Mean change in total acne lesions at cycle 6: MD: 2.5 [-8.81, 13.81], 1 study, n= 80<br>▪ Mean pustule count at cycle 6: MD: 1.8 [0.63, 2.97], 1 study, n= 80, favours control<br>▪ Mean papule count at cycle 6: MD: 2.9 [0.2, 5.6], 1 study, n= 80, favours control<br>▪ Mean cyst and nodule count at cycle 6: MD: 0.4 [-0.13, 0.93], 1 study, n= 80<br>▪ Women with dermatologist global "good" acne assessment at cycle 6: treatment (11/36) vs. control (28/45), Peto OR: 0.29 [0.12, 0.68], 1 study, n= 81<br>▪ Women with "good" acne self-assessment at cycle 6: treatment (11/36) vs. control (30/44), Peto OR: 0.23 [0.09, 0.54], 1 study, n= 80<br>▪ Discontinuation due to side effects: treatment (6/37) vs. control (6/48), Peto OR: 1.35 [0.4, 4.6], 1 study, n= 85<br>o LNG 150 µg / EE 30 µg versus CPA 2 mg / EE 50 µg:<br>▪ Mean change in total acne lesions at cycle 6: MD: 0.1 [-10.79, 10.99], 1 study, n= 81

Extraction at the level of systematic reviews	
	▪ Mean pustule count at cycle 6: MD: 2.1 [0.93, 3.27], 1 study, n= 81, favours control<br>▪ Mean papule count at cycle 6: MD: 3.6 [1.12, 6.08], 1 study, n= 82, favours control<br>▪ Mean cyst and nodule count at cycle 6: MD: 0.4 [-0.11, 0.91], 1 study, n= 81<br>▪ Women with dermatologist global "good" acne assessment at cycle 6: treatment (11/36) vs. control (31/45), Peto OR: 0.22 [0.09, 0.52], 1 study, n= 81<br>▪ Women with "good" acne self-assessment at cycle 6: treatment (11/36) vs. control (33/45), Peto 0.18 [0.08, 0.44], 1 study, n= 81<br>▪ Discontinuation due to side effects: treatment (6/37) vs. control (5/48), Peto OR: 1.66 [0.47, 5.92], 1 study, n= 85<br>○ LNG 250 µg / EE 50 µg versus CPA 2 mg / EE 50 µg:<br>▪ Women with global 'improvement or healing' acne assessment at cycle 6: treatment (31/44) vs. control (29/31), Peto OR: 0.24 [0.08, 0.75], 1 study, n= 75<br>○ LNG 100 µg / EE 20 µg versus NA 1 mg / EE 20 µg:<br>▪ Mean change in total lesion count among subset of women with >= 15 lesions at baseline: MD: 2.5 [-12.26, 17.26], 1 study, n= 19<br>▪ Discontinuation due to side effects: treatment (0/30) vs. control (1/28), Peto OR: 0.13 [0, 6.37], 1 study, n= 58<br>• **Other COCs vs. CPA 2 mg/EE**<br>○ Dienogest 2 mg / EE 30 µg versus CPA 2 mg / EE 35 µg:<br>▪ Mean percentage change in inflammatory lesion count after cycle 6: MD -1 [-4.72, 2.72], 1 study, n= 1037<br>▪ Mean percentage change in total lesion count after cycle 6: MD -1.1 [-4.37, 2.17], 1 study, n= 1043<br>▪ Improvement of facial acne [clinical assessment]: treatment (477/519) vs. control (480/532), Peto OR: 1.23 [0.8, 1.88], 1 study, n= 1051<br>▪ Discontinuation due to reason other than AE: treatment (14/525) vs. control (19/537), Peto OR: 0.75 [0.37, 1.5], 1 study, n= 1062<br>▪ Discontinuation due to AE: treatment (8/525) vs. control (3/537), Peto OR: 2.56 [0.78, 8.4], 1 study, n= 1062<br>○ CPA 2 mg / EE 35 µg versus CPA 2 mg / EE 50 µg:<br>▪ Mean change in total acne lesions at cycle 6: MD -2.4 [-7.15, 2.35], 1 study, n= 89<br>▪ Mean pustule count at cycle 6: MD 0.30 [-0.34, 0.94], 1 study, n= 89<br>▪ Mean papule count at cycle 6: MD 0.70 [-1.47, 2.87], 1 study, n= 90<br>▪ Mean cyst and nodule count at cycle 6: MD 0 [-0.25, 0.25], 1 study, n= 89<br>▪ Women with dermatologist global "good" acne assessment at cycle 6: treatment (28/45) vs. control (31/45), Peto OR 0.75 [0.31, 1.77], 1 study, n= 90<br>▪ Women with "good" acne self-assessment at cycle 6: treatment (30/44) vs. control (33/45), Peto OR 0.78 [0.32, 1.94], 1 study, n= 89<br>▪ Women with healed or improved facial acne lesions at cycle 9: treatment (129/218) vs. control (115/207), Peto OR: 1.16 [0.79, 1.7], 1 study, n= 425<br>▪ Women with severe acne score at cycle 12: treatment (4/40) vs. control (1/33), Peto OR: 2.94 [0.48, 17.99], 1 study, n= 73<br>▪ Discontinuation due to side effects: treatment (6/48) vs. control (5/48), Peto OR 1.23 [0.35, 4.27], 1 study, n= 96<br>○ NGM 180-215-250 µg / EE 35 µg versus CPA 2 mg / EE 35 µg:<br>▪ Mean change in total lesion count at cycle 3: MD -9.16 [-24.98, 6.66], 1 study, n= 45<br>▪ Discontinuation due to adverse event: treatment (4/25) vs. control (1/20), Peto OR: 2.97 [0.47, 18.9], 1 study, n= 45<br>○ CPA 2 mg/EE 50 µg vs. antibiotic:<br>▪ Women with self-assessed acne improvement at cycle 6: treatment (34/39) vs. control (32/39), Peto OR: 1.48 [0.43, 5.01], 1 study, n= 78<br>▪ Women with self-assessed lack of acne at cycle 6: treatment (7/39) vs. control (8/39), Peto OR: 0.85 [0.28, 2.6], 1 study, n= 78<br>▪ Discontinuation due to non-acne adverse event: treatment (4/49) vs. control (3/49), Peto OR: 1.36 [0.29, 6.26], 1 study, n= 98<br>▪ Discontinuation due to lack of acne improvement: treatment (2/49) vs. control (3/49), Peto OR: 0.66 [0.11, 3.95], 1 study, n= 98

AAD guidelines

Extraction at the level of systematic reviews				
**Reference**	**Methodological quality**	**Characteristics I**	**Characteristics II**	**Comments**
Reynolds RV. Guidelines of care for the management of acne vulgaris. J Am Acad Dermatol. 2024 May;90(5):1006.e1-1006.e30. doi: 10.1016/j.jaad.2023.12.017. Epub 2024 Jan 30. PMID: 38300170. https://pubmed.ncbi.nlm.nih.gov/38300170/	AMSTAR-2: critically low	**Objective**<br>• to provide evidence-based recommendations for the management of acne.<br>**Search period**<br>• 2014-05/2021<br>• Updated periodically through May 10, 2022<br>**Databases**<br>• Medline<br>**Population**<br>• Adults, adolescents, and preadolescents [≥9 years] with acne vulgaris<br>**Intervention**<br>• Hormonal agents<br>• Combined oral contraceptives: estrogen and progestins<br>• Aldosterone receptor antagonist: spironolactone<br>• Intralesional steroids: triamcinolone<br>• Oral corticosteroids: prednisolone and prednisone<br>**Control**<br>• Hormonal agents (see above)<br>• Topical agents<br>• Retinoids: adapalene, tazarotene, tretinoin, and trifarotene<br>• Benzoyl peroxide<br>• Topical antibiotics: clindamycin, dapsone, erythromycin, and minocycline<br>• Alpha hydroxy acid: glycolic acid<br>• Beta hydroxy acid: salicylic acid<br>• Azelaic acid<br>• Topical antiandrogen: clascoterone<br>• Others: sulfur/Sulfacetamide sodium, resorcinol<br>• Combinations of topical agents<br>• Systemic Antibiotics<br>• Tetracyclines: doxycycline, minocycline, and sarecycline<br>• Macrolides: azithromycin, clarithromycin, and erythromycin<br>• Penicillins: amoxicillin and ampicillin<br>• Cephalosporin: cephalexin<br>• Trimethoprim/sulfamethoxazole<br>• Others: dapsone<br>• Retinoid: isotretinoin<br>• Physical modalities	**Relevant inclusion criteria**<br>≥ 4 weeks follow up<br>**Relevant exclusion criteria**<br>• Acneiform skin eruptions<br>• Children aged younger than 9 years<br>**Types of studies**<br>Randomized controlled trials, pooled analyses, systematic reviews of RCTs<br>**Outcomes**<br>Outcomes rated as critical or important:<br>1. Physicians' global/local evaluation (e.g., Investigator global assessment scale/score, Global acne grading system, Global Acne Severity Score, etc.)<br>2. Change in lesion counts (e.g., total Inflammatory and non-inflammatory lesions, inflammatory lesions, non-inflammatory lesions, mean % change or mean absolute change in total/inflammatory lesion count, etc.)<br>3. Serious adverse events (e.g., life threatening event, death, etc.)<br>4. For COC: thromboembolic events<br>5. Participants' global self-assessment of acne improvement (e.g., patient's improvement assessment in treatment area appearance, patient's improvement assessment measured by a 4-point scale, etc.)<br>6. Withdrawal due to adverse events<br>7. Quality of Life (e.g., Dermatology Life Quality Index, Dermatology-Specific Quality of Life, Skindex, Assessment of Quality of Life, Acne Disability Index, The Cardiff Acne Disability Index, etc.)<br>8. For spironolactone: hyperkalemia<br>9. For spironolactone: cancer (e.g. breast cancer)<br>10. For spironolactone teratogenicity<br>11. Acne reoccurrence<br>12. Any adverse events (assessed as the total number of participants who experienced at least adverse event)<br>13. For COC: mood disturbance<br>14. For spironolactone: menstrual irregularities<br>**Follow up:** ≥ 4 weeks<br>**Study size:** n ≥ 50 per group	

<table>
<tr><th colspan="5">Extraction at the level of systematic reviews</th></tr>
<tr><td rowspan="3"></td><td></td><td>• Chemical peels: alpha hydroxy acid [glycolic acid, lactic acid, mandelic acid] and beta hydroxy acid [salicylic acid]
• Comedo extraction
• Lasers
• Photodynamic/Light therapies: blue light therapy, red light therapy,
• ALA, and IPL
• Any combination therapy of the above
Control / Placebo / No treatment</td><td></td><td></td></tr>
<tr><td colspan="4">Results</td></tr>
<tr><td colspan="4">Baseline characteristics
• 27 RCTs included
• 8 comparisons:
o COC to placebo (data for all COCs were pooled): n= 1
o different COC groups: n= 6
o spironolactone to placebo: n= 1
Comparisons
• Any COC versus placebo:
o Physician global assessment, clear or almost clear after cycle 6: 191/499 (38.3%) vs. 138/504 (27.4%); RR 1.45 [1.06, 1.97], 3 RCTs, I^2= 65%
o Physician global assessment, no, minimal or mild facial acne after cycle 6: 124/280 (44.3%) vs. 82/275 (29.8%) RR 1.49 [1.19, 1.86], 1 RCT
o Physician global assessment, improvement after cycle 6: 621/682 (91.1%) vs. 302/422 (71.6%) RR 1.29 [1.15, 1.44], 3 RCTs, I^2= 49%
o Physician global assessment, VAS score, after cycle 6: Changes from baseline for both treatment groups revealed significant increases in investigator VAS scores, which were increasingly larger in the DSGOC group over time. Significant differences at endpoint of about 11 mm [p = 0.04] showed a beneficial treatment effect for DSG-OC.*Used a 100-mm visual analog scale [VAS], with higher scores reflecting a better skin appearance*, 1 RCT
o Total lesion, change from baseline after cycle 6: MD -9.69 [-14.58, -4.8], I^2= 34%, 4 RCTs, n= 959, favours COC
o Total lesion, percentage change from baseline after cycle 6: MD -16.6 [-20.8, -12.4], I^2= 8%, 8 RCTs, n= 1506, favours COC
o Inflammatory lesion, change from baseline after cycle 6: MD -3.23 [-4.64, -1.81], 4 RCTs, I^2= 0%, n= 959, favours COC
o Inflammatory lesion, percentage change from baseline after cycle 6: MD -15.81 [-20.44, -11.7], I^2= 9%, 9 RCTs, n= 3220, favours COC
o Non-inflammatory lesion, change from baseline after cycle 6: MD -6.37 [-10.87, -1.88], I^2= 44%, 4 RCTs, n= 959, favours COC
o Non-inflammatory lesion, percentage change from baseline after cycle 6: MD -19.45 [-29.9, -9], I^2= 0%, 6 RCTs, favours COC
o Serious AE: 2/471 (0.4%) vs. 2/472 (0.4%) RR 0.96 [0.14, 6.49], I^2= 0%, 2 RCTs
o Patient global assessment, improvement after cycle 6: 487/591 (82.4%) vs. 371/587 (63.2%) RR 1.31 [1.17, 1.48], I^2= 56%, 4 RCTs
o Patient global assessment after cycle 6: no effect estimate calculated, "Changes from baseline for both treatment groups in Katz 2000 revealed significant increases in patient VAS scores [a 100-mm visual analog scale with high scores reflecting a better skin appearance), which were increasingly larger in the DSG-OC group over time. Significant differences at the endpoint of about 14 mm (p = 0.03) showed a beneficial treatment effect for DSG-OC. At the end of cycle 6 in Plewig 2009 study, at least 70.5% of women taking EE/CMA rated the improvement of their moderate acne lesions as completely resolved (2.8%), excellent improved (37.1%) or satisfactory improved (30.7%), whereas 41.3% of women of the placebo group stated the improvement of their moderate acne lesions as at least satisfactory. “No moderate acne improvement” was reported by 11.6% of subjects taking EE/CMA compared with 19.8%of subjects taking placebo, and a “deterioration” was reported by 6% vs. 13.5% of subjects, respectively", 2 RCTs.
o Withdrawal due to AE: 104/2034 (5.1%) vs. 48/1643 (2.9%) RR 1.86 [1.32, 2.62], I^2= 0%, 8 RCTs
o Acne QoL, self-perception after cycle 6: MD 3.45 [1.87, 5.03], 1 RCT, n= 450, favours COC
o Acne QoL, role-emotional, after cycle 6: MD 4.03 [2.41, 5.65], 1 RCT, n= 450, favours COC
o Acne QoL, role-social, after cycle 6: MD 2.58 [1.42, 3.74], 1 RCT, n= 448, favours COC
o Acne QoL, acne symptoms, after cycle 6: MD 2.96 [1.86, 4.06], 1 RCT, n= 446, favours COC
o Any AE: 668/1236 (54.0%) vs. 424/847 (50.1%) RR 1.15 [1.07, 1.24], I^2= 0%, 4 RCTs
• DRSP 3 mg/EE 30 µg vs. LNG 150 µg/ EE 30 µg: 1 RCT</td></tr>
</table>

**Extraction at the level of systematic reviews**	
	○ Total lesion after cycle 7: no effect estimate calculated, "Mean total lesion count decreased in the DRSP 3mg/EE 30 mg group with ongoing treatment, but remained relatively static in the LNG 150 mg/EE 30 mg group. Consistent with the changes in mean total lesion counts, there was a decrease in the proportion of subjects with acne in the DRSP 3mg/EE 30 mg group, but not in the LNG 150 mg/EE 30 mg group.", 1 RCT<br>○ Deep vein thrombosis: 1/282 (0.4%) vs. 0/142 (0.0%) RR 1.52 [0.06, 36.98], 1 RCT<br>○ Serious AE: 2/282 (0.7%) vs. 0/142 (0.0%) RR 2.53 [0.12, 52.28], 1 RCT<br>○ Any AE: 109/282 (38.7%) vs. 62/142 (43.7%) RR 0.89 [0.70, 1.12], 1 RCT<br>• LNG 150 µg / EE 30 µg vs. DSG 150 µg / EE 30 µg: 2 RCTs<br>○ Mean acne severity score after cycle 6: 1.26 ± 0.56 vs. 0.78 ± 0.66; MD 0.48 [0.06, 0.9], 1 RCT, n= 33<br>○ Mean total lesion count after cycle 9: 17.6 ± 7.6 vs. 11.3 ± 3.3; MD 6.3 [0.77, 11.83], 1 RCT, n= 16<br>○ Total lesion, percentage change from baseline, after cycle 9: no effect estimate calculated, "In subjects completing 9 months of therapy, acne decreased by 52.8% in the LNG/EE group (n = 9) and by 58.5% in the DSG/EE (n = 7) (between groups: P not significant).", 1 RCT<br>○ Withdrawal due to AE: 4/45 (8.9 %) vs. 3/43 (7.0%); RR 1.27 [0.30, 5.35], 2 RCTs<br>• DSG 150 µg / EE 20 µg vs. LNG 100 µg / EE 20 µg: 1 RCT<br>○ Improvement in comedones after cycle 6: 71/266 (26.7%) vs. 49/258 (19.0%) RR 1.41 [1.02, 1.94], 1 RCT<br>○ Improvement in papules after cycle 6: 63/266 (23.7%) vs. 61/258 (23.6%) RR 1.00 [0.74, 1.36], 1 RCT<br>○ Improvement in pustules after cycle 6: 46/266 (17.3%) vs. 32/258 (12.4%) RR 1.39 [0.92, 2.12], 1 RCT<br>○ Improvement in nodules after cycle 6: 14/266 (5.3%) vs. 17/258 (6.6%) RR 0.80 [0.40, 1.59], 1 RCT<br>○ Withdrawal due to AE: 10/500 (2.0%) vs. 26/498 (5.2%) RR 0.38 [0.19, 0.79], 1 RCT<br>○ Scores for Psychological General Well-Being Index after cycle 6: MD 1.1 [-0.83, 3.03], 1 RCT, n= 516<br>• NGM/EE (triphasic) vs. DSG/EE (biphasic): 1 RCT<br>○ Physician global assessment, excellent or good, after cycle 6: 81/93 (87.1%) vs. 71/95 (74.7%); RR 1.17 [1.01, 1.34], 1 RCT<br>○ Total lesion, change from baseline after cycle 6: -13.4 ± 9.7 vs. -11.9 ± 10.1, MD -1.5 [-4.33, 1.33], 1 RCT, n= 188<br>○ Total lesion, percentage change from baseline, after cycle 6: -74.4 ± 35.2 vs. -65.1 ± 35.2, MD -9.3 [-19.36, 0.76], 1 RCT, n= 188<br>○ Papules, change from baseline after cycle 6: -3.5 ± 5.1 vs. -2.8 ± 4.6, MD -0.7 [-2.09, 0.69], 1 RCT, n= 188<br>○ Pustules and Nodules, change from baseline after cycle 6: -0.9 ± 2.3 vs. -1.0 ± 2.1 MD 0.1 [-0.53, 0.73], 1 RCT, n= 188<br>○ Comedones, change from baseline after cycle 6: -9.0 ± 6.3 vs. -8.2 ± 7.4 MD -0.8 [-2.76, 1.16], 1 RCT, n= 188<br>○ Serious AE: 2/100 (2.0%) vs. 0/101 (0.0%), RR 5.05 [0.25, 103.87], 1 RCT<br>○ Patient global assessment, excellent or good after cycle 6: 86/93 (92.5%) vs. 91/95 (95.8%) RR 0.97 [0.90, 1.04], 1 RCT<br>• DRSP 3 mg / EE 30 µg vs. NGM 180-215-250 µg / EE 35 µg: 1 RCT<br>○ Physician global assessment, Improvement of facial acne after cycle 6: 527/551 (95.6%) vs. 524/569 (92.1%) RR 1.04 [1.01, 1.07], 1 RCT<br>○ Total lesion, percentage change from baseline after cycle 6: no information reported, MD -3.3 [-6.45, -0.15], 1 RCT, n= 1108<br>○ Total lesion, change from baseline after cycle 6: no information reported, MD -2.6 [-5.72, 0.52], 1 RCT, n= 1108<br>○ Inflammatory lesion, percentage change from baseline after cycle 6: no information reported, MD -2.4 [-5.97, 1.17], 1 RCT, n= 1108<br>○ Inflammatory lesion, change from baseline after cycle 6: no information reported, MD -0.5 [-1.86, 0.86], 1 RCT, n= 1108<br>○ Open comedone, percentage change from baseline after cycle 6: no information reported, MD -9.3 [-17.59, -1.01], 1 RCT, n= 1108<br>○ Open comedone, change from baseline after cycle 6: no information reported, MD -1.5 [-3.17, 0.17], 1 RCT, n= 1108<br>○ Closed comedone, percentage change from baseline after cycle 6: no information reported, MD -5.2 [-13.45, 3.05], 1 RCT<br>○ Closed comedone, change from baseline after cycle 6: no information reported, MD -0.6 [-2.1, 0.9], 1 RCT<br>○ Serious AE: 18/566 (3.2%) vs. 2/582 (0.3%) RR 9.25 [2.16, 39.70], 1 RCT<br>○ Patient global assessment, improvement of facial acne after cycle 6: 512/550 (93.1%) vs. 505/567 (89.1%) RR 1.05 [1.01, 1.08], 1 RCT<br>○ Discontinuation due to adverse event: 18/566 (3.2%) vs. 23/582 (4.0%) RR 0.80 [0.44, 1.47], 1 RCT<br>○ Any AE: 92/566 (16.3%) vs. 106/582 (18.2%) RR 0.89 [0.69, 1.15], 1 RCT<br>• LNG 100 µg / EE 20 µg vs. NA 1 mg / EE 20 µg: 1 RCT<br>○ Total lesion, change from baseline after cycle 3 [subgroup of women with ≥ 15 lesions at baseline]: -18.4±15.8 vs. -20.9±16.5, MD 2.5 [-12.26, 17.26], 1 RCT, n= 19

**Extraction at the level of systematic reviews**	
	○ Discontinuation due to side effects: 0/30 (0.0%) vs. 1/28 (3.6%) RR 0.31 [0.01, 7.35], 1 RCT<br>• Spironolactone vs. placebo: 3 RCTs<br>○ Physician global assessment, clear or almost clear, at 12 weeks: 15/20 (75.0%) vs. 6/20 (30.0%) RR 2.50 [1.22, 5.11], 1 RCT<br>○ Physician global assessment, improved, at 12 weeks: 3/10 (30.0%) vs. 1/6 (16.7%) RR 1.80 [0.24, 13.63], 1 RCT<br>○ Total lesion, change from baseline, at 12 weeks: -18.2 ± 17.6 vs. -38 ± 36.6 MD 19.75 [2.39, 37.11], 1 RCT, n= 42<br>○ Total lesion, ≥ 50% reduction, at 12 weeks: 15/20 (75.0%) vs. 4/20 (20.0%) RR 3.75 [1.51, 9.34], 1 RCT<br>○ Inflammatory lesion, change from baseline, at 12 weeks: -4.0 ± 7.1 vs. -6.5 ± 5.9 MD 2.45 [-1.52, 6.42], 1 RCT, n= 42<br>○ Noninflammatory lesion, change from baseline, at 12 weeks: -25.0 ± 15.4 vs. -35.1 ± 35.8 MD 10.1 [-6.57, 26.77], 1 RCT, n= 42<br>○ Patient global assessment, improved, at 12 weeks: 24/31 (77.4%) vs. 6/27 (22.2%) RR 3.60 [1.75, 7.42], 2 RCTs<br>○ Withdrawal due to AE: effect not estimable, 0 pts. in both groups, 1 RCT, n= 40<br>○ Menstrual irregularities: 13/32 (40.6%) vs. 0/27 (0.0%) RR 10.89 [1.54, 77.08], 2 RCTs

Extraction at the level of individual studies following the search period of the AAD guidelines

Extraction at the level of individual studies following the search period of the AAD guidelines

Reference	Study type	Characteristics	Intervention / Comparison	N	Results
**Spironolactone vs. placebo**					
Santer M, et al.: Effectiveness of spironolactone for women with acne vulgaris (SAFA) in England and Wales: pragmatic, multicentre, phase 3, double blind, randomised controlled trial. BMJ. 2023 May 16;381:e074349. PMID: 37192767 https://pubmed.ncbi.nlm.nih.gov/37192767/	RCT, double-blind<br><br>RoB 2.0 (overall): some concerns for efficacy, QoL and safety at 12 weeks and high risk of bias for efficacy, QoL and safety at 24 weeks	**Population:** Women (≥18 years) with facial acne for at least six months<br>**Study duration:** 24 weeks, followed by an unblinded follow-up period for up to 52 weeks<br>**Outcomes:**<br>Primary: mean Acne-QoL symptom subscale score at 12 weeks<br>Secondary: (see column results)<br>**Funding:** funded by the National Institute for Health and Care Research under its Health Technology Assessment programme	50 mg/day spironolactone, increasing to 100 mg/day spironolactone until week 24 + individual topical standard treatment	n = 201 received intervention, n = 176 included in primary outcome complete cases analysis	
			Placebo + individual topical standard treatment	n = 209 received placebo, n = 166 included in primary outcome complete cases analysis	

Baseline	Spironolactone (n = 201)	Placebo (n = 209)
Mean age (years)	29.6 ± 7.4 (n = 201)	28.7 ± 7.0 (n = 209)
Polycystic ovary syndrome diagnosis or suspected	30 (15%) (n = 195)	47 (23%) (n = 202)
Acne-QoL symptom subscale score Mean (SD)	13.2 ± 4.9 (n = 201)	12.9 ± 4.5 (n = 209)
IGA 3 or more	109 (54%) (n = 201)	111 (53%) (n = 209)

Post treatment	wk	Spironolactone	Placebo	Effect estimate (adjusted*)
Acne-QoL symptom subscale Mean (SD)	12<br>24	19.2 ± 6 (n= 176)<br>21.2 ± 6 (n= 163)	17.8 ± 6 (n= 166)<br>17.4 ± 6 (n= 136)	MD: 1.27 (0.07; 2.46)<br>MD: 3.45 (2.16; 4.75)
Self-assessed overall improvement score of 3-6	12<br>24	122/169 (72%)<br>131/160 (82%)	108/159 (68%)<br>81/128 (63%)	OR: 1.16 (0.70; 1.91)<br>OR: 2.72 (1.50; 4.93)
Satisfaction with trial treatment, score 3-5	24	101/143 (71%)	53/123 (43%)	OR: 3.12 (1.80; 5.41)
PGA success score	12<br>24	36/176 (21%)<br>53/164 (32%)	20/166 (12%)<br>15/136 (11%)	OR: 1.69 (0.89; 3.19)<br>OR: 3.76 (1.95; 7.28)
IGA success score	12	31/168 (19%)	9/160 (6%)	OR: 5.18 (2.18; 12.28)
Irregular menstrual bleeding	12 - 24	57 (32%)	61 (35%)	n.r.
At least one adverse reaction	n.i.	128/201 (64%)	107/209 (51%)	n.r.
Serious adverse reactions	n.i.	0/201 (0%)	0/209 (0%)	n.r.
Dropout due to unacceptable side effects	n.i.	2/201 (1%)	2/209 (1%)	n.r.

Extraction at the level of individual studies following the search period of the AAD guidelines

Reference	Study type	Characteristics	Intervention / Comparison	N	Results
					* Adjustment: stratification factors, baseline Acne-QoL symptom subscale score, topical treatment use, hormonal treatment use, age, and polycystic ovary syndrome status
**Spironolactone vs. doxycycline + placebo**					
Dréno B, et al.: Efficacy of Spironolactone Compared with Doxycycline in Moderate Acne in Adult Females: Results of the Multicentre, Controlled, Randomized, Double-blind Prospective and Parallel Female Acne Spironolactone vs doxyCycline Efficacy (FASCE) Study. Acta Derm Venereol. 2024 Feb 21;104:adv26002. PMID: 38380975; https://pubmed.ncbi.nlm.nih.gov/38380975/	RCT, double-blind<br><br>RoB 2.0 (overall): high risk of bias for efficacy, QoL and safety	**Population:**<br>female patients (≥20 years) with moderate acne (≥10 inflammatory lesions and ≤3 nodules according to the AFAST scoring tool)<br>**Study duration:** 6 months double-blind treatment followed by a 6-month open-labelled maintenance period<br>**Outcomes:**<br>Primary: best rate of success in each arm between Month 4 and Month, defined by a decrease of both Adult Female Acne Scoring Tool (AFAST) scores 1 and 2<br>Sencondary: see results<br>**Funding:** This study was supported by a grant from the French Ministry of Health (PHRC-16-0290).	Spironolactone 150mg/day+ benzoyl peroxide 5% **for 6 months**	n = 71 randomized,<br>n = 65 analyzed,<br>n = 63 analyzed (PP)	(see results tables below)
			Doxycycline 100mg/day + benzoyl peroxide 5% **for 3 months**, followed by placebo for 3 months + benzoyl peroxide 5% for 6 months	n = 76 randomized,<br>n = 68 analyzed,<br>n = 66 analyzed (PP)	

	**Spironolactone (n = 65);** Mean (SD)	**Doxycycline + placebo (n = 68);** Mean (SD)
**Baseline**		
Mean age (years)	27.52 ± 6.6	29.75 ± 7.90
AFAST Score Global	2.71 ± 0.50	2.70 ± 0.50
ECLA score	3.2 ± 2.3	2.9 ± 2.4
Inflammatory lesion count (*n*)	17.6 ± 5.6	20.3 ± 15.3
Non-inflammatory lesion count (*n*)	19.8 ± 14.5	17.4 ± 9.3
CADI score (0–15)	6.24 ± 2.64	6.32 ± 2.65
EQ-5D score (1–5)	5.7 ± 0.8	5.7 ± 0.9

**Post treatment**	**Follow up**	**Spiro**	**Doxy**	**Effect estimate**
AFAST Score Global	4 mo	Data only available graphically, see Fig. 4a in publication.		OR 1.37 (95% CI 0.60; 3.12)
	6 mo			OR 2.87 (95% CI 1.38; 5.99), in favour of Spironolactone
ECLA score	4 and 6 mo	Data only available graphically, see Fig. 5 in publication.		n.r.
Inflammatory lesion count	4 mo	12.7	13.0	n.r.

Extraction at the level of individual studies following the search period of the AAD guidelines

Reference	Study type	Characteristics	Intervention / Comparison	N	Results				
					(mean delta)	6 mo	14.4	12.9	n.r.
					Non-inflammatory lesion count (mean delta)	4 mo	9.2	5.6	n.r.
						6 mo	12.2	6.2	n.r.
					CADI score (0–15) mean (SD)	4 mo	Data only available graphically, see Fig. 7.		n.r.
						6 mo	2.56 ± 2.38	3.44 ± 2.44	n.r.
					EQ-5D score (1–5)	4 mo	n.r.	n.r.	n.r.
						6 mo	5.5 ± 0.8	5.8 ± 0.7	n.r.
					Number of patients with ≥1 adverse event (AE)		68/n.r.	50/n.r.	n.r.
					Number of serious adverse events		4	2	n.r.
					Dropout due to AE		0/n.r.	0/n.r.	n.r.
					non-serious AEs, related to treatment		n= 23	n.r.	n.r.

Risk of bias appraisal

Reference	Outcome	Judgement for "randomization process"	Judgement for "deviations from the intended interventions (assignment)"	Judgement for "missing outcome data"	Judgement for "measurement of the outcome"	Judgement for "selection of the reported result"	Overall risk of bias judgement
Dréno B, et al.: Efficacy of Spironolactone Compared with Doxycycline in Moderate Acne in Adult Females: Results of the Multicentre, Controlled, Randomized, Double-blind Prospective and Parallel Female Acne Spironolactone vs doxyCycline Efficacy (FASCE) Study. Acta Derm Venereol. 2024 Feb 21;104:adv26002. PMID: 38380975	1. AFAST Score Global at m6<br>2. ECLA score at m6<br>3. Inflammatory lesion count (mean delta)´at m6<br>4. Non-inflammatory lesion count (mean delta) at m6<br>5. CADI score at m6<br>6. EQ-5D score at m6	low risk of bias	high risk of bias	high risk of bias	low risk of bias	low risk of bias	**high risk of bias**
	Adverse events throughout the entire course of the study			low risk of bias	low risk of bias		**high risk of bias**
Santer M, et al.: Effectiveness of spironolactone for women with acne vulgaris (SAFA) in England and Wales: pragmatic, multicentre, phase 3, double blind, randomised controlled trial. BMJ. 2023 May 16;381:e074349. PMID: 37192767	1. Acne QoL symptom subscale score at w12 and w24<br>2. Self-assessed overall improvement score at w12 and w24<br>3. Participant's global assessment (PGA) success score at w12 and w24<br>4. IGA success score at w12	some concerns	low risk of bias	QOL 12 wks: low risk of bias<br><br>O 2-4: 12 wks: some concerns<br><br>O 1-4 24 wks: high risk of bias	low risk of bias	low risk of bias	**O 1-4 12 wks: some concerns**<br><br>**O 1-4 24 wks: high risk of bias**
	Side effects at w6, w12, w24 and up to w52			12 wks: some concerns<br><br>24 wks: high risk of bias	low risk of bias		**12 wks: some concerns**<br><br>**24 wks: high risk of bias**

List of abbreviations

COC	Combined oral contraceptive
EE	Ethinyl estradiol
LNG	Levonorgestrel
CPA	Cyproterone acetate
CMA	Chlormadinone acetate
DSG	Desogestrel
NA	Norethindrone acetate
NGM	Norgestimate
DRSP	Drospirenone
NOMAC	Nomegestrol acetate
GSD	Gestodene

Key question 3: New topical treatments

Comedonal acne

New Topicals													
**Author(s)**	**Intervention**	**N**	**S**	**D**	**B**	**%↓NIL**	**other Outcomes/ statistics**	**Comments**	**Summary of efficacy**	**Safety**	**Drop outs**	**Summary of safety**	
**Trifarotene vs. Placebo**													
Tan 2019[1] PERFECT 1 (face)	once-daily trifarotene 50 µg/g	612	IGA 3 (moderate)	12	PI	49.7	P< 0.001		once-daily trifarotene 50 µg/g > vehicle	pts. with severe drug related AEs: n= 6 pts.	72	Placebo better safety than trifarotene	
	vehicle cream	596				35.7				pts. with severe drug related AEs: n= 0 pts.	61		
Tan 2019[1] PERFECT 2 (face)	once-daily trifarotene 50 µg/g	602	IGA 3 (moderate)	12	PI	57.7	P< 0.001		once-daily trifarotene 50 µg/g > vehicle	pts. with severe drug related AEs: n= 3 pts.	44	Placebo better safety than trifarotene	
	vehicle cream	610				43.9				pts. with severe drug related AEs: n= 0 pts.	37		
Tan 2019[1] PERFECT 1 (trunk)	once-daily trifarotene 50 µg/g	612	IGA 3 (moderate)	12	PI	49.1	P< 0.001		once-daily trifarotene 50 µg/g = vehicle	see above			
	vehicle cream	596				40.3				see above			
Tan 2019[1] PERFECT 2 (trunk)	once-daily trifarotene 50 µg/g	602	IGA 3 (moderate)	12	PI	55.2	P< 0.001		once-daily trifarotene 50 µg/g > vehicle	see above			

New Topicals													
**Author(s)**	**Intervention**	**N**	**S**	**D**	**B**	**%↓NIL**	**other Outcomes/ statistics**	**Comments**	**Summary of efficacy**	**Safety**	**Drop outs**	**Summary of safety**	
	vehicle cream	610				45.1				see above			
Schleicher 2023[2] (face)	once-daily trifarotene 50 µg/g	121 (split-face)	IGA 3 or 4 (moderate or severe)	24	PI	61.4	P< 0.05	-	once-daily trifarotene 50 µg/g > vehicle	pts. with treatment related AE: 5.0 %	1 discontinuation due to AE	Placebo better safety than trifarotene	
	vehicle cream					32.1				pts. With treatment related AE: 1.7 %			
Alexis 2024[3] (face)	once-daily trifarotene 50 µg/g	60	IGA 3 (moderate)	24	PI	n.r.	total lesion score: -72.0%	P < 0.05		pts. with AE: 10/60 (16.7%)*	1 discontinued due to AE not related to study treatment.	Trifarotene better saftey than placebo	
	vehicle cream	63				n.r.	total lesion score: -62.8%			pts. with AE: 19/63 (30.2%)* *not drug related			
**Summary efficacy (face):**<br>superior efficacy once-daily trifarotene 50 µg/g > vehicle (range reduction NIL: Trifarotene: 49.7 - 61.4%, vehicle: 32.1-43.9%), 3 RCTs<br>**Summary efficacy (trunk):**<br>superior efficacy once-daily trifarotene 50 µg/g > vehicle, 1 RCTs<br>comparable efficacy once-daily trifarotene 50 µg/g = vehicle, 1 RCTs													
**Summary safety:** placebo showed better safety than trifarotene, but conflicting evidence for non drug-related AE													
**Trifaroten + systemic Doxycycline vs. Vehicle + Placebo**													
Del Rosso 2022[4] (face)	once-daily trifarotene 50 µg/g (T) + enteric-coated doxycycline (D) 120mg	133	IGA 4	12	PI	n.r.	mean absolute change in NIL: -39.5	P< 0.0001	T + D > vehicle + placebo	treatment related AE: 13.5 % (18/133)	10	Trifarotene + doxycycline = vehicle + placebo	

New Topicals													
**Author(s)**	**Intervention**	**N**	**S**	**D**	**B**	**%↓NIL**	**other Outcomes/ statistics**	**Comments**	**Summary of efficacy**	**Safety**	**Drop outs**	**Summary of safety**	
	vehicle cream + doxycycline placebo	69				n.r.	mean absolute change in NIL: -28.2			treatment related AE: 15.9 % (11/69)	4		
**Summary efficacy:** superior efficacy once-daily trifarotene 50 µg/g + Doxycycline 120 mg > vehicle + Placebo, 1 RCT													
**Summary safety**: comparable safety once-daily trifarotene 50 µg/g + Doxycycline 120 mg = vehicle + Placebo, 1 RCT													
**Clascoterone vs. Placebo**													
Hebert 2020[5] CB-03-01/25 (face)	1% clascoterone cream; BID	353	IGA 3 or 4	12	PI	30.6	P=0.009	-	1% clascoterone cream = vehicle cream	Patients with ≥1 TEAE: 40/353 (11.3%)	66	Clascoterone = Vehicle	
	vehicle cream	355				21.6				Patients with ≥1 TEAE: 41/355 (11.5%)	65		
Hebert 2020[5] CB-03-01/26 (face)	1% clascoterone cream; BID	369	IGA 3 or 4	12	PI	29.3	P<0.001	-	1% clascoterone cream > vehicle cream	Patients with ≥1 TEAE: 42/369 (11.4%)	67	Clascoterone = Vehicle	
	vehicle cream	363				15.6				Patients with ≥1 TEAE: 50/363 (13.8%)	81		
Mazzetti 2019[6] (face)	clascoterone 0.1% cream; BID	72	IGA 2 - 4	12	PI	n.r.	median [range]: -10.0 [-50 to +69]	only absolute change, non-inflammatory lesions at week 12 vs. baseline reported<br><br>clascoterone 1% BID group had significantly greater decrease (P<0.05) than the clascoterone 0.5% BID, clascoterone 1% QD, and vehicle groups at week 12/EOS from baseline		Number of Subjects with AE: 18 (25.0%)	59	Clascoterone = Vehicle	
	clascoterone 0.5% cream; BID	76				n.r.	median [range]: -10.0 [-56 to +171]			Number of Subjects with AE: 29 (38.2%)			
	clascoterone 1% cream; QD	70				n.r.	median [range]: -6.0 [-48 to +85]			Number of Subjects with AE: 16 (22.9%)			

New Topicals													
**Author(s)**	**Intervention**	**N**	**S**	**D**	**B**	**%↓NIL**	**other Outcomes/ statistics**	**Comments**	**Summary of efficacy**	**Safety**	**Drop outs**	**Summary of safety**	
	clascoterone 1% cream; BID	70				n.r.	median [range]: -17.5 [-63 to +34]			Number of Subjects with AE: 13 (18.6%)			
	vehicle cream BID or QD	75				n.r.	median [range]: -9.0 [-45 to +64]			Number of Subjects with AE: 17 (22.7%)			
**Summary efficacy:**<br>superior efficacy 1% clascoterone BID > vehicle, 1 RCT<br>comparable efficacy 1% clascoterone BID = vehicle, 1 RCT													
Summary safety/ tolerability: clascoterone 1% showed comparable safety to vehicle cream													

Abbreviations: N= number, S = severity of acne, D= duration, B= Blinding, NIL= non-inflammatory lesion

Papulopustular acne

New Topicals													
**Author(s)**	**Intervention**	**N**	**S**	**D**	**B**	**%↓IL**	**other Outcomes/ statistics**	**Comments**	**Summary of efficacy**	**Safety/tolerabilty**	**Drop outs**	**Summary of safety**	
**Trifarotene vs. Placebo**													
Tan 2019[1] PERFECT 1 (face)	once-daily trifarotene 50 µg/g	612	IGA 3 (moderate)	12	PI	54.4	P< 0.001		once-daily trifarotene 50 µg/g = vehicle	pts. with severe drug related AEs: n= 6 pts.	72	Placebo better safety than trifarotene	
	vehicle cream	596				44.8				pts. with severe drug related AEs: n= 0 pts.	61		
Tan 2019[1] PerfECT 2 (face)	once-daily trifarotene 50 µg/g	602	IGA 3 (moderate)	12	PI	66.2	P< 0.001		once-daily trifarotene 50 µg/g > vehicle	pts. with severe drug related AEs: n= 3 pts.	44	Placebo better safety than trifarotene	
	vehicle cream	610				51.2				pts. with severe drug related AEs: n= 0 pts.	37		

New Topicals													
**Author(s)**	**Intervention**	**N**	**S**	**D**	**B**	**%↓IL**	**other Outcomes/ statistics**	**Comments**	**Summary of efficacy**	**Safety/tolerabilty**	**Drop outs**	**Summary of safety**	
Tan 2019[1] PERFECT 1 (trunk)	once-daily trifarotene 50 µg/g	612	IGA 3 (moderate)	12	PI	57.4	P< 0.001		once-daily trifarotene 50 µg/g = vehicle	see above			
	vehicle cream	596				50.0				see above			
Tan 2019[1] PerfECT 2 (trunk)	once-daily trifarotene 50 µg/g	602	IGA 3 (moderate)	12	PI	65.4	P< 0.001		once-daily trifarotene 50 µg/g > vehicle	see above			
	vehicle cream	610				51.1				see above			
Schleicher 2023[2] (face)	once-daily trifarotene 50 µg/g	121 (split-face design)	IGA 3 or 4 (moderate or severe)	24	PI	76.3	P< 0.05		once-daily trifarotene 50 µg/g > vehicle	pts. with treatment related AE: 5.0 %	1 discontinuation due to AE	Placebo better safety than trifarotene	
	vehicle cream					48.3				pts. With treatment related AE: 1.7 %			
Alexis 2024[3] (face)	once-daily trifarotene 50 µg/g	60	IGA 3 (moderate)	24	PI	n.r.	total lesion score: -72.0%	P < 0.05		pts. with AE: 10/60 (16.7%)*	1 discontinued due to AE not related to study treatment.	Trifarotene better saftey than placebo	
	vehicle cream	63				n.r.	total lesion score: -62.8%			pts. with AE: 19/63 (30.2%)* *not drug related			

Author(s)	Intervention	N	S	D	B	%↓IL	other Outcomes/ statistics	Comments	Summary of efficacy	Safety/tolerabilty	Drop outs	Summary of safety
**New Topicals**												
**Summary efficacy (face):**<br>superior efficacy once-daily trifarotene 50 µg/g > vehicle (range reduction IL: Trifarotene: 66.2 - 76.3%, vehicle: 48.3-51.2%), 2 RCTs<br>comparable efficacy once-daily trifarotene 50 µg/g = vehicle, 1 RCTs<br>Summary efficacy (trunk):<br>superior efficacy once-daily trifarotene 50 µg/g > vehicle, 1 RCTs<br>comparable efficacy once-daily trifarotene 50 µg/g = vehicle, 1 RCTs												
**Summary safety:** Placebo showed better safety than trifarotene, but conflicting evidence for non drug-related AE												
**Trifaroten + Doxycycline vs. Placebo**												
Del Rosso 2022[4] (face)	once-daily trifarotene 50 µg/g (T) + enteric-coated doxycycline (D) 120mg	133	IGA 4	12	PI	n.r.	mean absolute change in IL: -29.4	P< 0.0001	T + D > vehicle + Placebo	treatment related AE: 13.5 % (18/133)	10	Trifarotene + doxycycline = vehicle + placebo
	vehicle cream + doxycycline placebo	69				n.r.	mean absolute change in IL: -19.5			treatment related AE: 15.9 % (11/69)	4	
Summary efficacy: comparable efficacy once-daily trifarotene 50 µg/g + Doxycycline 120 mg = vehicle + Placebo, 1 RCT												
**Summary safety**: comparable safety once-daily trifarotene 50 µg/g + Doxycycline 120 mg = vehicle + Placebo, 1 RCT												
**Clascoterone vs. Placebo**												
Hebert 2020[5] CB-03-01/25 (face)	1% clascoterone cream; BID	353	IGA 3 or 4	12	PI	44.8	P=0.005	-	1% clascoterone cream = vehicle cream	Patients with ≥1 TEAE: 40/353 (11.3%)	66	Clascoterone = Vehicle
	vehicle cream	355				36.5				Patients with ≥1 TEAE: 41/355 (11.5%)	65	
Hebert 2020[5] CB-03-01/26 (face)	1% clascoterone cream; BID	369	IGA 3 or 4	12	PI	46.9	P<0.001	-	1% clascoterone cream > vehicle cream	Patients with ≥1 TEAE: 42/369 (11.4%)	67	Clascoterone = Vehicle
	vehicle cream	363				29.6				Patients with ≥1 TEAE: 50/363 (13.8%)	81	

New Topicals													
**Author(s)**	**Intervention**	**N**	**S**	**D**	**B**	**%↓IL**	**other Outcomes/ statistics**	**Comments**	**Summary of efficacy**	**Safety/tolerabilty**	**Drop outs**	**Summary of safety**	
Mazzetti 2019[6] (face)	clascoterone 0.1% cream; BID	72	IGA 2 - 4	12	PI	n.r.	median [range]: - 11 [-31 to +43]	only absolute Change, Inflammatory Lesions at Week 12 vs. Baseline reported<br><br>clascoterone 1% BID group significantly greater decrease (P<0.05) than clascoterone 0.5% BID, clascoterone 1% QD, and vehicle groups.		Number of Subjects with AE: 18 (25.0%)	59	Clascoterone = Vehicle	
	clascoterone 0.5% cream; BID	76				n.r.	median [range]: -7.5 [-23 to +32]			Number of Subjects with AE: 29 (38.2%)			
	clascoterone 1% cream; QD	70				n.r.	median [range]: -8.5 [-45 to +25]			Number of Subjects with AE: 16 (22.9%)			
	clascoterone 1% cream; BID	70				n.r.	median [range]: -13.5 [-39 to +38]			Number of Subjects with AE: 13 (18.6%)			
	vehicle cream BID or QD	75				n.r.	median [range]: -8.0 [-50 to +34]			Number of Subjects with AE: 17 (22.7%)			
Trifu 2011[7] (face)	Cortexolone 17a-propionate (CB-03-01) 1% cream, QD	30	IGA 2 - 3	8	PI	67.26 ± 32.03	CB-03-01 significantly more effective than placebo at week 8 (27.9%, 95% CI 5.85–49.82%, P =0.0134)<br><br>CB-03-01 was statistically better than Tretinoin at **week 6** (19.2%, 95% CI 1.13–37.32%, P =0.0374)	NIL not reported	Cortexolone 17a-propionate (CB-03-01) 1% cream, QD > Tretinoin 0.05% cream, QD AND Vehicle cream, QD	pts. with any AEs: 3/28 (11%)	10	Insufficient data	
	Tretinoin 0.05% cream, QD	32				50.71 ± 34.46				pts. with any AEs: 2/30 (7%)			
	Vehicle cream, QD	15				38.98 ± 33.22				pts. with any AEs: 3/14 (21%)			

Summary efficacy:
superior efficacy 1% clascoterone BID > vehicle (range reduction IL: Clascoterone: 46.9-67.26 %, vehicle: 29.6.3-38.98%), 2 RCT
comparable efficacy 1% clascoterone BID= vehicle, 1 RCT

New Topicals												
Author(s)	Intervention	N	S	D	B	%↓IL	other Outcomes/ statistics	Comments	Summary of efficacy	Safety/tolerabilty	Drop outs	Summary of safety
**Summary safety:** Clascoterone 1% showed comparable safety to vehicle cream												

Abbreviations: N= number, S = severity of acne, D= duration, B= Blinding, IL= inflammatory lesion

Additional safety aspects

Author	intervention	original study/studies	additional aspect	aim	outcome	results	comment
Bhatia 2024[8]	Clascoterone cream 1%, BID<br><br>study 1: entire face, shoulders, upper chest, and upper back<br>study 2: entire face and trunk	NCT01831960<br>NCT02720627	pooled analysis	laboratory signs of hypothalamic-pituitary-adrenal (HPA) axis suppression without clinical signs	cosyntropin stimulation test (CST) at baseline and day 14, if abnormal follow up 4 wks later	**day 14:** 5/65 clascoterone-treated patients with abnormal CST:<br>- 1 adult patient (Study 1, Cohort 1),<br>- 2 adolescent patients ≥12 to <18 y (Study 1, Cohort 2),<br>- 2 adolescent patients 9 to <12 y (Study 2).<br>--> all patients female<br>--> all patients white<br>**4 wks later:** All patients with normal cortisol levels	**original studies: open-label Phase-2:**<br>- n= 69 enrolled<br>- n= 65 evaluable for HPA suppression<br>**age:**<br>- study 1, cohort 1: ≥18 y<br>- study 1, cohort 2: ≥12 - <18 y<br>- study 2: 9 - <12 y<br>**IGA:**<br>- Grade 3 or 4
Eichenfield 2020[9]	Clascoterone cream 1%, BID	CB-03-01/25<br>CB-03-01/26	open-label, long-term extension study	long term safety (up to 9 mo)	AE /SAE	**pts. with any TEAE:**<br>clascoterone: 58/317 [18.3%]<br>vehicle: 52/290 [17.9%]<br><br>**Any serious TEAE:**<br>clascoterone: 3/317 [0.9%]<br>vehicle: 3/290 [1.0%]<br><br>**Any test article-related TEAE*:**<br>clascoterone: 12/317 [3.8%]<br>vehicle: 2/290 [0.7%]<br><br>**Any serious test article-related TEAE:**<br>clascoterone: 0/317 [0%]<br>vehicle: 0/290 [0%]<br><br>**Nota bene:** Study participants were summarized according to the original test article they actually received in the original study.	

Risk of bias appraisal

Reference	Outcome	Judgement for "randomization process"	Judgement for "deviations from the intended interventions (assignment)"	Judgement for "missing outcome data"	Judgement for "measurement of the outcome"	Judgement for "selection of the reported result"	Overall risk of bias judgement
Schleicher S., et al. Trifarotene Reduces Risk for Atrophic Acne Scars: Results from A Phase 4 Controlled Study. Dermatology And Therapy. 2023.13(12):3085-3096.	IL- and NIL-%-change at 24 weeks	some concerns	low risk of bias	high risk of bias	low risk of bias	some concerns	**high risk of bias**
	investigator global assessment (IGA, 5-point scale with 0 = clear to 4 = severe) at 24 weeks			high risk of bias	low risk of bias		**high risk of bias**
	safety (AE and local tolerability) at 24 weeks			high risk of bias	low risk of bias		**high risk of bias**
Alexis A., et al. Importance of treating acne sequelae in skin of color: 6-month phase IV study of trifarotene with an appropriate skincare routine including UV protection in acne-induced post-inflammatory hyperpigmentation. International Journal of Dermatology. 2024.63(6):806-815.	IL- and NIL-%-change at 24 weeks	some concerns	some concerns	high risk of bias	some concerns	some concerns	**high risk of bias**
	safety (AE and local tolerability) at 24 weeks			high risk of bias	some concerns		**high risk of bias**
Del Rosso J. Q., et al. A Randomized, Controlled Trial of Trifarotene Plus Doxycycline for Severe Acne Vulgaris. The Journal of Clinical & Aesthetic Dermatology. 2022.15(7):E53-E59	IL- and NIL-%-change at 24 weeks	low risk of bias	low risk of bias	some concerns	low risk of bias	some concerns	**some concerns**
	safety (AE and local tolerability) at 24 weeks			some concerns	low risk of bias		**some concerns**
	Quality of life			some concerns	low risk of bias		**some concerns**
Trifu V. Cortexolone 17alpha-propionate 1% cream, a new potent antiandrogen for topical treatment of acne vulgaris. A pilot randomized, double-blind comparative study vs. placebo and tretinoin 0.05% cream.	TLC; ILC after 8 wks	some concerns	low risk of bias	low risk of bias	some concerns	some concerns	**high risk of bias**
	safety: local and systemic tolerability after 8 wks		some concerns	low risk of bias	low risk of bias		**high risk of bias**

Reference	Outcome	Judgement for "randomization process"	Judgement for "deviations from the intended interventions (assignment)"	Judgement for "missing outcome data"	Judgement for "measurement of the outcome"	Judgement for "selection of the reported result"	Overall risk of bias judgement
British Journal of Dermatology. Jul 2011;165(1):177-83.							
Mazzetti A. A Phase 2b, Randomized, Double-Blind Vehicle Controlled, Dose Escalation Study Evaluating Clascoterone 0.1%, 0.5%, and 1% Topical Cream in Subjects With Facial Acne. Journal of Drugs in Dermatology: JDD. Jun 01 2019;18(6):570.	IL- and NIL median absolute change at 12 weeks	some concerns	some concerns	some concerns	some concerns	some concerns	**high risk of bias**
	participant satisfaction at 12 weeks	some concerns	some concerns	some concerns	some concerns	some concerns	**high risk of bias**
	AEs up to week 12	some concerns	some concerns	some concerns	Outcome 1/2/3: some concerns<br><br>Outcome 4/6: low risk	some concerns	**high risk of bias**
Hebert 2020 (CB-03-01/25)	percentage change from baseline in TLC, NILC, ILC at week 12	low risk of bias	low risk of bias	low risk of bias	low risk of bias	low risk of bias	**low risk of bias**
	AE at week 12	low risk of bias	low risk of bias	low risk of bias	low risk of bias	low risk of bias	**low risk of bias**
Hebert 2020 (CB-03-01/26)	percentage change from baseline in TLC, NILC, ILC at week 12	low risk of bias	low risk of bias	low risk of bias	low risk of bias	low risk of bias	**low risk of bias**
	AE at week 12	low risk of bias	low risk of bias	low risk of bias	low risk of bias	low risk of bias	**low risk of bias**
Tan J. 2019 PERFECT 1	IL; NIL at week 12	low risk of bias	low risk of bias	high risk of bias	low risk of bias	some concerns	**high risk of bias**
	safety (wk not specified)	low risk of bias	low risk of bias	high risk of bias	low risk of bias	some concerns	**high risk of bias**
Tan J. 2019 PERFECT 2	IL; NIL at week 12	low risk of bias	low risk of bias	some concerns	low risk of bias	some concerns	**high risk of bias**
	safety (wk not specified)	low risk of bias	low risk of bias	high risk of bias	low risk of bias	some concerns	**high risk of bias**

References

1. Tan J, Thiboutot D, Popp G, et al. Randomized phase 3 evaluation of trifarotene 50 mug/g cream treatment of moderate facial and truncal acne. Clinical Trial, Phase III. *Journal of the American Academy of Dermatology*. Jun 2019;80(6):1691-1699. doi:https://dx.doi.org/10.1016/j.jaad.2019.02.044
2. Schleicher S, Moore A, Rafal E, et al. Trifarotene Reduces Risk for Atrophic Acne Scars: Results from A Phase 4 Controlled Study. *Dermatol Ther (Heidelb)*. Dec 2023;13(12):3085-3096. doi:https://dx.doi.org/10.1007/s13555-023-01042-7
3. Alexis A, Del Rosso JQ, Forman S, et al. Importance of treating acne sequelae in skin of color: 6-month phase IV study of trifarotene with an appropriate skincare routine including UV protection in acne-induced post-inflammatory hyperpigmentation. *International journal of dermatology*. Jun 2024;63(6):806-815. doi:https://dx.doi.org/10.1111/ijd.17189
4. Del Rosso JQ, Johnson SM, Schlesinger T, et al. A Randomized, Controlled Trial of Trifarotene Plus Doxycycline for Severe Acne Vulgaris. *J Clin Aesthet Dermatol*. Jul 2022;15(7):E53-E59.
5. Hebert A, Thiboutot D, Stein Gold L, et al. Efficacy and Safety of Topical Clascoterone Cream, 1%, for Treatment in Patients With Facial Acne: Two Phase 3 Randomized Clinical Trials. *JAMA dermatology*. 06 01 2020;156(6):621-630. doi:https://dx.doi.org/10.1001/jamadermatol.2020.0465
6. Mazzetti A, Moro L, Gerloni M, Cartwright M. A Phase 2b, Randomized, Double-Blind Vehicle Controlled, Dose Escalation Study Evaluating Clascoterone 0.1%, 0.5%, and 1% Topical Cream in Subjects With Facial Acne. *Journal of Drugs in Dermatology: JDD*. Jun 01 2019;18(6):570.
7. Trifu V, Tiplica GS, Naumescu E, Zalupca L, Moro L, Celasco G. Cortexolone 17alpha-propionate 1% cream, a new potent antiandrogen for topical treatment of acne vulgaris. A pilot randomized, double-blind comparative study vs. placebo and tretinoin 0.05% cream. *British Journal of Dermatology*. Jul 2011;165(1):177-83. doi:https://dx.doi.org/10.1111/j.1365-2133.2011.10332.x
8. Bhatia N, Eichenfield LF, Mazzetti A, Moro L, Squittieri N, Hebert AA. Hypothalamic-Pituitary-Adrenal Axis Response in Patients With Acne Vulgaris Treated With Clascoterone. *Journal of Drugs in Dermatology: JDD*. Jun 01 2024;23(6):433-437. doi:https://dx.doi.org/10.36849/JDD.7997
9. Eichenfield L, Hebert A, Gold LS, et al. Open-label, long-term extension study to evaluate the safety of clascoterone (CB-03-01) cream, 1% twice daily, in patients with acne vulgaris. *Journal of the American Academy of Dermatology*. Aug 2020;83(2):477-485. doi:https://dx.doi.org/10.1016/j.jaad.2020.04.087
